# Physiology and Transcriptional Analysis of (p)ppGpp-Related Regulatory Effects in *Corynebacterium glutamicum*

**DOI:** 10.3389/fmicb.2019.02769

**Published:** 2019-11-28

**Authors:** Matthias Ruwe, Marcus Persicke, Tobias Busche, Benjamin Müller, Jörn Kalinowski

**Affiliations:** ^1^Microbial Genomics and Biotechnology, Center for Biotechnology, Bielefeld University, Bielefeld, Germany; ^2^Biofidus AG, Bielefeld, Germany

**Keywords:** (p)ppGpp, stringent response, stress response, promoter, discriminator, Rel

## Abstract

The alarmone species ppGpp and pppGpp are elementary components of bacterial physiology as they both coordinate the bacterial stress response and serve as fine-tuners of general metabolism during conditions of balanced growth. Since the regulation of (p)ppGpp metabolism and the effects of (p)ppGpp on cellular processes are highly complex and show massive differences between bacterial species, the underlying molecular mechanisms have so far only been insufficiently investigated for numerous microorganisms. In this study, (p)ppGpp physiology in the actinobacterial model organism *Corynebacterium glutamicum* was analyzed by phenotypic characterization and RNAseq-based transcriptome analysis. Total nutrient starvation was identified as the most effective method to induce alarmone production, whereas traditional induction methods such as the addition of serine hydroxamate (SHX) or mupirocin did not show a strong accumulation of (p)ppGpp. The predominant alarmone in *C. glutamicum* represents guanosine tetraphosphate, whose stress-associated production depends on the presence of the bifunctional RSH enzyme Rel. Interestingly, in addition to ppGpp, another substance yet not identified accumulated strongly under inducing conditions. A *C. glutamicum* triple mutant (Δ*rel*,Δ*relS*,Δ*relH*) unable to produce alarmones [(p)ppGpp^0^ strain] exhibited unstable growth characteristics and interesting features such as an influence of illumination on its physiology, production of amino acids as well as differences in vitamin and carotenoid production. Differential transcriptome analysis using RNAseq provided numerous indications for the molecular basis of the observed phenotype. An evaluation of the (p)ppGpp-dependent transcriptional regulation under total nutrient starvation revealed a complex interplay with the involvement of ribosome-mediated transcriptional attenuation, the stress-responsive sigma factors σ^B^ and σ^H^ and transcription factors such as McbR, the master regulator of sulfur metabolism. In addition to the differential regulation of genes connected with various cell functions, the transcriptome analysis revealed conserved motifs within the promoter regions of (p)ppGpp-dependently and independently regulated genes. In particular, the representatives of translation-associated genes are both (p)ppGpp-dependent transcriptionally downregulated and show a highly conserved and so far unknown TTTTG motif in the −35 region, which is also present in other actinobacterial genera.

## Introduction

Bacteria react to drastically changing environmental conditions and various stresses with a coordinated cellular response to ensure their survival. The cellular reaction, also known as stringent response, is coordinated by the hyperphosphorylated guanosine derivatives guanosine pentaphosphate (pppGpp) and guanosine tetraphosphate (ppGpp), also referred to as alarmones or (p)ppGpp (Cashel and Kalbacher, 1970; Haseltine et al., 1972). Under stressful conditions like nutrient deficiency (p)ppGpp synthesis is triggered in different ways, which are only partly known up to now (Ronneau and Hallez, 2019). Representatives of (p)ppGpp synthesizing and degrading enzymes, which are also referred to as RelA-SpoT homologs (RSHs) according to the enzymes found in *Escherichia coli*, are conserved over almost all bacterial phyla, excluding only the PVC superphylum, and represent the key player enzymes in alarmone production and degradation (Atkinson et al., 2011; Hauryliuk et al., 2015). Based on the analysis of the two eponymous gene variants of the model organism *E. coli* it has long been assumed that the (p)ppGpp metabolism of all bacteria is based solely on large RSH enzymes with (p)ppGpp synthesis as well as hydrolase domains (Chatterji and Kumar Ojha, 2001). However, new insights found by bioinformatic analyses and subsequent *in vitro* characterization resulted in an extension of the (p)ppGpp metabolism to include monofunctional (p)ppGpp synthetases [small alarmone synthase (SAS)] and monofunctional (p)ppGpp hydrolases [small alarmone hydrolase (SAH)] (Lemos et al., 2007; Nanamiya et al., 2008; Atkinson et al., 2011; Ruwe et al., 2018). Accumulation of (p)ppGpp causes a transcriptional switch, whereby growth-associated gene functions, such as ribosomal proteins and rRNA genes, are generally downregulated and survival-associated genes are upregulated (Dalebroux and Swanson, 2012; Gaca et al., 2015a). In addition, the alarmones interact with numerous proteins and thus have a massive effect on essential cellular processes such as GTP biosynthesis, replication, and translation (Srivatsan and Wang, 2008). According to recent findings, the (p)ppGpp basal levels also have considerable physiological relevance under conditions of balanced growth (Gaca et al., 2013).

In the case of the actinobacterial model organism *Corynebacterium glutamicum*, which is used for a wide variety of biotechnological production processes, a rather simple (p)ppGpp metabolism with the bifunctional RSH protein Rel as the sole component was assumed originally. A partial deletion of the corresponding gene resulted in a mutant phenotype with clear growth defects and absence of (p)ppGpp production after induction of amino acid deficiency (Wehmeier et al., 1998; Tauch et al., 2001). Later and based on bioinformatic analyses, two further components of the (p)ppGpp metabolism were identified and characterized *in vitro* for *C. glutamicum*: the SAS enzyme RelS and the SAH enzyme RelH (Ruwe et al., 2017, 2018). Growth characterization of corresponding deletion mutants revealed clear differences between the *rel* single deletion mutant and mutants lacking both (p)ppGpp producing enzymes Rel and RelS (Ruwe et al., 2018). This raises the question to what extent the newly identified enzymes RelS and RelH are involved in (p)ppGpp production or depletion under stress or balanced growth conditions. Furthermore, the synthesis of GMP 3′-diphosphate (pGpp) from GMP and ATP was demonstrated for the SAS enzyme RelS from *C. glutamicum* by assay-based *in vitro* characterization (Ruwe et al., 2017). The biological activity of this substance has already been determined by Gaca et al. (2015b). Although the presence of pGpp, as well as its isomeric structure ppGp, was already identified decades ago for *Bacillus subtilis* in response to *O*-methylthreonine-induced isoleucine starvation (Nishino et al., 1979), the analysis of these nucleotide compounds still represents a major challenge. Since pGpp, ppGp, and GTP (pppG) differ only in their phosphate group positions, they exhibit similar retention behavior in many chromatographic separation methods and are difficult to differentiate with modern mass spectroscopic methods due to their identical molecular weight. Therefore, recent investigations are usually limited to the easily separable components ppGpp and pppGpp and the analysis of a possible biological function of pGpp requires further investigation.

The *in vivo* analysis of cellular processes influenced by (p)ppGpp revealed highly variable molecular mechanisms of action depending on the living conditions of the respective species (Boutte and Crosson, 2013). Also considering the recently identified importance of the (p)ppGpp basal level for cell homeostasis, the analysis of the (p)ppGpp-mediated transcriptional regulation is of great interest for a deeper understanding of central metabolism regulation and stress response. As a result, the corresponding processes could also have a massive influence on production processes. An *in silico* study on the identification of regulatory active targets for the optimization of production processes revealed the *rel* gene from *C. glutamicum* acting as a possible regulatory function for amino acid and lycopene production (Koduru et al., 2018).

The analysis of the molecular effect mechanisms of (p)ppGpp at the transcriptional level has revealed two major mechanism of action paradigms to date: the *E. coli* paradigm and the *B. subtilis* paradigm (Prusa et al., 2018). Furthermore, an apparently less common variant of transcription initiation regulation was recently identified for the pathogenic species *Francisella tularensis* (Cuthbert et al., 2017). Although *E. coli* represents a special case regarding its RSH enzyme configuration with two long RSH copies, this Gammaproteobacterium has developed into a representative of the proteobacteria due to its historical significance. In these organisms, ppGpp and pppGpp bind to the RNA-polymerase (RNAP) and directly alter the transcription by influencing the intrinsic properties of this multi-enzyme complex (Ross et al., 2016). According to current scientific knowledge direct binding of (p)ppGpp to RNAP does not occur in most other bacterial species, with *B. subtilis* (Firmicutes) being the best characterized representative of this second paradigm. Here the transcription is influenced indirectly by a (p)ppGpp-associated change in the concentration of GTP as initial NTP (iNTP) (Krásný and Gourse, 2004; Gaca et al., 2015a). Interestingly, properties that do not correspond to the previous taxonomic picture of the two-paradigm based model were identified for *Mycobacterium tuberculosis* (gram-positive, Actinobacteria) a species taxonomically closely related to *C. glutamicum*. In contrast to other representatives of Actinobacteria such as *Cellulomonas gilvus* and *Streptomyces coelicolor*, a direct influence of pppGpp on RNAP was observed for *M. tuberculosis* by means of *in vitro* transcription analysis (Tare et al., 2013; Liu et al., 2015). The special position of *M. tuberculosis* is underlined by a regulation of the guanylate kinase (GMK) enzyme activity that differs from other gram-positive bacteria (Prusa et al., 2018). The investigation of this functional relationship, which has not yet been clarified for *C. glutamicum*, is therefore of great scientific interest in order to further investigate the evolutionary development of (p)ppGpp-related regulation processes within the phylum Actinobacteria.

Important information for the analysis of (p)ppGpp-associated effects can be generated by differential transcriptome analysis of so-called (p)ppGpp^0^ strains, which can no longer synthesize alarmone species by deletion of RSH genes. Studies for *E. coli* carried out under different stress conditions revealed differential regulation of 700–800 genes between the (p)ppGpp-devoid strain and the parental strain, thus illustrating the global influence of (p)ppGpp on transcriptional regulation (Durfee et al., 2008; Traxler et al., 2008). However, the induction of alarmone production is a decisive factor, especially considering the various RSH enzyme configurations (Gourse et al., 2018). The different induction strategies such as the artificial induction of an amino acid deficiency by amino acid analogs like SHX or the induction of a deficiency situation result in different metabolic changes. In addition to the effects directly caused by (p)ppGpp, this leads to further changes in the transcriptome, which cannot be distinguished from each other. Therefore, both the individual properties of the respective (p)ppGpp metabolism and the suitability of the induction strategy used must be considered when carrying out transcriptome analyses. Recently, Sanchez-Vazquez et al. (2019) presented a system for stress-independent analysis of (p)ppGpp-related effects based on the induced production of a constitutively active RelA enzyme and an RNAP variant without (p)ppGpp binding sites. However, this system is difficult to transfer to other organisms.

In the context of the study presented here, a global transcriptome analysis was performed to investigate (p)ppGpp-associated effects for *C. glutamicum*, taking the above-mentioned constraints into account. The influence of the (p)ppGpp basal level on the physiology of *C. glutamicum* was investigated by phenotypic and transcriptional comparison of the parental strain CR099, a lab strain free of prophages and the two most abundant insertion elements IS*Cg1* and IS*Cg2*, with the derived (p)ppGpp^0^ mutant strain CR099 Δ*rel*Δ*relS*Δ*relH.* Furthermore, the induction of alarmone production was investigated by ^32^P-labeling and thin-layer chromatography (TLC) analysis of the nucleotide spectrum under different stress conditions. Based on the optimized induction conditions, a differential RNAseq analysis was performed to determine the (p)ppGpp-associated effects in the context of the stress response. Particular attention was paid to the investigation of possible DNA signatures involved in the influence of (p)ppGpp. Corresponding sequence motifs or characteristics have already been found by *in vitro* transcription analysis of individual promotors for organisms from different taxonomic branches such as *E. coli*, *B. subtilis*, and *M. tuberculosis* (Tare et al., 2013). The high resolution of RNAseq, together with available data including transcription start sites and promoter investigations, allows a global sequence analysis and thus delivers more valid information on the stress- and basal level-associated function of alarmones.

## Materials and Methods

### Bacterial Strains and Growth Condition

Bacterial strains used for this study are listed in Table 1.

**TABLE 1:** Bacterial strains used in this study.

**Strain**	**Characteristics or genotype**	**Source or references**
*C. glutamicum*		
CR099	*C. glutamicum* ATCC 13032, ΔCGP1, ΔCGP2, ΔCGP3, Δ*ISCg*1, Δ*ISCg*2	Baumgart et al., 2013; Unthan et al., 2015
CR099 Δ*crtR*	*C. glutamicum* CR099 Δ*crtR*	This work
CR099 Δ*rel*	*C. glutamicum* CR099 Δ*rel*	Ruwe et al., 2017
CR099 Δ*relS*	*C. glutamicum* CR099 Δ*relS*	Ruwe et al., 2017
CR099 Δ*relH*	*C. glutamicum* CR099 Δ*relH*	Ruwe et al., 2018
CR099 Δ*rel*Δ*relS*	*C. glutamicum* CR099 Δ*rel*Δ*relS*	Ruwe et al., 2017
CR099 Δ*rel*Δ*relH*	*C. glutamicum* CR099 Δ*rel*Δ*relH*	Ruwe et al., 2018
CR099 Δ*relS*Δ*relH*	*C. glutamicum* CR099 Δ*relS*Δ*relH*	Ruwe et al., 2018
CR099 Δ*rel*Δ*relS*Δ*relH*	*C. glutamicum* CR099 Δ*rel*Δ*relS*Δ*relH*	Ruwe et al., 2018
CR099 Δ*rel*Δ*relS*Δ*relH*Δ*crtR*	*C. glutamicum* CR099 Δ*rel*Δ*relS*Δ*relH*Δ*crtR*	this work
*E. coli*		
MG1655	Wild-type *E. coli* MG1655, derived from *E. coli* K12	Blattner et al., 1997

Cultivation of *C. glutamicum* strains was carried out as described previously (Ruwe et al., 2017). MOPS Minimal Medium (MMM) with a glucose concentration of 4 g/L was used for the cultivation of *E. coli* MG1655 (Neidhardt et al., 1974). In order to obtain a strong ^32^P signal during nucleotide measurements, the phosphate concentration in CGXII medium (Keilhauer et al., 1993) was reduced to a final concentration of 1.32 mM (low P). Furthermore, all 20 proteinogenic amino acids were supplemented to a final concentration of 50 mg/L each to allow TLC-based analysis of various stressors and deletion mutants in defined nutrient-rich conditions comparable to previous measurements (Durfee et al., 2008; Nanamiya et al., 2008; Gaca et al., 2012; Sanchez-Vazquez et al., 2019). The cultivations under light and dark conditions as well as for transcriptome measurements were also carried out in CGXII medium with supplementation of all canonical amino acids (50 mg/L each). To illuminate the cultures, two 18 W cool white fluorescent tubes were mounted in the shaker above the shaking flasks.

### Deletion of *crtR* in *C. glutamicum* Strains

The construction of *C. glutamicum crtR* deletion mutants was performed as described previously (Ruwe et al., 2017), based on the suicide vector system pK19*mobsacB* (Schäfer et al., 1994). The construction of the plasmid pK19*mobsacB*-*crtR* used was described by Henke et al. (2016). The system is based on the in-frame deletion of the target gene by a two-step homologous recombination. For this purpose, the flanking regions of the target gene were cloned into the vector pK19*mobsacB* and the recombination events were triggered by appropriate selection conditions.

### ^32^P-Based Nucleotide Measurements

Precultures of the investigated strains were cultured in the main culture medium overnight. The main cultures were inoculated with an optical density of 0.15. 396 μL of each culture and 4 μL of a 5.4 mCi/mL [^32^P]phosphoric acid solution (Hartmann Analytic, Braunschweig, Germany) were transferred into 1.5 mL reagent vessels with a punctured lid. Cultivation was performed for 3 h at 30°C and 1000 rpm using a Thermomixer comfort shaker (Eppendorf, Hamburg, Germany). The ^32^P-labeled cultures were treated with stressors as specified in the figure legends or centrifuged down for 5 s at 16,000 × *g* and resuspended in starvation solution (42 g/L MOPS, 250 mg/L MgSO_4_ × 7H_2_0, pH 7.0) as indicated. Subsequently, the samples were further incubated for 30 min. For cell disruption, the cultures were centrifuged for 5 s at 16,000 × *g*, the obtained cell pellet was resuspended in 20 μL 6 M formic acid and immediately frozen in liquid nitrogen. five freeze-thaw cycles were performed, and the samples were centrifuged for 5 min at 16,000 × *g*. 1.5 μL of the obtained solutions were applied to PEI cellulose F TLC plates (Merck, Darmstadt, Germany). The enzymatic preparation of the [^32^P](pp)pGpp running marker was carried out in 50 μL reaction mixtures containing 5 mM MgCl_2_, 1 mM GMP/GDP/GTP each, 3.5 μM purified RelS (Ruwe et al., 2017) and 0.5 μL of a 10 mCi/mL [γ-^32^P]ATP solution in 50 mM HEPES buffer (pH 7). Following an incubation of 3 h at room temperature, 1450 μL of a 25 mM HEPES buffer solution (pH 7.0) with 5 mM MgCl_2_ were added to the reaction mixture and 0.4 μL of the diluted (pp)pGpp solution was applied as the reference to the TLC plate. The TLC plates were developed using 1.5 M KH_2_PO_4_ solution (pH 3.4) (Cashel and Kalbacher, 1970), exposed on a storage phosphor screen for 24 h (GE Healthcare, Chicago, IL, United States) and visualized with a Typhoon 8600 imager (GE Healthcare, Chicago, IL, United States).

### Transcriptome Measurements

For transcriptome analysis, both 42 mL of the parental strain CR099 and the (p)ppGpp^0^ mutant CR099 Δ*rel*Δ*relS*Δ*relH* were cultured in 250 mL shaking flasks in three biological replicates with an initial optical density of 0.4. After reaching an OD_600_ value of 2.5, 2 mL of the cell suspension was harvested by rapid centrifugation, supernatant removal, and immediate shock freezing in liquid nitrogen. For transcriptome analysis under starvation conditions, the cultures were transferred to 50 mL tubes after collecting the unstressed t_0_ sample and centrifuged immediately for 10 s at 10,000 × *g*. The resulting pellets were directly resuspended in 40 mL of starvation solution and further incubated under identical conditions. After 5, 15, 30, and 60 min, respectively, additional sampling was performed. For isolation of total RNA, the RNeasy Mini Kit was used along with the RNAse free DNA Kit (Qiagen, Hilden, Germany), according to Hüser et al. (2003). In the process, genomic DNA was digested in three successive reactions, two times on-column and once in solution. Ribosomal RNA was depleted using the Ribo-Zero rRNA Removal Kit (Bacteria) (Illumina, San Diego, CA, United States). Library preparation was performed with the TruSeq Stranded Total RNA Library Prep Kit (Illumina, San Diego, CA, United States) using the manufacturer’s specifications. For sequencing of 2 × 25 bp or 2 × 70 bp (paired end reaction) the Illumina HiSeq 1500 platform (San Diego, CA, United States) was used in rapid mode. The trimmed reads were mapped to a *C. glutamicum* CR099 reference genome using *Bowtie* 2 (Langmead and Salzberg, 2012). Principle Component Analysis (PCA) was performed with *R* (R Core Team, 2018) using read counts generated by *ReadXplorer* 2.2.3 (Hilker et al., 2016) and normalized with *DESeq2* (Love et al., 2014). Differential gene expression analysis was also performed using the tool *DESeq2* (Love et al., 2014). Only genes with transcriptional changes of more than two-fold (log_2_ fold change ≥1 or ≤−1) compared to the unstressed initial state and a *p*_adj_ ≤ 0.01 were regarded as differentially transcribed. For over-representation analysis (ORA) of the differentially regulated genes with respect to their assignment to KEGG-pathways, the *DAVID* 6.7 software was used (Huang et al., 2009a, b).

### Decaprenoxanthin Measurements

The extraction and analysis of carotenoids from *C. glutamicum* was performed as described by Heider et al. (2014) and Henke et al. (2017). In summary, the pigments were extracted from the cell pellets of 1 mL aliquots of the individual cultures with 1 mL of a methanol:acetone mixture (7:3) in a Thermomixer comfort shaker (Eppendorf, Hamburg, Germany) at 60°C and 800 rpm for 20 min. HPLC analysis of the carotenoid content was performed on an Agilent 1200 series HPLC system (Agilent Technologies GmbH & Co. KG, Böblingen, Germany), including a diode array detector (DAD). For chromatographic separation 50 μL were injected into a column system (CS Chromatographie Service GmbH, Langerwehe, Germany) consisting of a precolumn (LiChrospher 100 RP18 EC-5, 40 × 4 mm) and a main column (LiChrospher 100 RP18 EC-5, 125 × 4 mm) with methanol (A) and methanol/water (9:1) (B) as mobile phases. Since no commercially available standard for decaprenoxanthin was available, its concentration was calculated by standardization with beta-carotene as beta-carotene equivalent. For the analysis of carotenoid production depending on the regulator CrtR equal cell numbers of the analyzed strains were plated on CASO-bouillon (Roth, Karlsruhe, Germany) media plates and incubated for 48 h under variable light conditions at room temperature. Subsequently, cell material was resuspended in PBS buffer and processed analogously to the cultivation samples.

### Amino Acid Measurements

The amino acid analysis of the cultivation supernatants were performed by derivatization with *o*-phthaldialdehyde (OPA) (Lindroth and Mopper, 2002) and subsequent HPLC analysis, as described by Walter et al. (2016). An OPA Precolumn Analyser system (Knauer, Berlin, Germany) was applied and the separation was performed using an AccQ-Tag^TM^ 3.9 × 150 mm column (Waters, Milford, MA, United States). The following gradient was used with a flow rate of 1 mL/min: 0 min A: 100%, 20 min A: 100%, 50 min B: 100%, 55 min B. 100%. Identification of the amino acids contained in the supernatant was based on physiological amino acid standard A9906 (Sigma-Aldrich, St. Louis, MO, United States), and quantification was carried out using single pure reference standards to obtain calibration curves.

### Vitamin Measurements

Samples were diluted 1:20 in 0.05% acetic acid and analyzed *via* LC-MS/MS in positive ionization mode. Separation of vitamins was carried out on an Acclaim C30 column 3.0 μm column (Thermo Scientific, Waltham, MA, United States) with the mobile phase consisting of eluent A water with 0.1% formic acid and eluent B acetonitrile. Injection volume was 5 μL, oven temperature was 32 °C, and flow rate was set to 200 μl min^–1^. Separation was performed using a gradient from 0 to 95% B. Liquid chromatography was interfaced to a Varian 320 MS triple quadrupole instrument *via* an electrospray ionization source using Argon as collision gas. The identification and quantification of specific B vitamins in the supernatant was accomplished by comparison with B vitamin standards using tandem mass spectrometry (MS/MS) and selected reaction monitoring (SRM). The following transitions were used: Pantothenate – 220.1 m/z/89.9 m/z, Riboflavin – 377.0 m/z/243.0 m/z. Each sample was analyzed in duplicates.

### Metabolite Measurements

Both metabolite derivatization and GC-MS analysis of the cultivation supernatants were performed as described by Plassmeier et al. (2007). The cells were removed by centrifugation for 5 min at 16,000 × *g* and subsequent filtration of the supernatant using a Filtropur S 0.2 μM pore size membrane filter (Sarstedt, Nümbrecht Germany). 40 μL of the cell-free supernatant were added 1000 μL 80% methanol (v/v) with 10 μM ribitol as internal standard and the resulting solution was completely evaporated in a nitrogen stream. Two-step derivatization of the metabolites was performed by methoxymation and silylation. For metabolite measurement, 1 μL of the derivatized samples was injected into a GC-MS system consisting of a Trace GC gas chromatograph, a PolarisQ ion trap and an AS1000 autosampler (Thermo Fisher, Dreieich, Germany). The chromatographic separation was accomplished utilizing a 30 m × 0.25 mm OPTIMA^®^ 5 MS Accent GC column with a 0.25 μm silarylene phase (Macherey-Nagel, Düren, Germany). Compound detection and normalization to the internal standard ribitol was performed as described in Persicke et al. (2011), using the *Xcalibur*^TM^ software (Version 1.4, Thermo Fisher, Dreieich, Germany) and the *MeltDB* software (Neuweger et al., 2008).

### Whole Genome Sequencing of Cultivation Replicates

Since individual replicates of strain CR099 Δ*rel*Δ*relS*Δ*relH* showed considerably different behavior in the course of phenotypic characterization, whole genome sequencing of the stationary phase cultures was performed in order to define the cause of the observed results more closely. For DNA isolation from 1 mL pelleted culture the NucleoSpin Microbial DNA Kit (Macherey-Nagel, Düren, Germany) was used. Library preparation was performed using the TruSeq DNA PCR-Free Kit (Illumina, San Diego, CA, United States). The Illumina MiSeq platform (San Diego, CA, United States) was applied for sequencing of 2 × 300 bp using the MiSeq Reagent Kit v3 (Illumina, San Diego, CA, United States). The trimmed reads were mapped to a *C. glutamicum* CR099 reference genome using *Bowtie* 2 (Langmead and Salzberg, 2012). In order to identify possible genomic changes, the SNP detection function of the tool *ReadXplorer* 2.2.3 (Hilker et al., 2016) was used. Since enriched subpopulations had to be assumed, a minimum variation of 25% was allowed, which directly corresponds to one quarter of the total cell population due to the sequencing technology used. The occurrence of genomic alterations in even smaller subpopulations was assumed to be irrelevant due to the pronounced phenotypic heterogeneity.

## Results

### Total Starvation, Antibiotic Stress and Iron Stress Lead to a Significant Accumulation of Alarmones in *C. glutamicum* and a Nucleotide Spectrum Differing From That of *E. coli*

**FIGURE 1:**
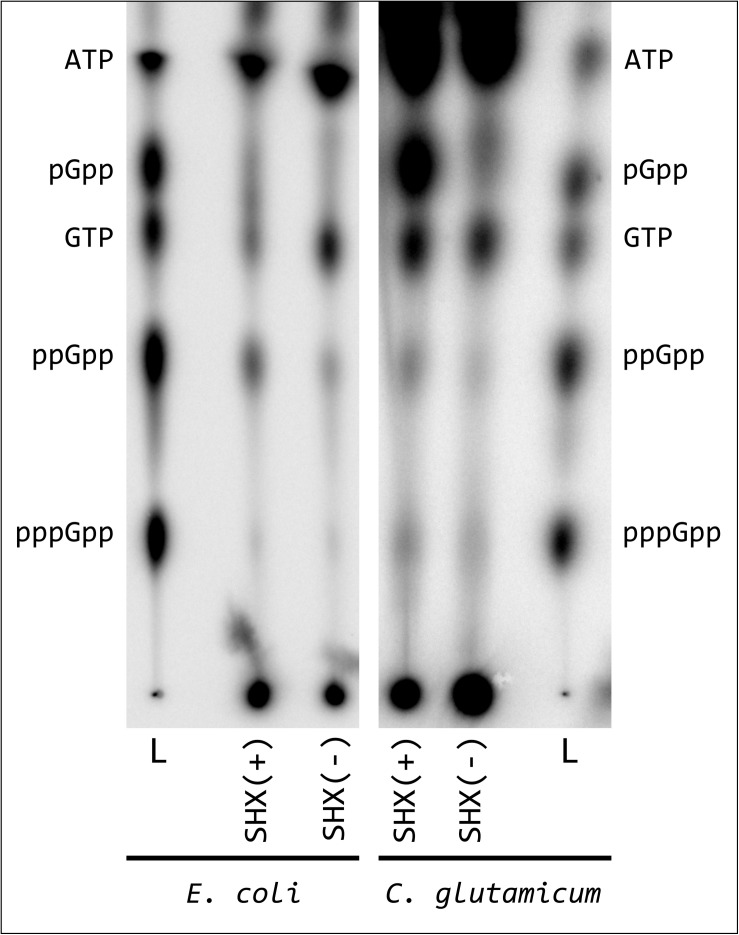
Synthesis of hyperphosphorylated nucleotides in *E. coli* and *C. glutamicum* after SHX treatment. *E. coli* cells were labeled in MMM, *C. glutamicum* cells in CGXII medium (low P) for 3 h with 54 μCi/ml [^32^P]orthophosphate. Subsequently the cultures were treated with 1 mg/L SHX and 0.5 mg/L L-valine, as described by Lemos et al. (2007) and further incubated for 30 min. For nucleotide extraction, the cell pellets from treated samples and the untreated controls were resuspended in 6 M formic acid, shock-frozen in liquid nitrogen and subjected to five freeze-thaw cycles. 1.5 μL of the clarified supernatant were applied to PEI cellulose TLC-plates and developed in 1.5 M KH_2_PO_4_ solution (pH 3.4). ^32^P-labeled pGpp, ppGpp and pppGpp standards were prepared enzymatically by RelS catalyzed pyrophosphorylation of GMP, GDP, and GTP using [γ-^32^P]ATP and applied together with [γ-^32^P]ATP and [α-^32^P]GTP.

Our first question relates to whether the classical induction of amino acid deficiency by addition of the seryl-tRNA synthetase inhibitor SHX is also useful to study alarmone production in *C. glutamicum*. Here nucleotide measurements based on ^32^P-labeling and TLC separation revealed substantial differences between *E. coli* and *C. glutamicum*. For a better interpretation of the TLC patterns, we used a alarmone standard composed of pGpp, ppGpp, and pppGpp synthesized *in vitro* with the SAS enzyme RelS (Ruwe et al., 2017). Compared to the initial state before SHX application, a significantly reduced GTP signal was observed for *E. coli* MG1655 after an induction with 1 g/L SHX and 0.5 g/L L-valine in MMM, the intensity of which correlated directly with an increase in the ppGpp signal (Figure 1). For reasons of comparability, identical induction conditions were used for initial *C. glutamicum* experiments, although valine supplementation was not expected to have any additional effect on the induction of (p)ppGpp synthesis. The addition of valine to the strain *E. coli* strain K12 causes an isoleucine deficiency and thus (p)ppGpp accumulation (Leavitt and Umbarger, 1962). The induction mechanism relies on the feedback inhibition of the acetohydroxy acid synthases AHAS I and AHAS III by valine and is specific for this strain since the non-valine inhibited isoleucine synthetase AHAS II is inactive due to a frameshift mutation in the gen *ilvG* (Felice et al., 1979). Using chemically defined CGXII-minimal medium, no change in GTP concentration was detected for *C. glutamicum* and the ppGpp signal had only a slightly increased intensity. In contrast to *E. coli*, however, *C. glutamicum* was characterized by the SHX-induced formation of a pronounced spot above the GTP signal. The migratory behavior of the corresponding substance was similar to that of pGpp. To further characterize the effects of SHX on *C. glutamicum* a growth experiment was performed. Contrary to expectations based on the behavior of several other species, the addition of 1 g/L SHX at an OD_600_ of 2.5 in CGXII medium supplemented with all 20 proteinogenic amino acids (50 mg/L each) did not result in an immediate stop of cell growth (Supplementary Figure S1). Compared to the reference the treated culture exhibited merely a slightly reduced growth rate in the exponential growth phase.

**FIGURE 2:**
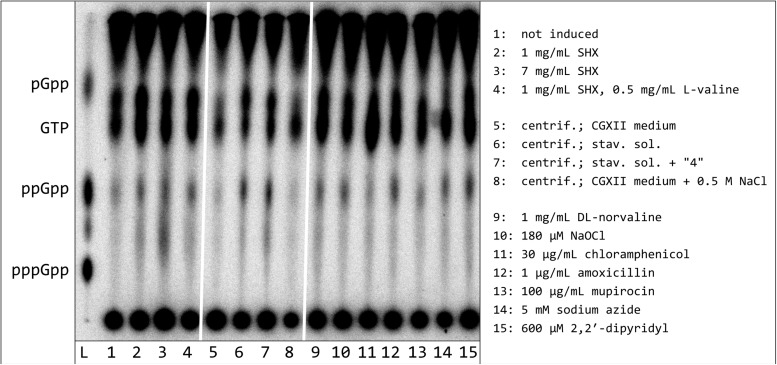
Synthesis of hyperphosphorylated nucleotides in *C. glutamicum* after treatment with various stressors or exposure to nutrient deficiency. *C. glutamicum* cells were labeled in CGXII medium (low P), containing all proteinogenic amino acids (50 mg/L each), for 3 h with 54 μCi/ml [^32^P]orthophosphate. The cultures were treated with the indicated stressors, respectively centrifuged and subsequently resuspended in starvation solutions, as specified. All samples were further incubated for 30 min. For nucleotide extraction, the cell pellets were resuspended in 6 M formic acid, shock-frozen in liquid nitrogen and subjected to five freeze-thaw cycles. 1.5 μL of the clarified supernatant were applied to PEI cellulose TLC-plates and developed in 1.5 M KH_2_PO_4_ solution (pH 3.4). ^32^P-labeled pGpp, ppGpp. and pppGpp standards were prepared enzymatically by RelS catalyzed pyrophosphorylation of GMP, GDP, and GTP using [γ-^32^P]ATP.

In order to find optimal induction conditions for alarmone synthesis in *C. glutamicum*, a spectrum of different stressors was analyzed. These treatments comprised antibiotics (amoxicillin, chloramphenicol, mupirocin), amino acid analogs (D,L-norvaline, SHX), chemical stressors (NaOCl, sodium azide, dipyridyl) as well as a removal of all carbon-, nitrogen-, and phosphorous-containing nutrients (total starvation). When interpreting the data obtained, it has to be considered that significant labeling differences are most likely to occur due to varying metabolic activities after induction or the almost complete removal of the ^32^P orthophosphate from the experimental approaches exposed to starvation stress. For this reason, no quantitative evaluation was carried out and the data were only considered qualitatively. It was shown that total nutrient deficiency, compared to all other stressors tested, led to a significantly higher ppGpp production, since the strongest ppGpp spot intensities and the maximum ratio between ppGpp and GTP were determined for incubation in starvation solution (Figure 2). While dipyridyl, amoxicillin, norvaline, NaOCl, and sodium azide induced a low but distinct ppGpp production analogous to SHX, the also frequently used inductor mupirocin as well as the ribosomal peptidyl transferase inhibitor chloramphenicol had no effect on the ppGpp signal compared to the non-induced comparative cultivation.

The spot above the GTP signal already detected during the first SHX-induction experiments, became stronger in all treatments leading to ppGpp induction. The maximum spot intensity in relation to the GTP signal was found for treatments with SHX, norvaline and under conditions of total starvation. Interestingly, in this TLC approach, the running behavior of the unknown spot clearly differed from the enzymatically produced pGpp. In addition, another spot occurred between ppGpp and pppGpp in the marker lane. As this spot also appeared in a [γ-^32^P]ATP standard track (data not shown) it obviously represents a reagent impurity.

### The Bifunctional RSH Protein Rel_Cg_ Is the Key Player Enzyme of Total Starvation Induced ppGpp Production

**FIGURE 3:**
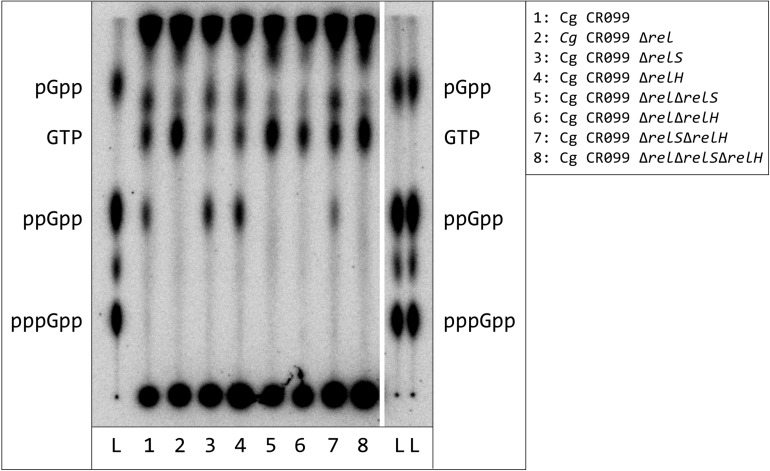
Synthesis of hyperphosphorylated nucleotides in *C. glutamicum* strains with deletions of (p)ppGpp metabolism associated genes after exposure to total starvation. *C. glutamicum* cells were labeled in CGXII medium (low P), containing all proteinogenic amino acids (50 mg/L each), for 3 h with 54 μCi/ml [^32^P]orthophosphate. The cultures were centrifuged and subsequently resuspended in starvation solution. All samples were further incubated for 30 min under constant conditions. For nucleotide extraction, the cell pellets from treated samples and the untreated controls were resuspended in 6 M formic acid, shock-frozen in liquid nitrogen and subjected to five freeze-thaw cycles. 1.5 μL of the clarified supernatant were applied to PEI cellulose TLC-plates and developed in 1.5 M KH_2_PO_4_ solution (pH 3.4). ^32^P-labeled pGpp, ppGpp, and pppGpp standards were prepared enzymatically by RelS catalyzed pyrophosphorylation of GMP, GDP, and GTP using [γ-^32^P]ATP.

Once total starvation was identified as suitable method to induce stringent response or production of ppGpp in *C. glutamicum*, respectively, an investigation of *C. glutamicum* deletion mutants in (p)ppGpp metabolism was performed. These included all deletion combinations of the genes *rel*, *relS*, and *relH*, which have already been functionally characterized by *in vitro* analyses (Ruwe et al., 2017, 2018). The evaluation of the alarmone measurements revealed that the accumulation of ppGpp only occurred in strains where *rel* was present (Figure 3). In order to determine the running behavior of the TLC separation in more detail, the marker mixture of pGpp, ppGpp, and pppGpp was applied twice in the border areas of the TLC plate. Since the individual substance spots were located exactly parallel, an asymmetrical separation behavior in the border region could be excluded.

### A *C. glutamicum* (p)ppGpp^0^ Mutant Exhibits Altered Transcript Amounts of Genes Involved in Sulfur Metabolism-, Amino Acids- and Carotenoid Synthesis

**FIGURE 4:**
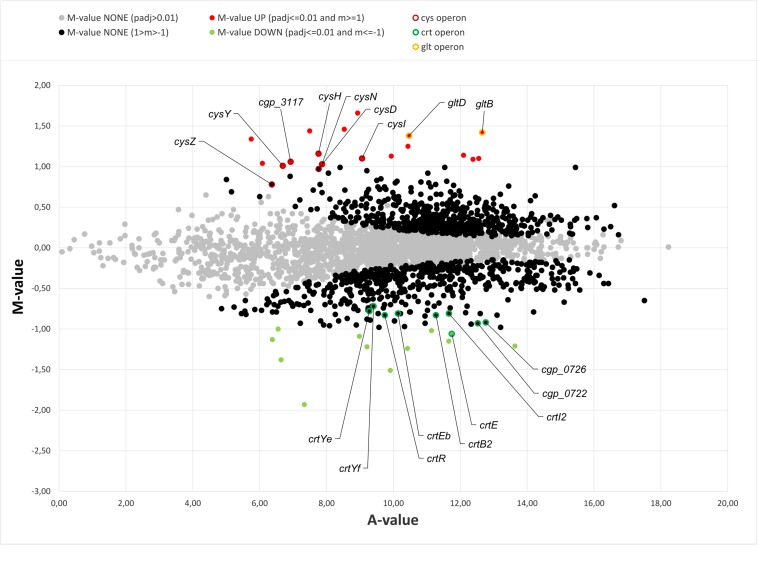
MA-plot of transcriptome data from early exponential growth phase: *C. glutamicum* CR099 Δ*rel*Δ*relS*Δ*relH* vs. *C. glutamicum* CR099. Visualization of transcriptomic data from three biological replicates each, transformed onto *M* (log_2_ fold change) and *A* (mean average) values, using *DESeq2* (Love et al., 2014). Significantly differentially transcribed genes are labeled in red (*M*: > 1; *p*_adj_: < 0.01) and green (*M*: < −1; *p*_adj_: < 0.01), respectively. Genes below the fold change threshold with a significant probability value (1 > *M* > 1; *p*_adj_: < 0.01) are visualized in black and genes with a *p*_adj_: > 0.01 in gray. Representatives of the *cys*, *crt*, and *glt* operons are highlighted by colored borders: *cys*: dark red; *crt*: dark green; *glt*: yellow.

In earlier studies the phenotypic characterization of different *C. glutamicum* mutants in (p)ppGpp metabolism revealed clear growth differences to the parental strain (Ruwe et al., 2018). Since the differences also occurred in early growth phases under good growth conditions and are therefore probably associated with the altered (p)ppGpp basal level, a differential transcriptome analysis for the early exponential growth phase was aimed at. RNAseq analysis of the parental strain CR099 and its derived (p)ppGpp^0^ mutant CR099 Δ*rel*Δ*relS*Δ*relH* was performed during growth in CGXII minimal medium with additional supplementation of all 20 proteinogenic amino acids (50 mg/L each). Comparing both strains, 17 differentially up- and 13 downregulated genes were identified for the (p)ppGpp-devoid mutant (Table 2 and Figure 4). Amongst others, *crtE*, a gene involved in carotenoid biosynthesis, *metE* coding for the final enzyme of methionine biosynthesis, and the sucrose-specific phosphotransferase system gene *ptsS* were significantly downregulated. A closer examination of the corresponding biosynthetic pathways showed that in the (p)ppGpp^0^ strain all other genes of the operon *crtE-cg0722-crtBIYEb* associated with decaprenoxanthin synthesis were also significantly downregulated with *M* values from −0.72 to −0.93. In contrast, the other genes of methionine biosynthesis did not show differential transcription. The significantly upregulated genes included representatives of the *cys* (assimilatory sulfate reduction) and *gltBD* (glutamate synthase) operons.

**TABLE 2:** Differentially transcribed genes in the early exponential growth phase: *C. glutamicum* CR099 Δ*rel*Δ*relS*Δ*relH* vs. *C. glutamicum* CR099.

**Locus tag**	**Gene name**	**Product/Function**	***M*-value**	***p*_adj_**
*cg0096*		hypothetical protein	1.04	1.93295E−06
*cg0175*		putative secreted protein	1.13	1.68562E−25
*cg0177*		hypothetical protein	1.10	1.64591E−48
*cg0229*	*gltB*	glutamate synthase (NADPH), large chain	1.42	1.30696E−08
*cg0230*	*gltD*	glutamate synthase (NADPH), small chain	1.38	3.24867E−08
*cg0706*		putative membrane protein	1.14	6.41725E−63
*cg1325*		hypothetical protein	1.25	1.64564E−61
*cg1665*		putative secreted protein	1.09	6.95957E−12
*cg1784*	*ocd*	putative ornithine cyclodeaminase	1.44	5.2046E−16
*cg2543*	*glcD*	putative (S)-2-hydroxy-acid oxidase	1.66	1.09228E−47
*cg2545*		putative secreted or membrane protein	1.34	1.85048E−09
*cg2546*		putative secondary C4-dicarboxylate transporter, tripartite ATP-independent transporter (TRAP-T) family	1.46	1.04406E−29
*cg3113*	*cysY*	sirohydrochlorin ferrochelatase	1.01	5.6237E−07
*cg3114*	*cysN*	sulfate adenylyltransferase subunit 1	1.03	4.76049E−11
*cg3116*	*cysH*	adenosine phosphosulfate reductase	1.16	2.57338E−11
*cg3117*	*cysX*	ferredoxin-like protein, involved in electron-transfer	1.06	4.3445E−08
*cg3118*	*cysI*	ferredoxin-sulfite reductase	1.10	2.31364E−15
*cg0507*		ABC-type putative spermidine/putrescine/iron(III) transporter, permease subunit	–1.09	4.43375E−17
*cg0623*		ABC-type putative cobalt transporter, permease subunit	–1.02	6.33337E−47
*cg0723*	*crtE*	geranylgeranyl pyrophosphate synthase	–1.06	4.57784E−06
*cg0921*		siderophore-interacting protein	–1.13	1.23408E−08
*cg0922*		ABC-type putative iron-siderophore transporter, substrate-binding lipoprotein	–1.38	5.29062E−13
*cg1290*	*metE*	5-Methyltetrahydropteroyltriglutamate- homocysteine methyltransferase	–1.21	3.98243E−15
*cg1485*	*relH*	(p)ppGpp phosphohydrolase, RelA/SpoT-family	–1.93	3.21735E−33
*cg1861*	*rel*	Bifunctional (p)ppGpp synthase/hydrolase, RelA/SpoT-family	–7.25	0^∗^
*cg1862*	*apt*	adenine phosphoribosyltransferase	–1.24	1.24926E−63
*cg2234*		ABC-type putative iron(III) dicitrate transporter, substrate-binding lipoprotein	–1.00	3.76317E−06
*cg2324*	*relS*	(p)ppGpp synthetase, RelA/SpoT-family	–1.51	8.38571E−77
*cg2925*	*ptsS*	phosphotransferase system (PTS), sucrose-specific enzyme IIBCA component	–1.15	7.96046E−51
*cg3226*		putative MFS-type L-lactate permease	–1.22	9.86559E−18

*log_2_ ratios (*M*-values) and probability values (*p*_*adj*_) were determined using *DESeq2* (Love et al., 2014), integrated in the *ReadXplorer* 2 software (Hilker et al., 2016), based on three biological replicates each. ^∗^Artifact due to removal of most of the respective gene from the mutants genome.*

### The (p)ppGpp^0^ Strain Exhibits Significant Metabolic Changes in Vitamin, Amino Acid and Carotenoid Biosynthesis and Shows Unstable Growth Behavior Under the Influence of Light

Based on the transcriptome data of the exponential growth phase, an analysis of the compared strains with respect to their amino acid and carotenoid production was performed as follows. Since recently a direct association between light dependence of the carotenoid biosynthesis and the *crt* operon was described (Henke et al., 2017), the analysis was performed under both light and dark conditions. After a cultivation time of 24 h, the cultures of the (p)ppGpp^0^ mutant had not yet reached the expected optical density, so cultivation was continued for another 20 h. Interestingly, even after 44 h of cultivation, the individual replicates achieved different optical densities. While the cultures of the parental strain had only minimal differences, the cultures of the (p)ppGpp-devoid strain achieved lower optical densities under illumination. Furthermore, the differences between the individual CR099 Δ*rel*Δ*relS*Δ*relH* cultures were so severe that the biological replicates were subsequently considered separately. In order to exclude genomic alterations as a possible reason for phenotypic heterogeneity, PCR-free whole genome sequencing was performed for the stationary phase cultures of the individual replicates. An SNP analysis with comparatively low variance parameters revealed that all cultures did not contain genomically modified subpopulations comprising at least 25% of the cells.

**FIGURE 5:**
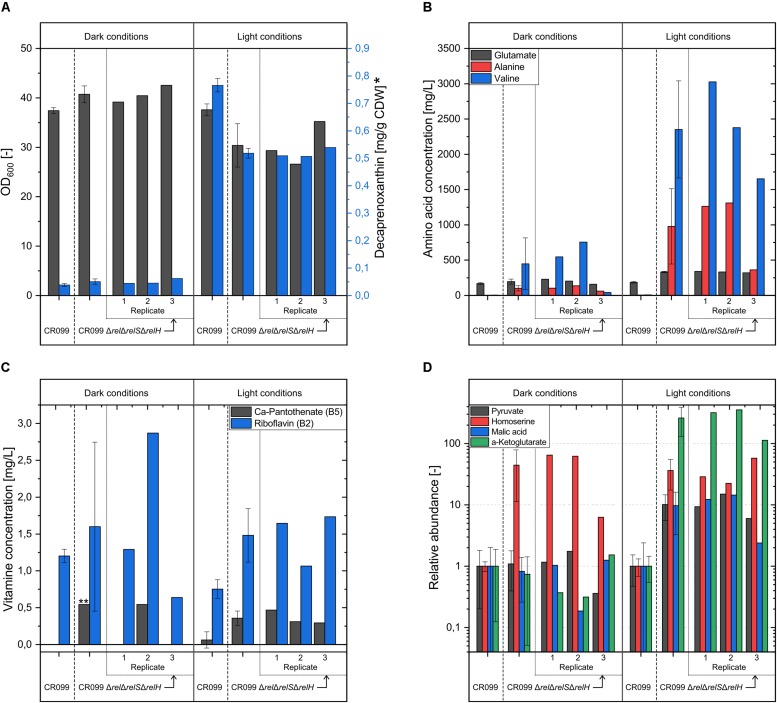
Analysis of relevant parameters for the cultivation of *C. glutamicum* CR099 and *C. glutamicum* CR099 Δ*rel*Δ*relS*Δ*relH* in dependence of illumination. Both strains were cultured in CGXII medium with supplementation of all proteinogenic amino acids (50 mg/L each) for 44 h under light and dark conditions. Due to considerable differences within the three biological replicates of the (p)ppGpp^0^ strain, the mean values as well as the corresponding individual measurements are plotted. **(A)** OD_600_ values and decaprenoxanthin concentration, determined by HPLC analysis and normalized to the cell weight used for extraction. ^∗^ Due to the unavailability of standards, the decaprenoxanthin concentration was calculated as ß-carotene equivalent. **(B)** L-glutamate, L-alanine and L-valine concentrations in supernatant, determined by OPA derivatization and HPLC measurement. **(C)** Supernatant concentration of water-soluble vitamins Ca-pantothenate and riboflavin, analyzed by LC-MS/MS measurement. ^∗∗^ Since the pantothenate concentration in two out of three replicates was below the limit of detection, only the single measured value is indicated without a corresponding error. **(D)** Concentrations of pyruvate, homoserine, malic acid, and α-ketoglutarate in supernatant, determined by methoxymation and silylation and subsequent GC-MS measurement. Metabolite values were normalized to the internal standard ribitol and illustrated as relative abundance, compared to the parental strain values.

As expected, only little amounts of the carotenoid decaprenoxanthin were produced by all cultures under dark conditions ranging in concentration from 0.034 to 0.062 mg/g CDW (Figure 5). In contrast, a significantly stronger carotenoid synthesis took place under light conditions, with the parental strain producing significantly more decaprenoxanthin than the (p)ppGpp^0^ mutant. In order to investigate the effects of (p)ppGpp on carotenoid synthesis, a possible association with the recently described regulator CrtR (Henke et al., 2017) was investigated. For this purpose, the gene *crtR* was deleted from both CR099 and CR099 Δ*rel*Δ*relS*Δ*relH*. According to expectations, considerable differences were found under dark conditions. Strains without *crtR* deletion were almost white, while deletion of the MarR-type repressor caused a distinct yellow coloration (Supplementary Figure S2). However, quantification of the decaprenoxanthin concentration normalized to biomass, generated by growth on solid media, also revealed significant (p)ppGpp-associated differences. Over 70% more of the carotenoid was formed in the parental strain missing *crtR* compared to CR099 Δ*rel*Δ*relS*Δ*relH*Δ*crtR* (Supplementary Figure S2). As expected, the influence of light in both the parental and (p)ppGpp^0^ strains caused a significant increase in decaprenoxanthin production, whereas the altered conditions had no effect on strains with deletion of *crtR*. However, both with and without deletion of the regulator gene *crtR*, normalized carotenoid titers in strain CR099 were over 40% higher than in the (p)ppGpp^0^ strain even under these conditions.

The amino acid quantification in cultivation supernatants revealed significant differences between the parental strain and the (p)ppGpp^0^ mutant as well as within the replicates of the (p)ppGpp-devoid strain. In addition, the latter showed large variations between light and dark conditions. Without exposure to light, significantly elevated L-alanine and L-valine concentrations were measured for two replicates of the (p)ppGpp^0^ mutant. Compared to the values determined under dark conditions, the parental strain produced slightly more glutamate under the influence of light, averaging 185 mg/L. In contrast, the three cultivation approaches of the (p)ppGpp^0^ strain showed elevated supernatant concentrations between 320 and 340 mg/L. While again no valine or alanine was found in the supernatant of the parental strain cultures, considerable amounts of these two amino acids were detected for all CR099 Δ*rel*Δ*relS*Δ*relH* replicates analyzed. The two cultures with the lowest final optical densities each reached supernatant concentrations of more than 1.25 g/L alanine and 2.4 or 3 g/L valine after 44 h.

Since a distinct yellow coloration of individual supernatants had been determined in the course of the previous analyses, an HPLC-MS analysis for the quantification of water-soluble vitamins was performed. The parental strain secreted slightly more riboflavin under dark conditions averaging 1.1 mg/L. According to the general trend, riboflavin production in cultivations of the (p)ppGpp^0^ mutant was very different. In addition to a replicate with significantly lower production, a strongly increased riboflavin concentration of 2.87 mg/L was determined for replicate 2, in agreement with the observed yellow coloration. Furthermore, 0.54 mg/L Ca-pantothenate was detected for this replicate. Under the influence of light more riboflavin and Ca-pantothenate was produced from all (p)ppGpp^0^ replicates, compared to the parental strain.

Furthermore, a GC-MS based metabolome analysis of the supernatant was performed in order to find explanations for the enhanced secretion of amino acids or B-vitamins. In the supernatant of CR099 Δ*rel*Δ*relS*Δ*relH* cultures, increased L-homoserine titers were identified in the dark. Under light conditions there was an increase in further metabolites. Compared to the parental strain, the concentrations of pyruvate and malate were 10 times higher on average. The homoserine concentrations were increased between 22 and 57 fold. The strongest changes were observed with respect to α-ketoglutarate, which was elevated in the supernatant of the (p)ppGpp^0^ mutant by factors between 112 and 353.

### Differential Transcriptome Analysis Under Total Starvation Conditions Clarifies Global Effects of (p)ppGpp on Central Metabolism Under Conditions of Nutrient Deficiency

To analyze the transcriptional changes in the context of the stress response of *C. glutamicum* and to verify the influence of (p)ppGpp on cellular regulation processes, an RNAseq study was performed. Therefore, both the parental strain CR099 and the ppGpp^0^ strain CR099 Δ*rel*Δ*relS*Δ*relH* were cultured until the early exponential growth phase was reached and subsequently transferred to a total starvation situation.

**FIGURE 6:**
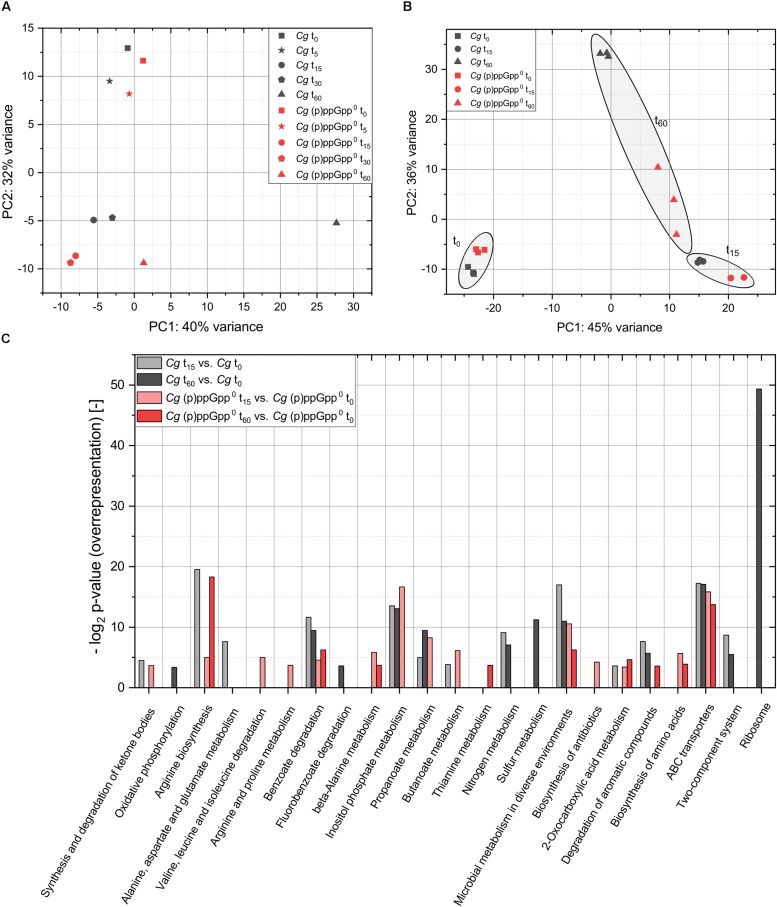
Principal component analysis (PCA) and over-representation analysis (ORA) of the RNAseq data from total starvation analysis of CR099 and CR099 Δ*rel*Δ*relS*Δ*relH*. **(A)** PCA of pooled biological triplicates for all time points sampled. **(B)** PCA of three biological replicates from three sampling points, prior to stress exposure and after 15 or 60 min of total starvation. Related data points of t_0_, t_15_, and t_60_ are highlighted. Read counts were determined using *ReadXplorer* 2 (Hilker et al., 2016). After data normalization using *DESeq2*, PCA analysis was performed with R (Love et al., 2014; R Core Team, 2018). **(C)** Over-representation analysis (ORA) of the differentially regulated genes with respect to their assignment to KEGG-pathways using *DAVID* 6.7 (Huang et al., 2009a, b).

In order to identify the most relevant time points for the analysis of (p)ppGpp-dependent transcriptional effects, an initial RNAseq analysis was performed based on pooled biological triplicates and evaluated by performing a PCA. The variance of the transcriptome data compared to the untreated initial state (t_0_) was represented in both the parental strain and the (p)ppGpp-devoid mutant up to the t_30_ sample primarily by the second main component (Figure 6A). Only minor differences were present compared to the initial state after 5 min of stress exposure and the deviations present after 15 min were almost identical to the result of PCA analysis for t_30_. The t_60_ dataset of the parental strain CR099 deviated significantly from the initial state even on the first main component. The corresponding transcriptional changes obviously could not be realized in the (p)ppGpp^0^ strain, since the result of the corresponding dataset differed to a much lesser extent from the time points t_15_ and t_30_. Based on this result, the stress exposure times t_15_ and t_60_ were selected for further analysis in the form of separately analyzed biological triplicates, as these almost fully represent the variance within the data and thus the transcriptional responses.

In order to compare the transcriptional variance between the two strains investigated in the course of the stress response with the deviations within the individual replicates, a further PCA analysis was performed. The datasets before stress induction and 15 min after induction revealed only a small variance between the three biological replicates for both the parental strain and (p)ppGpp^0^ samples (Figure 6B). However, the replicates of strain CR099 Δ*rel*Δ*relS*Δ*relH* showed a higher internal variance with respect to the first main component. As expected, the obtained t_60_ datasets differed significantly from each other. In this context, the second major component illustrated significant differences between the single cultures of CR099 Δ*rel*Δ*relS*Δ*relH*, while the cultivation of CR099 showed very little internal variation.

For fundamental classification of processes regulated within the strains studied in the course of starvation, an ORA of the differentially regulated genes with respect to their assignment to KEGG-pathways was performed using *DAVID* 6.7 (Huang et al., 2009a, b). According to the results already presented, the statistically significant over-representation of differentially regulated genes in numerous pathways corresponded to global regulatory processes (Figure 6C). Besides pathways like benzoate degradation, microbial metabolism in various environments and ABC transporters which showed an over-representation of differentially regulated genes in all strains and stress exposure times, accumulations of regulated genes also occurred depending on the strain as well as the sampling time. Significant over-representation of translation-associated genes within the genes regulated in the parental strain CR099 after 60 min was particularly striking and highly interesting in the context of the stringent response.

**FIGURE 7:**
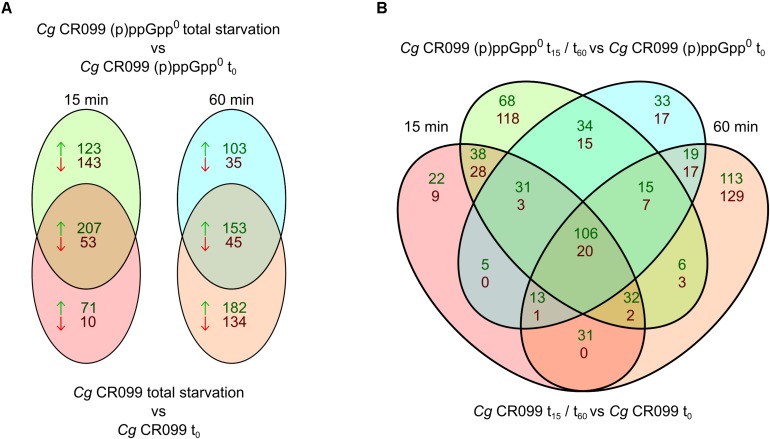
Venn diagrams of differentially regulated genes between parental strain CR099 and (p)ppGpp^0^ mutant CR099 Δ*rel*Δ*relS*Δ*relH* for two stress exposure durations. For both strains, the stress-induced change in transcript levels was determined in relation to the unstressed initial state. **(A)** Venn diagrams of individual stress exposure durations. **(B)** Combined Venn diagram of both strains and stress exposure durations. Individual Venn diagram elements represent the genes differentially up- or downregulated in the respective time points (t15: left; t60: right) and strain backgrounds (CR099: bottom; CR099 Δ*rel*Δ*relS*Δ*relH*: top). The numbers of positively regulated genes are shown in green and the downregulated genes in red. Differentially regulated genes of all subgroups are listed comprehensively with their corresponding log_2_ ratios (*M*-values) in Supplementary Tables S3–S13.

For a more detailed analysis of such relationships, which correspond to the transcriptional regulation depending on the presence of alarmones, the regulated genes were sorted according to their occurrence in the datasets of both strains. The groups determined for both individual time points complied with (p)ppGpp-independent (both strains), (p)ppGpp-dependent (only CR099) or (p)ppGpp^0^-related (CR099 Δ*rel*Δ*relS*Δ*relH*) transcriptional regulation (Figure 7A). After 15 min, significantly more genes were exclusively upregulated (123) or downregulated (143) in the (p)ppGpp^0^ strain than in the parental strain, which comprised 71 genes regulated positively and 10 negatively compared to the unstressed condition. After 60 min of starvation with 335 genes up- and 179 downregulated, enhanced transcriptional changes were present in the parental strain, whereas the (p)ppGpp^0^ mutant showed less differentially regulated genes. The delayed occurrence of an exclusive transcriptional response in the parent strain was further illustrated by the more detailed breakdown of the data, taking into account both strain background and duration of starvation exposure (Figure 7B).

For an overview of the cellular processes influenced by the (p)ppGpp-dependent starvation stress response the differentially regulated genes were considered according to their assignment to functional groups of the Cluster of Orthologous Groups (COGs) scheme (Table 3). A comprehensive list of all analyzed subgroups is shown in Supplementary Table S1. Analysis of genes differentially regulated exclusively in strain CR099 showed distinct differences between the two stress exposure durations analyzed. Genes positively regulated after 15 min of stress exposure were mainly associated with energy production, amino acid metabolism, carbohydrate metabolism and metabolism and transport of inorganic ions. After 60 min, genes of these classes were differentially regulated in both directions compared to the untreated initial state. Compared to the t_15_ sample, nucleotide metabolism, lipid metabolism and transcription were also affected by differential regulation. The translation-associated genes represented a striking group of differentially regulated genes. 46 out of 74 members of the corresponding COG class J were (p)ppGpp-dependent downregulated, while only a few representatives of this group occurred both within (p)ppGpp-independent and the (p)ppGpp^0^-associated regulation.

**TABLE 3:** Analysis of (p)ppGpp-dependent differentially transcribed genes with regard to their affiliation to (p)ppGpp metabolism associated functional groups of the Clusters of Orthologous Groups (COGs) scheme (Tatusov et al., 2000; Meyer et al., 2003), as well as their membership to regulons of various sigma factors and regulators of sulfur and iron metabolism.

**Functional group [COG]**	**15 min**		**60 min**	
	+	−	+	−
	**71**	**10**	**182**	**134**
Energy production [C] (90)	11	0	20	10
Amino acid metabolism [E] (152)	15	2	29	8
Nucleotide metabolism [F] (33)	0	0	2	5
Carbohydrate metabolism [G] (128)	11	1	24	5
Lipid metabolism [I] (36)	1	0	7	0
Translation [J] (74)	0	0	0	**46**
Replication and repair [L] (45)	0	0	0	1
Inorganic ion metabolism [P] (126)	8	0	22	8
σ^B^ regulon (121) (Ehira et al., 2008)	6	0	9	1
σ^H^ regulon (122) (Busche et al., 2012)	7	1	9	3
McbR regulon (46) (Rey et al., 2005)	0	1	**36**	0
CysR regulon (16) (Rückert et al., 2008)	0	0	**9**	0
SsuR regulon (9) (Koch et al., 2005)	0	0	**8**	0
DtxR regulon (58) (Brune et al., 2006)	1	1	8	1

*Regulated fractions comprising over 50% of the respective COG class representatives are shown in bold. A comprehensive list of (p)ppGpp-independent, -dependent and (p)ppGpp^0^-associated differentially transcribed genes as well as all COG, sigma factor and regulator assignments is shown in Supplementary Table S1.*

As expected, the (p)ppGpp-independent response to total starvation covered a broad spectrum of cellular processes. In particular, elementary metabolic processes of the COG classes C, E, G, and P as well as transcription-associated genes were differentially regulated compared to the initial state. Transcriptional changes within strain CR099 Δ*rel*Δ*relS*Δ*relH* were also distributed across a broad spectrum of functional groups. Considering genes associated with central metabolism, a specific regulation of amino acid synthesis pathways was noticeable. This comprises a stronger upregulation of the gene *alaT* encoding pyruvate-alanine aminotransferase, an increased transcription of *ilv* genes involved in the synthesis of branched-chain amino acids, some *aro* genes involved in the synthesis of aromatic amino acids as well as the *trp* operon responsible for tryptophan synthesis. Since transcriptional attenuation has been identified for parts of the *ilv*, *aro*, and *trp* genes (Mentz et al., 2013; Neshat et al., 2014), all known representatives of this type of regulation were examined more closely. It turned out that all genes under ribosome-mediated attenuation control, as well as the respective leader peptides were affected. The analysis of the temporal pattern using the RNAseq datasets for pooled biological replicates showed a maximum of upregulation of *ilv*, *aro*, and *trp* genes in the (p)ppGpp^0^ strain for 15 and 30 min of exposure to total starvation (Supplementary Figure S3). *leuA* and its leader peptide *leuL* were solely upregulated after 5 min of stress exposure. The basic tendency of a total starvation-associated upregulation of these amino acid biosynthesis operons was also observed in the parental strain, but the transcriptional changes were considerably weaker and did not exceed the significance criteria used.

Differentially regulated genes were also investigated with respect to known sigma factor regulons and other transcriptional regulatory networks. σ^B^-dependently transcribed genes (Ehira et al., 2008) were partially regulated (p)ppGpp-independent but were also consistent with those downregulated after 15 min in strain CR099 Δ*rel*Δr*elS*Δ*relH* or upregulated (p)ppGpp-dependently after 60 min of stress exposure. Representatives of the σ^H^ regulon were mainly upregulated in the (p)ppGpp-devoid deletion mutant as well as (p)ppGpp-independently. Numerous genes differentially regulated in the parental strain CR099 were also influenced by the transcriptional regulators of sulfur metabolism: McbR, repressor and master regulator, CysR, the activator of assimilatory sulfate reduction and SsuR, the activator of alternative sulfur metabolism, as well as the iron metabolism regulator DtxR. 36 genes upregulated (p)ppGpp-dependently after 60 min were regulated by McbR, while 10 genes of the McbR-regulon were downregulated in the (p)ppGpp-devoid strain after 15 min. Similar tendencies could also be found for the CysR and DtxR regulated genes, whereby many representatives of the DtxR regulon were also (p)ppGpp-independently downregulated.

Analysis of the most strongly upregulated genes in CR099 after 60 min of starvation underlined a correlation between transcription factor mediated regulation and the presence of (p)ppGpp. The 30 most strongly positively regulated genes mainly encode components of sulfur, propionic acid, or inositol metabolism as well as putative transporters (Table 4). A considerable proportion of these genes is regulated by the regulators McbR, CysR, SsuR, DtxR, and PrpR. In the (p)ppGpp^0^ strain the corresponding genes were not or only slightly positively regulated. In contrast, genes not addressed by regulators were (p)ppGpp-independent upregulated, except for genes *cg3399* and *cg0018*. For the *M*-values of seven genes of strain CR099 Δ*rel*Δr*elS*Δ*relH* no *p*_adj_-value could be calculated. The *DESeq2* program used for data evaluation thus reacted to a too high deviation of the read counts within the biological replicates. A comparison of the raw read counts normalized to RPKM values showed that all three biological replicates were affected by deviations after stress exposure of 60 min (Supplementary Table S2).

**TABLE 4:** Top-scorer analysis of genes characterized by increased transcript levels in the parental strain CR099 after 60 min of stress exposure compared to the unstressed state, as well as their affiliation to the regulons of different regulators of sulfur, iron, and propionate metabolism (A: activator; R: repressor).

**Gene**		**CR099**		**CR099 (p)pGpp^0^**		**Regulation**				
			
**Locus tag**	**Gene name**	***M***	***p*_adj_**	***M***	***p*_adj_**	**McbR**	**CysR**	**SsuR**	**DtxR**	**PrpR**
*cg3119*	*fpr2*	9.18	2.1E−304	0.77	^∗^	R	A		R	
*cg3113*	*cysY*	8.66	0	0.96	0.20765	R	A		R	
*cg3118*	*cysI*	8.36	6.4E−128	0.26	^∗^	R	A		R	
*cg3112*	*cysZ*	8.30	0	1.01	0.168504	R	A		R	
*cg3117*	*cysX*	8.23	5.45E−74	0.04	^∗^	R	A		R	
*cg3115*	*cysD*	8.00	9.74E−72	0.52	0.526895	R	A		R	
*cg3116*	*cysH*	7.96	9.12E−64	0.24	^∗^	R	A		R	
*cg3114*	*cysN*	7.93	6.7E−109	-1.1	0.145052	R	A		R	
*cg3399*		6.34	0	0.6	0.435792					
*cg3391*	*idhA1*	5.90	7.57E−91	4.84	1.22E−24					
*cg0762*	*prpC2*	5.76	0	1.58	^∗^					A
*cg0760*	*prpB2*	5.66	1.3E−118	1.65	0.02341					A
*cg0012*	*ssuR*	5.57	6.7E−296	0.19	0.81453	R	A			
*cg3390*		5.53	1.08E−97	4.95	4.6E−28					
*cg3280*		5.49	0	4.67	0					
*cg0464*		5.31	0	5.63	0					
*cg3392*	*idhA2*	5.31	2.2E−107	4.68	9.48E−25					
*cg3402*		5.29	0	5.16	0					
*cg1376*	*ssuD1*	5.17	1.97E−37	1.24	0.003146	R		A		
*cg0759*	*prpD2*	5.15	0	1.84	0.010373					A
*cg3279*		5.14	0	4.66	9.3E−196					
*cg1377*	*ssuC*	5.14	7.08E−95	0.22	0.753393	R		A		
*cg4028*		5.12	2.99E−78	2.2	0.00013					
*cg3282*		5.11	3.37E−73	4.72	0					
*cg3281*		5.04	2.1E−119	4.42	0					
*cg2678*		4.99	0	0.43	0.561184	R				
*cg1379*	*ssuB*	4.88	1.98E−71	0.58	0.371168	R		A		
*cg3411*		4.83	0	4.6	1.5E−292					
*cg0018*		4.76	9.61E−40	0.89	0.009047					
*cg0755*	*metY*	4.71	0	0.27	0.745413	R				

*Beside the *M*- and *p*_*adj*_-values of strain *C. glutamicum* CR099 the respective values of the (p)ppGpp^0^ strain are listed. ^∗^The transcript levels of certain genes showed considerable differences for CR099 Δ*rel*Δ*relS*Δ*relH* replicates after 60 min of stress exposure. Thus, the measured values were not considered as replicates by *DESeq2* and no probability value was calculated. The read counts of the individual replicates normalized to reads per kilo base per million mapped reads (RPKM-values) are shown in Supplementary Table S2.*

### Promotor Sequence Analysis of Genes Exclusively Regulated in Strain CR099 Reveals DNA Signatures Associated With ppGpp-Dependent Transcriptional Regulation

**FIGURE 8:**
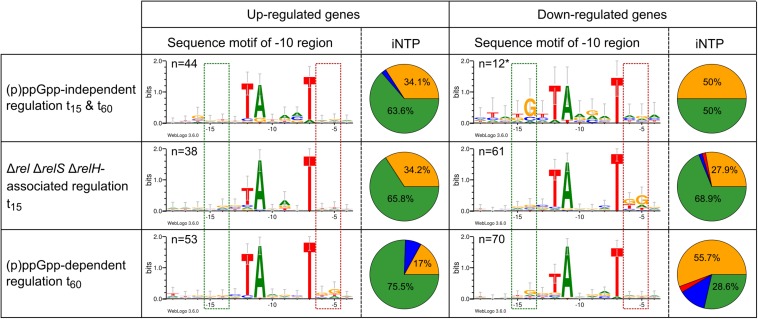
Initial NTP (iNTP) distribution and sequence logos of the −10 motif within selected differentially regulated groups of total starvation analysis between wild type strain CR099 and (p)ppGpp-devoid mutant Δ*rel*Δ*relS*Δ*relH*. The percentage composition of the nucleic acid at position +1 (iNTP) is shown in the form of a pie chart: green: ATP; red: UTP; blue: CTP; yellow: GTP. The −10 motif was identified using *Improbizer* (Ao et al., 2004) and the corresponding sequence was displayed with Weblogo3 (Crooks et al., 2004) as sequence logo from position −18 to −4. The stack height corresponds to the sequence conservation, measured in bits, and the height of each letter corresponds to the relative frequency of the corresponding nucleic acid at that position. The green box corresponds to the localization of the extended −10 motif (Barne et al., 1997) and the red box illustrates to the position of the −10 motif extension, which has been identified for σ^B^ dependent promoters (Ehira et al., 2008) ^∗^Since no *Improbizer*-motif of the −10 region was found by *Improbizer* due to the small number of samples, the predicted −10 motif localizations of the original data were used (Pfeifer-Sancar et al., 2013; Albersmeier et al., 2017).

Since the initial NTP (iNTP) is significantly involved in the (p)ppGpp-associated transcription regulation in Firmicutes such as *B. subtilis* (Krásný and Gourse, 2004; Krásný et al., 2008; Tojo et al., 2008), the transcription start points (TSS) of the gene classes determined by differential transcriptome analysis were examined. In addition, the promoter regions 50 bp upstream of the TSS were analyzed in order to investigate the possible direct DNA signature associated influence of the alarmone species on transcriptional changes in the context of starvation exposure. Sequence information used for this purpose originate from native 5′ end transcriptome datasets which were determined for a pooled approach from different cultivation conditions including different stress factors (Pfeifer-Sancar et al., 2013; Albersmeier et al., 2017). Thus, the data potentially cover a wide range of promoters.

(p)ppGpp-independently regulated genes and differentially regulated genes associated with the genotype Δ*rel*Δ*relS*Δ*relH* exhibited a +1 composition, which is very close to the average occurrence at this position found in *C. glutamicum* of 63.1% A; 33% G, 2.6% C, and 1.3% T (1,657 promoters) (Figure 8). Genes exclusively upregulated in the parental strain after 60 min possessed a reduced G content of 17% and more ATP (75.5%) as their iNTP. Exclusively downregulated genes in this strain, on the other hand, exhibited opposite tendencies. The number of genes with GTP as iNTP was significantly increased to 55.7% and the proportion of ATP was reduced to 28.6%. A relatively large proportion of CTP was also apparent. With 12.9% the amount was nearly five times as high as the average occurrence of the corresponding base at this position.

In contrast to the genes regulated (p)ppGpp-dependently after 15 min, for which no abnormalities apart from the classical −10 motif were detected (data not shown), further sequence motifs were identified for the genes regulated exclusively in strain CR099 Δ*rel*Δ*relS*Δ*relH*. The −10 sequence motif found for the downregulated genes after 15 min contained a downstream extension by moderately conserved G at positions −6 and −5, with the latter occurring more frequently. The particularly interesting group of genes exclusively regulated in the parental strain CR099 after 60 min of stress exposure featured opposite tendencies compared to the group described previously. Differentially upregulated genes had a distinct accumulation of G at positions −6 and −5, while negatively regulated genes of this group displayed weak enrichment of G at position −14, also known as extended −10 motif (Albersmeier et al., 2017).

**FIGURE 9:**
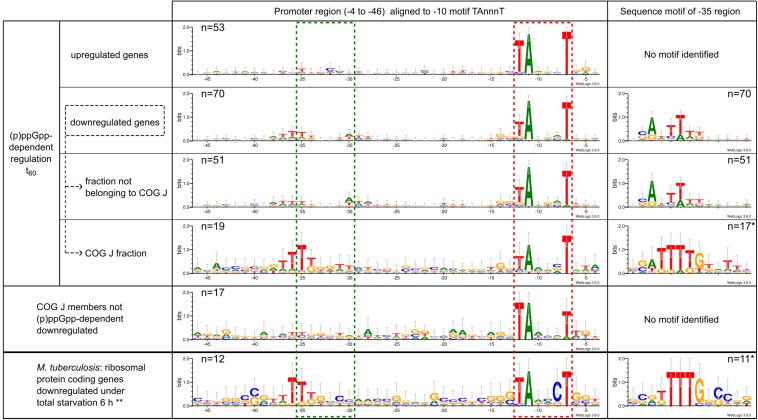
Promoter sequence characteristics of *C. glutamicum* gene showing similar transcriptional regulation in the context of (p)ppGpp-dependent response to starvation stress. The −10 motif was identified using *Improbizer* (Ao et al., 2004) and the sequence aligned with this hexamer was displayed with *Weblogo*3 (Crooks et al., 2004) as sequence logo from position −46 to −4. The stack height corresponds to the sequence conservation, measured in bits, and the height of each letter corresponds to the relative frequency of the corresponding nucleic acid at that position. The location of the −10 region is marked as a red dotted box and the −35 region as a green dotted box. In addition, the *Improbizer* tool was used for the identification of conserved −35 motifs. The respective region is also illustrated as a sequence logo after alignment of the promoter sequences to the identified motif. ^∗^Promotor sequences for which no −35 motif could be identified by *Improbizer* were not taken into account for the presentation of the −35 motif, resulting in a reduced sample size. ^∗∗^Ribosomal proteins showing negative transcriptional regulation in *M. tuberculosis* WT after 6 h of total starvation exposure (Dahl et al., 2003); TSS detection was based on a 5′ enriched RNAseq dataset by Schneefeld et al. (2017).

In the course of the promoter analysis, a significant motif localized in the −35 region was found for the group of (p)ppGpp-dependent downregulated genes after 60 min of starvation exposure (Figure 9). All other groups described in this study had no enriched nucleotides in this position. Based on the promoter sequences aligned at the −10 region, the motif represented a T-triplet at positions −37 to −35. Since the conserved TTG motif of the common −35 motif associated with housekeeping sigma factors is located in the position range −35 to −33, this observation was an interesting result and has been further analyzed. An alignment of the promoter sequence with respect to the specific sequence motif identified at this position using *Improbizer* (Ao et al., 2004) resulted in a CAnTTT motif. Especially COG class J, the subgroup of translation-associated genes revealed a highly significant motif that was examined in more detail. An alignment anchored at the −35 region resulted in a highly conserved TTTTG motif flanked by other moderately conserved bases. In contrast, genes of class COG J that were not differentially regulated during starvation stress showed no apparent sequence motif in the −35 region.

Since a (p)ppGpp-dependent downregulation of ribosomal proteins was also found for *M. tuberculosis* under total starvation condition (Dahl et al., 2003), the corresponding promoters were also analyzed with regard to possible sequence motifs. The promoter regions were identified based on 5′ enriched RNAseq data (Schneefeld et al., 2017). Due to the arrangement of ribosomal proteins in several operons, the evaluated group comprised only 12 promoters. Despite this small sample size, a clear enrichment of T in the −35 region was observed when the sequences were aligned with the highly conserved −10 region (Figure 9). An alignment on the sequence motif of the −35 region resulted in a clear TTTTGnC motif, which shows a high agreement with the sequence motif TTTTG found in *C. glutamicum*.

## Discussion

### Stress Associated Alarmone Production in *C. glutamicum*: In Search of Magic Spot Three

Analysis of the (p)ppGpp-associated gene regulation is conventionally performed by induction of an amino acid deficiency situation using the seryl-tRNA synthetase inhibitor SHX (Tosa and Pizer, 1971a). In the course of this study it was shown that this induction method is not suitable for the actinobacterial model organism *C. glutamicum*. The addition of 1 mg/mL only led to a minor ppGpp level increase (Figure 1). Comparatively low (p)ppGpp concentrations have already been observed in earlier studies of a *C. glutamicum rel* partial deletion mutant in direct comparison with *E. coli* (Wehmeier et al., 1998, 2001). Furthermore, growth of *C. glutamicum* CR099 is only minimally affected by the addition of SHX (Supplementary Figure S1). This observation contradicts the expected molecular mechanism of action, which is based on a sudden termination of translation due to the absence of seryl-tRNA species (Tosa and Pizer, 1971a) and thus results in growth restriction (Tosa and Pizer, 1971b). For example, the addition of a 10-fold smaller amount of 100 μg/ml SHX in the *E. coli* wild type strain MG1655 resulted in a sudden growth arrest (Durfee et al., 2008). The observed difference between both organisms is therefore most likely due to the lack of uptake of SHX or an insufficient specificity of seryl-tRNA synthetase for SHX compared to serine. A similar behavior has already been demonstrated for *Helicobacter pylori* and *M. tuberculosis* (Scoarughi et al., 1999; Primm et al., 2000).

The investigation of alarmone production for different alternative stresses is in basic agreement with the results for *M. tuberculosis* and *Mycobacterium smegmatis*, both also representing important actinobacterial model organisms (Primm et al., 2000; Dahl et al., 2005). Similar to these organisms, (p)ppGpp production can be induced by total starvation, sodium azide and D,L-norvaline. However, the initiation of a total starvation situation leads to a significantly stronger ppGpp production compared to all tested alternative induction methods. Since the expected molecular mechanisms of these stressors would each result in a sudden strong alarmone production, they all seem to indirectly cause an increase in ppGpp concentration by triggering stress based on diverse and so far unknown molecular mechanisms. The exact processes of alarmone production in *C. glutamicum* under total starvation conditions therefore require further analysis, since no information on the molecular functional principle could be generated based on the tested substances. On the basis of the already known association between Rel and the ribosome (Wehmeier et al., 2001), however, a sensing of the deficiency conditions based on the tRNA loading status is likely.

Analysis of different RSH deletion mutants corresponds to earlier studies in which no (p)ppGpp accumulation was found for a *C. glutamicum* mutant with partial deletion of the *rel* gene (Wehmeier et al., 1998). Under the conditions tested, including amoxicillin stress (data not shown), the (p)ppGpp production is directly linked to the presence of this gene. For the SAS enzyme RelS, on the other hand, no alarmone production was detected. Its biological function therefore possibly differs from other monofunctional (p)ppGpp synthetases in which transcriptional activation as well as pyrophosphokinase activity under specific stress conditions has been demonstrated (Nanamiya et al., 2008; Geiger et al., 2014). Likewise, it is possible that so far untested conditions could lead to RelS induction. Since the deletion of *relS* causes a growth phenotype in the exponential growth phase, furthermore, a participation of RelS in the (p)ppGpp basal level is likely.

One of the most interesting results of the nucleotide measurements is the accumulation of an additional substance under stress conditions. The TLC characteristics of the substance, which was found under conditions optimized for hyperphosphorylated nucleotides are comparable to that of GTP and therefore suggests a structural similarity to triphosphorylated nucleotide compounds. pGpp is obvious in this context, since Gaca et al. (2015b) have already demonstrated a biological function in *in vitro* analyses and *C. glutamicum* has the enzymatic repertoire to produce this substance (Ruwe et al., 2017). However, a direct comparison with enzymatically produced GMP 3′-diphosphate showed that the unknown substance is most likely not pGpp. This interpretation corresponds to the occurrence of the corresponding spot under SHX induction (Figure 1), although no change in GTP signal intensity can be observed under these conditions. According to present knowledge, the alarmone species can only be formed by pyrophosphorylation of GMP, GDP or GTP, as well as the conversion into each other by dephosphorylation of the 5′-phosphate residues. Since the cellular GTP:GDP:GMP ratio according to the data determined for *E. coli* is shifted toward GTP under conditions of exponential growth in an approximate ratio of 20:7:1 (Bennett et al., 2009), the molecular basis for the production of a high concentrated guanosine derivative is missing. For this reason, an accumulation of non-G-based nucleotide derivatives such as the substance pppApp recently detected for *Methylobacterium extorquens* is conceivable (Sobala et al., 2019). Based on the comprehensive *in vitro* characterization of the *C. glutamicum* pyrophosphokinases Rel and RelS, which did not indicate the production of pppApp (Ruwe et al., 2017), it is very unlikely that the previously described spot represents this specific substance. However, the accumulation of the unknown spot is linked to the presence of Rel under different stress conditions and therefore associated to the stress response. Unfortunately, identification of the substance is difficult, since the nucleotide concentrations obtained for *C. glutamicum* with established methods are not yet sufficient to perform quantitative LC-MS-based measurements (Kästle et al., 2015; Juengert et al., 2017; Varik et al., 2017; Zborníková et al., 2019). Furthermore, the separation of the nucleotide fraction by HILIC or cHILIC column materials used in recently developed approaches is not comparable with the separation behavior of the PEI cellulose F TLC material, which complicates the assignment of the unknown substance in the complex mixture of all cellular metabolites.

### Basal Level Effects of Alarmones in *C. glutamicum*: (p)ppGpp Probably Work as a Fine Tuner of Central Metabolism Processes

Transcriptome analysis in the early exponential growth phase revealed numerous, but mostly rather small differences between the parental strain and the (p)ppGpp^0^ mutant. This result is not in line with previous analyses, in which significantly stronger and fundamentally distinct differences were found for a *C. glutamicum rel* partial deletion mutant (Brockmann-Gretza and Kalinowski, 2006). Besides technical differences in both studies, these deviations might also result from the (p)ppGpp synthetase activity of RelS, whereby previous measurements do not represent the analysis of a (p)ppGpp^0^ strain. The largest transcriptional differences have been found in the context of sulfur metabolism as well as amino acid and carotenoid synthesis, including the *cys*, *glt*, and *crt* operons. The involvement of stress nucleotides in the transcriptional regulation of amino acid synthesis related genes has already been demonstrated for numerous organisms. However, the effects of (p)ppGpp on the *C. glutamicum* transcriptome seem to be less severe since the (p)ppGpp-devoid strain is able to grow in minimal medium (Ruwe et al., 2018).

The pyruvate pool appears to be particularly affected by (p)ppGpp-based transcriptional regulation, as the metabolically unstable (p)ppGpp^0^ phenotype during shaking flask cultivation indicate an accumulation of this key metabolite of central metabolism. The similar *C. glutamicum* phenotype of L-alanine and L-valine overproduction also results from pyruvate accumulation as a consequence of an inactivation of the pyruvate dehydrogenase complex (PDHC), which catalyzes the decarboxylation of pyruvate to acetyl-CoA and CO_2_ (Blombach et al., 2007). The production of α-ketoglutarate is probably related to the strong production of L-valine. This intermediate was found together with L-alanine and L-glycine as a prominent by-products during the construction process of a L-valine producer based on the overexpression of the L-valine biosynthetic pathway (Brik Ternbach et al., 2005).

The increased intensity of the discussed effects under the influence of light indicates that the central metabolism regulation of the (p)ppGpp^0^ mutant is out of balance especially under stress conditions. Both the exposure to light and the generally deregulated metabolism lead to increased formation of reactive oxygen species (ROS) (Gaca et al., 2013), which cause oxidative stress and unbalance the cell homeostasis. Unbalanced behavior has already been observed for a (p)ppGpp-devoid mutant of *Enterococcus faecalis*, which in contrast to the *C. glutamicum* (p)ppGpp^0^ strain is characterized by an upregulation of operons involved in energy metabolism (Gaca et al., 2012). The lack of fine-tuning functionalities in central metabolism by (p)ppGpp, resulting in the deregulation of elementary metabolic processes with the character of a noise driven reaction, combined with the inability to react with the conserved stress response are most likely also responsible for the unstable phenotype of the (p)ppGpp^0^ strain. A regulatory influence of (p)ppGpp on the pyruvate concentration could directly contribute to the optimization of the cellular resource distribution with respect to given conditions and thus to the regulation of growth behavior. The entry of pyruvate into the TCA cycle through the conversion to acetyl-CoA has already been directly associated with the regulation of growth rate for *C. glutamicum* in this context (Buchholz et al., 2013; Ma et al., 2018).

An increased production of vitamins also observed for individual (p)ppGpp^0^ mutant cultures may be due to aberrant activity or activation of sigma factors in this strain, since σ^H^ overexpression leads to a similar phenotype as well (Taniguchi and Wendisch, 2015). In the early exponential growth phase, however, there is no differential regulation for *sigH* or the *rib*-operon genes involved in riboflavin synthesis. In addition, both positive and negative deviations of the individual CR099 Δ*rel*Δ*relS*Δ*relH* replicates compared to the riboflavin concentrations determined for the parental strain under dark conditions suggest a general influence of the missing (p)ppGpp basal level as unlikely. Rather it can be assumed that the absence of (p)ppGpp may lead to metabolic shifts which indirectly cause activation or reduction of riboflavin synthesis. In this context, a mechanistic involvement of σ^H^ is imaginable even without transcriptional regulation, as both the cognate anti-sigma factor RshA (Busche et al., 2012) and the sigma factor competition at the RNA polymerase could be affected by (p)ppGpp in favor or disfavor of σ^H^. Appropriate functional interrelationships have already been demonstrated for alternative sigma factors of *E. coli* (Jishage et al., 2002; Traxler et al., 2008; Girard et al., 2017). Further evidence for a relationship between (p)ppGpp and sigma factors is provided by deviations in decaprenoxanthin concentrations, also observed for *sigA* overexpression (Taniguchi et al., 2017). The reduction of decaprenoxanthin concentrations is not dependent on the presence of the local regulator CrtR, which in combination with the RNAseq results point to a global influence of (p)ppGpp.

### (p)ppGpp-Dependent Transcriptional Regulation in *C. glutamicum*: Evidence of a Complex Interplay Involving Sigma Factors and Transcriptional Regulators

The transcriptional analysis under total starvation conditions revealed an increased downregulation of the PDHC in the (p)ppGpp-devoid strain after 15 min compared to the parental strain. Although the determined log_2_ fold change for the corresponding gene *aceE* of −1.2 (ratio of 0.435 fold) is only moderately deviating from the value of the parental strain of −0.8 (0.574 fold), the result basically corresponds to the apparent pyruvate accumulation in the mutant strain, which was observed in the course of cultivation under balanced growth conditions. An enhanced negative regulation of this gene and a general connection between pyruvate metabolism and isoleucine starvation response was also found for a (p)ppGpp^0^ *E. coli* strain (Traxler et al., 2008). In line with the *C. glutamicum* (p)ppGpp^0^ mutant, this strain has problems physiologically restructuring its metabolism after isoleucine deficiency. The amino acid metabolism is also deregulated, which however, results in glycine production and glutamate depletion. In addition, the (p)ppGpp-devoid *E. coli* strain showed an imbalance between glutamate and α-ketoglutarate (Traxler et al., 2008). Aberrant α-ketoglutarate values were also found in the (p)ppGpp-devoid *C. glutamicum* mutant under light stress conditions. Since an increased positive regulation of the *ilv* and *aro* genes as well as of *alaT* was determined under total starvation conditions, the increased valine and alanine production is probably also transcriptionally conditioned, in addition to the pyruvate concentration-based effects. Furthermore, the differential regulation of all genes regulated by leader peptide-based transcriptional attenuation, indicates a coupling between (p)ppGpp and translation. Since uncharged tRNA species lead to stalling of the ribosome during the synthesis of the leader peptides and thus to the formation of an anti-terminator complex (Naville and Gautheret, 2010), the transcription of these genes reflects the loading status of the tRNA. The absence of alarmones thus apparently facilitates the accumulation of deacylated tRNAs, as an enhanced up-regulation was observed for the (p)ppGpp^0^ mutant. The production of alarmones, which is caused by the accumulation of unloaded tRNAs according to previous models, seems to quickly trigger a dosed counter-reaction in the parental strain. Since a shift of the tRNA loading status is thus largely avoided, (p)ppGpp seems to contribute to maintaining the cell in a homeostatic state, even with regard to translation.

In addition to the already discussed relationship between the presence of (p)ppGpp and the activity of σ^H^, differential transcript levels were also found for representatives of the σ^B^ regulon in the *C. glutamicum* strains studied. This non-essential primary like sigma factor is mainly present in the transition and early stationary phase (Larisch et al., 2007) but also participates in various stress responses (Ehira et al., 2008; Dostálová et al., 2017). Although the *sigB* gene shows no significant transcriptional difference between parental strain and (p)ppGpp^0^ mutant, genes regulated by σ^B^ under oxygen deprivation (Ehira et al., 2008) are downregulated in the mutant after 15 min and upregulated in the wild type after 60 min of stress exposure. Indications for this relationship can also be found in the promoter motifs analyzed for these groups, each of which shows a slight accumulation of G at position −5 (Figure 8). This motif has been identified as a characteristic feature of σ^B^ regulated genes (Ehira et al., 2008). The phased occurrence could indicate that (p)ppGpp is involved in switching between housekeeping sigma factor σ^A^ and the more stress-associated primary like sigma factor σ^B^. Evaluation of the transcriptional analysis with regard to known regulatory networks also revealed a relationship between (p)ppGpp and the sulfur reduction chain regulated by the McbR, CysR, and SsuR, respectively (Rückert and Kalinowski, 2008) as well as the iron metabolism associated with DtxR (Wennerhold and Bott, 2006). The almost complete transcriptional activation of the sulfur metabolism genes after 60 min of stress exposure is particularly striking as the sulfate concentration of 1 mM present in the starvation solution represents no sulfur deficiency conditions for *C. glutamicum* (Rückert and Kalinowski, 2008). Based on the transcriptome data obtained, the transcriptional activation of *cysR* and *ssuR* by the master regulator McbR only results in the co-activation of the CysR regulon (Rückert et al., 2008), whereas no further activation is recognizable for the genes controlled by SsuR (Koch et al., 2005). A possible connection between this additional level of regulation and (p)ppGpp becomes clear from the different transcript levels of individual genes within the biological replicates of the (p)ppGpp^0^ mutant (Supplementary Table S2). Most of the affected genes are under the control of regulators such as CysR and PrpR, which are influenced by the central metabolic intermediates O-acetyl-L-serine or O-acetyl-L-homoserine (Rückert et al., 2008), and 2-methylcitrate (Plassmeier et al., 2012), respectively. The unstable metabolism of the (p)ppGpp-devoid strain is therefore most likely directly related to regulator-based fluctuations of the transcriptome by aberrant levels of respective metabolites. The involvement of cofactors derived from the central metabolism was also reported for the regulatory effect of the master regulator protein McbR. The identified effectors of McbR, S-adenosyl methionine (Suda et al., 2008) and S-adenosyl homocysteine (Rey et al., 2005) reflect the methylation activity of the cell through their direct participation in transmethylation reactions. It is an attractive hypothesis that McbR actually measures the relationship between both molecules and uses this signal as an indicator of growth. A possible interaction of both systems, which however, needs to be investigated in more detail by further analyses, would extend the already very strict regulation of macroelement homeostasis by a further regulatory level and directly link sulfur metabolism with the stress response. Since the McbR regulon is also responsible for the metabolism of toxic sulfur metabolism intermediates which may accumulate under different stress scenarios, this might be a fundamental feature of the stress response in *C. glutamicum*.

In line with *C. glutamicum*, a (p)ppGpp-dependent downregulation of the cellular translational apparatus was found for *M. tuberculosis* under starvation conditions (Dahl et al., 2003), which represents a known hallmark of the stringent response (Potrykus and Cashel, 2008). In analogy to the *B. subtilis* paradigm of (p)ppGpp-associated transcriptional regulation (Krásný et al., 2008), *C. glutamicum* exhibits a significant accumulation of G at +1 position of genes that are differentially (p)ppGpp-dependent regulated after 60 min of stress exposure. This suggests that the GTP level fulfills an important functional role in corynebacterial transcription regulation. The temporal scale illustrates that the stress response induced by starvation is not as fast as the (p)ppGpp production induced by antibiotics or amino acid analogs due to the presence of nutrient pools. Contrary to expectations no sequence motif was found in the discriminator area between −10 motif and +1 position (Haugen et al., 2006). The slightly enriched G at position −5 is more likely associated to the σ^B^ promoter motif (Ehira et al., 2008). Interestingly, a thymine at position −4 was postulated to be essential for iNTPs and (p)ppGpp mediated transcriptional response in *M. tuberculosis* (Tare et al., 2013). However, this study is based on the analysis of merely two promoters (P_rrnPCL1_ and P_gyrB1_) and our larger survey of (p)ppGpp-dependent differentially transcribed ribosomal proteins from *M. tuberculosis* (Dahl et al., 2003) did not reveal any enrichment of a discriminator motif in this context (Figure 9). In contrast to all previous investigations of (p)ppGpp-associated DNA sequence motifs, which exclusively revealed features in the discriminator region, an enrichment of the motif TTTG was found in the −35 region of (p)ppGpp-dependent down-regulated genes of *C. glutamicum*. A particularly significant enrichment of this motif is present for (p)ppGpp-dependent regulated genes of ribosomal proteins from both *C. glutamicum* and *M. tuberculosis*. This sequence motif is also conserved in further species of Corynebacteria and Mycobacteria and can also be found for other Actinobacteria of the quite distant genera *Actinoplanes* and *Streptomyces* (Supplementary Figures S4–S6). In the course of a mutational analysis of the *E. coli rrnB* P1 promoter, Gaal et al. (1989) found a significant reduction in promoter activity for a TTTTG sequence motif at this position compared to the original motif TCTTG. The promoter region had only 5% of its original activity after replacing a single base at position −37 (C-37T), possibly due to a DNA curvature caused by the resulting T stretch. However, the low promoter activity contradicts the strong transcription of ribosomal proteins and rather suggests that the destabilizing effect of the sequence is part of an intrinsic promoter instability of actinobacterial ribosomal protein genes. Such promoter characteristic, which is known for mycobacterial *rrn* promoters (Tare et al., 2012) could facilitate a +1 position-based downregulation of stringently controlled genes at low GTP levels. Due to the localization of the found motif in the −35 region, which is known to interact with the σ_4_ subunit of σ^70^ sigma factors (Campbell et al., 2002), a sequence-specific influence on promoter recognition by the RNAP is also conceivable. Since the motif is also found in *M. tuberculosis* and a direct effect of (p)ppGpp on RNAP has been demonstrated for this closely related organism (Tare et al., 2013), such a functional relationship is also conceivable for *C. glutamicum* and possibly for Actinobacteria in general.

In order to clarify the hypotheses on the interaction of (p)ppGpp, RNAP, sigma factors, other transcription regulators and iNTP concentration, further investigations are necessary. This could be accomplished, for example, by *in vitro* transcription based methods or mutational analysis using an appropriate reporter system. In this context, a possible association with the RNAP-binding proteins CarD and RbpA should also be analyzed, since both proteins are involved in the transcriptional regulation during various stress responses (Flentie et al., 2016). At least CarD is a functional homolog to the regulatory protein DksA (Stallings et al., 2009), which is involved in the association between RNAP and (p)ppGpp in *E. coli* (Ross et al., 2016). In addition, CarD is necessary for the effects of (p)ppGpp in *M. tuberculosis* and *M. smegmatis* within the context of stringent response (Stallings et al., 2009). It is therefore probable that these two transcription factors or so far unknown functional components are involved in the interconnection between (p)ppGpp concentration and transcriptional regulation in *C. glutamicum*. It is also conceivable that the current paradigm of a non-existent interaction between (p)ppGpp and RNAP in gram-positive bacteria, especially in the group of Actinobacteria, is based on a lack of knowledge of the molecular mechanisms involved. The analysis of the interaction, for example by *in vitro* transcription analysis, might require the corresponding factors. In summary, the results of this study illustrate the need for a deeper understanding of (p)ppGpp mechanisms of action in order to be able to analyze the (p)ppGpp-dependent transcriptional regulation more closely by an optimized experimental design.

## Data Availability Statement

The datasets generated for this study can be found in the EMBL-EBI ArrayExpress database under accession number E-MTAB-8070 (www.ebi.ac.uk/arrayexpress).

## Author Contributions

MR, MP, and JK designed, analyzed, and interpreted the performed experiments. MP and JK supervised the research. MR performed the wet lab experiments and wrote the manuscript. BM carried out the vitamin measurements. MR and TB performed the transcriptome analysis using RNAseq as well as whole genome sequencing. MP and JK revised the manuscript. All authors read and approved the final version of the manuscript.

## Conflict of Interest

BM was employed by company Biofidus AG. The remaining authors declare that the research was conducted in the absence of any commercial or financial relationships that could be construed as a potential conflict of interest.
