# Supplementary material for: Physiology and Transcriptional Analysis of (p)ppGpp-Related Regulatory Effects in Corynebacterium glutamicum
Source: Front Microbiol. 2019 Nov 28;10:2769. doi: 10.3389/fmicb.2019.02769 (PMC6892785; doi:10.3389/fmicb.2019.02769)
Supplement: Supplementary file 1 [file Data_Sheet_1.docx]

Supplementary Material

Physiology and transcriptional analysis of (p)ppGpp-related regulatory effects in *Corynebacterium glutamicum*

Matthias Ruwe^1^, Marcus Persicke^1^, Tobias Busche^1^, Benjamin Müller^2^, and Jörn Kalinowski^1^

^1^ Microbial Genomics and Biotechnology, Center for Biotechnology, Bielefeld University, Universitätsstr. 27, 33615 Bielefeld, Germany

^2^ Biofidus AG, Hainteichstr. 78, 33613 Bielefeld, Germany

*** Correspondence:**Jörn Kalinowski
joern@cebitec.uni-bielefeld.de


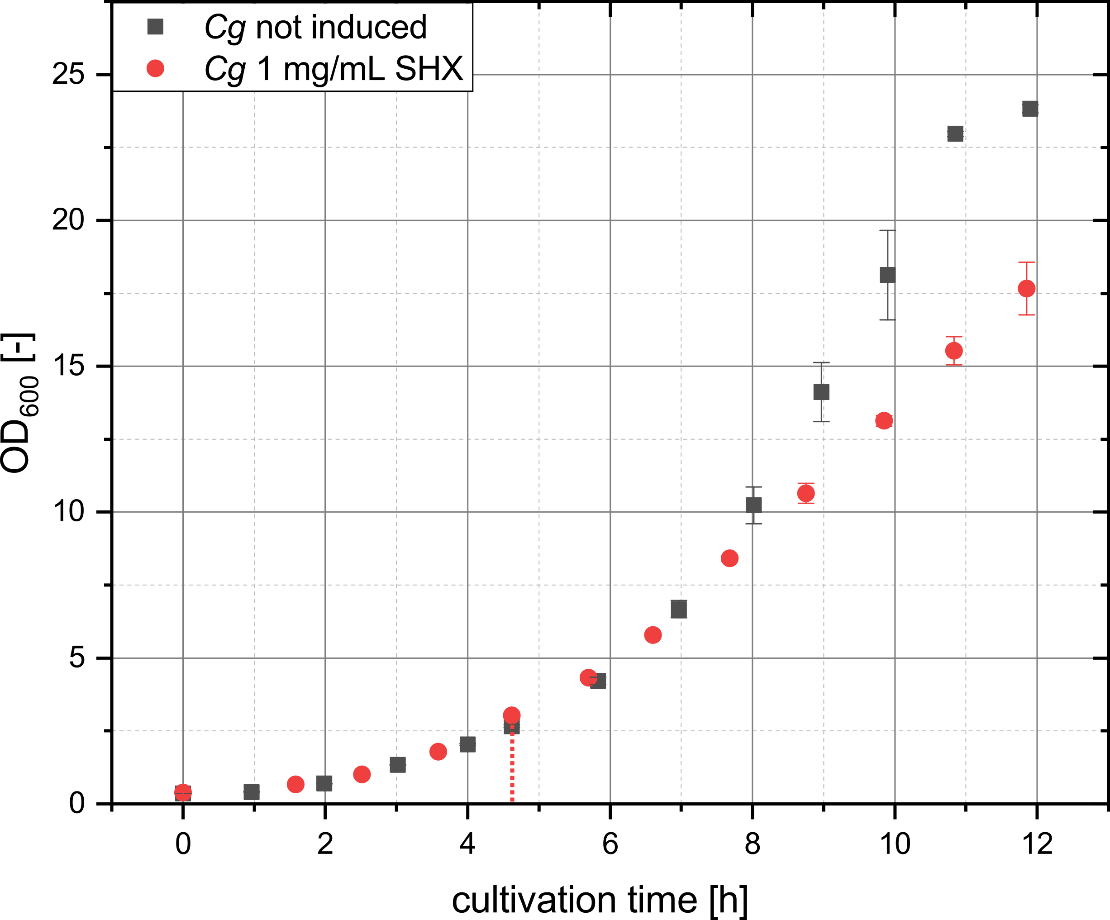


Figure S1: Effect of serine hydroxamate (SHX) addition on growth of *C. glutamicum*. The cultures were grown in CGXII medium (1% glucose) with the additional supplementation of all amino acids (50 mg/L each) from an initial OD_600_ of 0.4 (Keilhauer et al., 1993). After reaching an optical density of 2.5 (red dashed line) serine hydroxamate was added to the cultures with a final concentration of 1 mg/mL. Mean values and standard deviations shown for both treated and untreated cultures were calculated from three biological replicates.


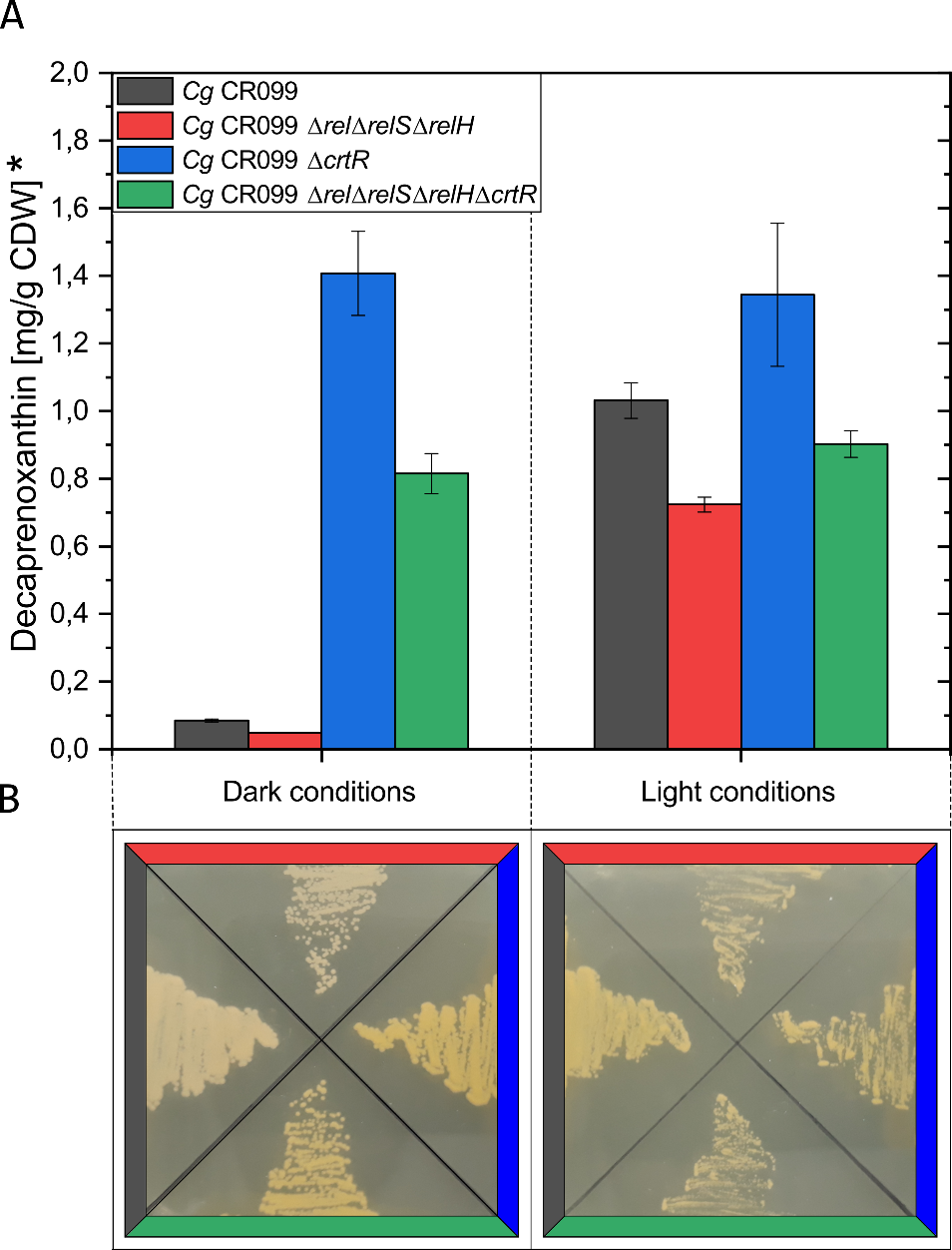


Figure S2: Analysis of light-dependent decaprenoxanthin production in *C. glutamicum* depending on transcription regulator CrtR and the presence of the (p)ppGpp metabolism associated genes *rel*, *relS* and *relH*. The parental strain C. glutamicum CR099 was grown in comparison with the (p)ppGpp free strain CR099 Δ*rel*Δ*relS*Δ*relH*, and *crtR* deletion mutants in both strain backgrounds, on CASO-bouillon solid medium plates under light and dark conditions for 48 hours. A: Decaprenoxanthin concentrations, normalized to the cell weight used for extraction. Cell material was resuspended in PBS buffer for cell density determination and the extracted carotenoids were measured by HPLC analysis. * Due to the unavailability of standards, the decaprenoxanthin concentration was calculated as ß-carotene equivalent. B: Graphical representation of cell morphology and coloration of the strains analyzed. The color code of the border corresponds to the bar chart nomenclature.


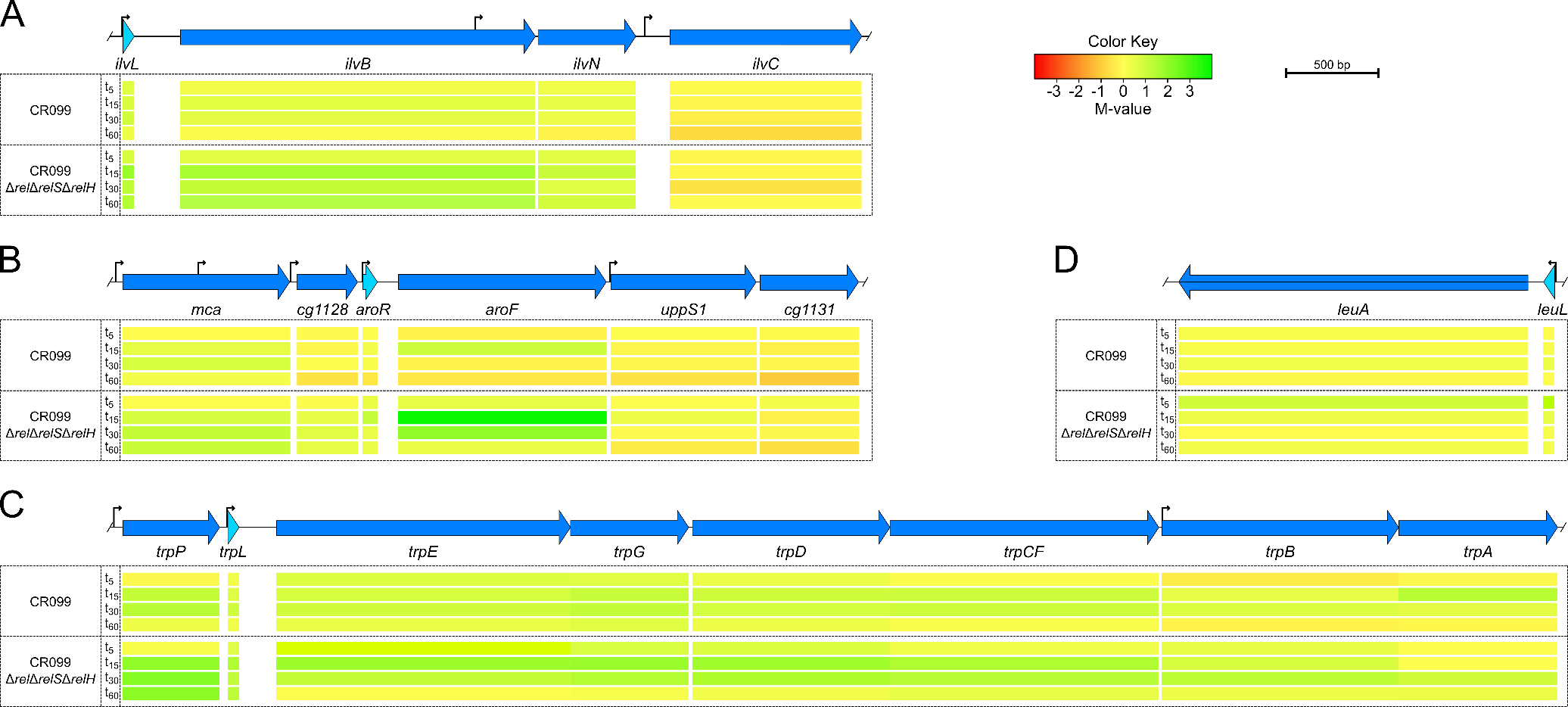


Figure S3: Transcriptional changes of genes regulated by ribosome-mediated transcriptional attenuation in *C. glutamicum* strain CR099 and a derived (p)ppGpp^0^ mutant (CR099 Δ*rel*Δ*relS*Δ*relH*) as a consequence of total starvation. The organization of the transcriptional attenuator targets (A) *ilvBNC* operon, (B) *mca* operon, (C) *trp* operon, (D) *leuA* gene is shown to scale with the known transcriptional start sites indicated as black arrows. In the lower part of the individual figures, the transcriptional changes compared to the unstressed initial state are visualized for the two analyzed strains and 4 stress exposure durations in the form of a heat map. The M-values illustrated were calculated from TPM-values, determined using *ReadXplorer 2* (Hilker et al., 2016).


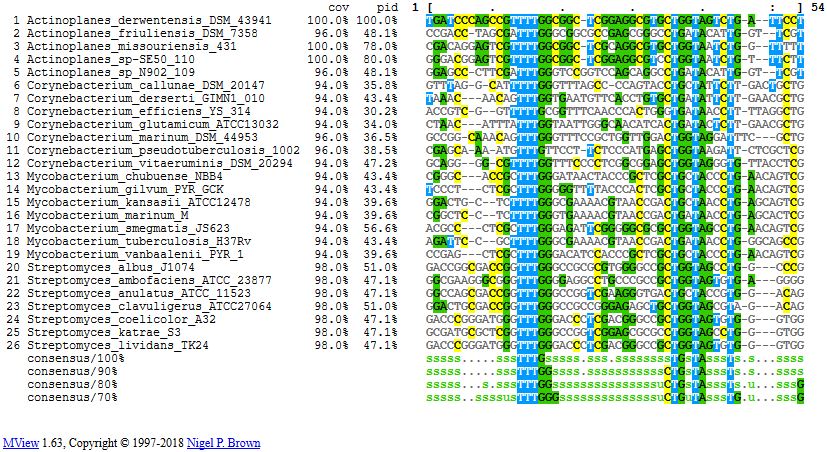


Figure S4: Multiple sequence alignment of 50 bp *rpsT* UTR regions from selected species of actinobacterial genera *Actinoplanes*, *Corynebacterium*, *Mycobacterium* and *Streptomyces*. TSS detection within the investigated genera was performed using the Pan-Genome tool of software platform *EDGAR* (Blom et al., 2016), as well as existing 5`entriched RNAseq data sets of *Actinoplanes sp.* SE50/110 (Wolf et al., 2017), *Corynebacterium glutamicum* ATCC 13032 (Pfeifer-Sancar et al., 2013), *Mycobacterium tuberculosis* H37Rv (Schneefeld et al., 2017) and *Streptomyces albus* J1074 (Myronovskyi et al., 2014). The alignment of all sequences was performed with *T-Coffee* (Notredame et al., 2000) and visualized using *MView* (Brown et al., 1998).


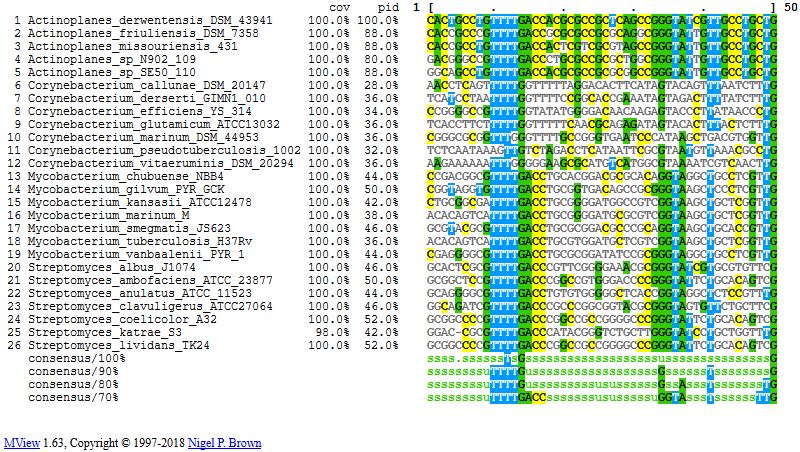


Figure S5: Multiple sequence alignment of 50 bp *rplM* UTR regions from selected species of actinobacterial genera *Actinoplanes*, *Corynebacterium*, *Mycobacterium* and *Streptomyces*. TSS detection within the investigated genera was performed using the Pan-Genome tool of software platform *EDGAR* (Blom et al., 2016), as well as existing 5`entriched RNAseq data sets of *Actinoplanes sp.* SE50/110 (Wolf et al., 2017), *Corynebacterium glutamicum* ATCC 13032 (Pfeifer-Sancar et al., 2013), *Mycobacterium tuberculosis* H37Rv (Schneefeld et al., 2017) and *Streptomyces albus* J1074 (Myronovskyi et al., 2014). The alignment of all sequences was performed with *T-Coffee* (Notredame et al., 2000) and visualized using *MView* (Brown et al., 1998).


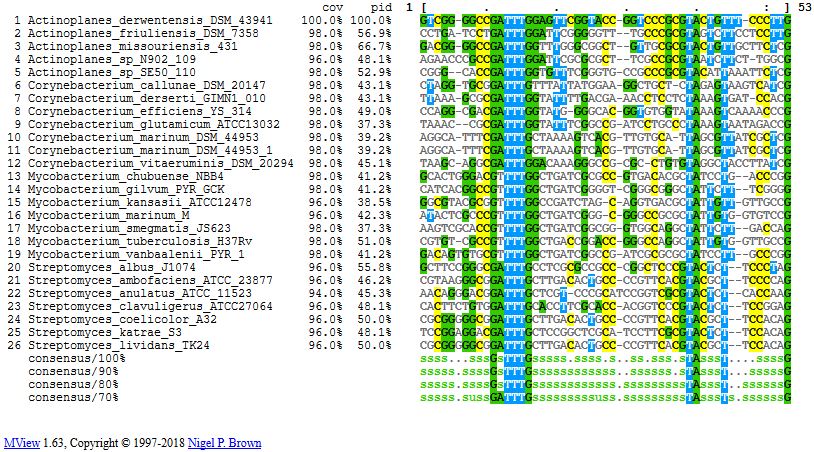


Figure S6: Multiple sequence alignment of 50 bp *rplJ* UTR regions from selected species of actinobacterial genera *Actinoplanes*, *Corynebacterium*, *Mycobacterium* and *Streptomyces*. TSS detection within the investigated genera was performed using the Pan-Genome tool of software platform *EDGAR* (Blom et al., 2016), as well as existing 5`entriched RNAseq data sets of *Actinoplanes sp.* SE50/110 (Wolf et al., 2017), *Corynebacterium glutamicum* ATCC 13032 (Pfeifer-Sancar et al., 2013), *Mycobacterium tuberculosis* H37Rv (Schneefeld et al., 2017) and *Streptomyces albus* J1074 (Myronovskyi et al., 2014). The alignment of all sequences was performed with *T-Coffee* (Notredame et al., 2000) and visualized using *MView* (Brown et al., 1998).

Table S1: Analysis of (p)ppGpp independent, dependent and (p)ppGpp^0^-associated transcriptionally regulated genes with regard to their affiliation to the Clusters of Orthologous Groups (COGs) scheme (Tatusov et al., 2000; Meyer et al., 2003), as well as their membership to regulons of various sigma factors and regulators of sulfur and iron metabolism.

|  | (p)ppGpp independent | | | | (p)ppGpp dependent | | | | (p)ppGpp^0^ associated | | | |
| --- | --- | --- | --- | --- | --- | --- | --- | --- | --- | --- | --- | --- |
|  | 15 min | | 60 min | | 15 min | | 60 min | | 15 min | | 60 min | |
|  | +  207 | -  53 | +  153 | -  45 | +  71 | -  10 | +  182 | -  134 | +  123 | -  143 | +  103 | -  35 |
| C: Energy production and conversion (90) | 15 | 1 | 19 | 0 | 11 | 0 | 20 | 10 | 6 | 9 | 0 | 2 |
| D: Cell cycle control, cell division, chromosome partitioning (3) | 0 | 0 | 0 | 0 | 0 | 0 | 1 | 0 | 1 | 0 | 0 | 0 |
| E: Amino acid transport and metabolism (152) | 39 | 10 | 22 | 5 | 15 | 2 | 29 | 8 | 15 | 15 | 21 | 4 |
| F: Nucleotide transport and metabolism (33) | 5 | 1 | 1 | 0 | 0 | 0 | 2 | 5 | 1 | 2 | 4 | 0 |
| G: Carbohydrate transport and metabolism (128) | 20 | 3 | 12 | 5 | 11 | 1 | 24 | 5 | 14 | 14 | 12 | 2 |
| H: Coenzyme transport and metabolism (59) | 4 | 2 | 1 | 2 | 3 | 2 | 6 | 1 | 7 | 5 | 5 | 2 |
| I: Lipid transport and metabolism (36) | 3 | 1 | 1 | 1 | 1 | 0 | 7 | 0 | 1 | 4 | 1 | 1 |
| J: Translation, ribosomal structure and biogenesis (74) | 3 | 0 | 2 | 0 | 0 | 0 | 0 | 46 | 3 | 1 | 3 | 0 |
| K: Transcription (80) | 12 | 4 | 6 | 2 | 1 | 1 | 9 | 3 | 6 | 12 | 7 | 3 |
| L: Replication, recombination and repair (45) | 1 | 1 | 1 | 2 | 0 | 0 | 0 | 1 | 2 | 6 | 1 | 7 |
| M: Cell wall/membrane/envelope biogenesis (29) | 4 | 4 | 3 | 0 | 1 | 0 | 1 | 2 | 2 | 4 | 2 | 1 |
| N: Cell motility (3) | 1 | 0 | 1 | 0 | 0 | 0 | 0 | 0 | 0 | 1 | 0 | 0 |
| O: Post-translational modification, protein turnover, and chaperone (49) | 6 | 1 | 10 | 0 | 6 | 1 | 4 | 3 | 5 | 6 | 9 | 0 |
| P: Inorganic ion transport and metabolism (126) | 32 | 16 | 23 | 16 | 8 | 0 | 22 | 8 | 9 | 4 | 11 | 2 |
| Q: Secondary metabolites biosynthesis, transport, and catabolism (27) | 6 | 0 | 11 | 0 | 3 | 0 | 4 | 0 | 2 | 4 | 0 | 1 |
| T: Signal transduction mechanisms (27) | 5 | 1 | 4 | 1 | 0 | 0 | 4 | 1 | 3 | 8 | 0 | 1 |
| U: Intracellular trafficking, secretion, and vesicular transport (12) | 0 | 0 | 0 | 0 | 0 | 0 | 0 | 0 | 1 | 1 | 2 | 2 |
| R: General function prediction only (149) | 29 | 4 | 22 | 4 | 8 | 1 | 23 | 7 | 13 | 20 | 13 | 3 |
| S: Function unknown (80) | 14 | 0 | 13 | 1 | 1 | 0 | 9 | 6 | 9 | 9 | 4 | 2 |
| σ^B^ dependent (exponential phase) (121) (Ehira et al., 2008) | 6 | 4 | 7 | 1 | 6 | 0 | 9 | 1 | 4 | 24 | 3 | 1 |
| σ^B^ dependent (Oxygen deprivation) (70) (Ehira et al., 2008) | 6 | 4 | 4 | 2 | 1 | 0 | 4 | 2 | 0 | 9 | 0 | 2 |
| σ^H^ dependent (122) (Busche et al., 2012) | 23 | 1 | 20 | 1 | 7 | 1 | 9 | 3 | 19 | 8 | 25 | 3 |
| σ^C^ dependent (10) (Toyoda and Inui, 2016) | 0 | 0 | 0 | 2 | 0 | 0 | 0 | 2 | 0 | 0 | 0 | 0 |
| McbR regulon (46) (Rey et al., 2005) | 1 | 12 | 4 | 0 | 0 | 1 | 36 | 0 | 2 | 10 | 1 | 3 |
| CysR regulon (16) (Rückert et al., 2008) | 2 | 4 | 4 | 0 | 0 | 0 | 9 | 0 | 2 | 5 | 1 | 0 |
| SsuR regulon (9) (Koch et al., 2005) | 0 | 0 | 1 | 0 | 0 | 0 | 8 | 0 | 0 | 0 | 0 | 0 |
| DtxR regulon (58) (Brune et al., 2006) | 6 | 16 | 5 | 14 | 1 | 1 | 8 | 1 | 0 | 7 | 2 | 4 |

Table S2: Normalized read counts of genes that were not considered as replicates by the *DESeq2* (Love et al., 2014) software after 60 minutes of total starvation stress exposure in the (p)ppGpp^0^-strain CR099 Δ*rel*Δ*relS*Δ*relH* due to deviating values. Read counts of each biological replicate are normalized as reads per kilo base per million mapped reads (RPKM).

| **Locus tag** | **Gene name** | **RPKM t_0_** | | | **RPKM t_60_** | | |
| --- | --- | --- | --- | --- | --- | --- | --- |
|  |  | **Rep 1** | **Rep 2** | **Rep 3** | **Rep 1** | **Rep 2** | **Rep 3** |
| *cg0737* | *metQ* | 866.46 | 848.29 | 728.51 | 322.04 | 2096.2 | 226.57 |
| *cg0762* | *prpC2* | 11.53 | 9.31 | 13.54 | 150.13 | 19.89 | 13.09 |
| *cg1225* | *pcaK* | 19.01 | 17.03 | 18.71 | 20.66 | 29.97 | 183.99 |
| *cg3116* | *cysH* | 36.64 | 55.72 | 36.1 | 2.22 | 76.75 | 1.4 |
| *cg3117* | *cysX* | 68.52 | 94.94 | 56.36 | 5.06 | 204.52 | 3.97 |
| *cg3118* | *cysI* | 49.97 | 62.53 | 45.65 | 5.24 | 207.11 | 4.58 |
| *cg3119* | *fpr2* | 36.66 | 47.88 | 38.52 | 9.04 | 362.43 | 8.84 |

Table S3: (p)ppGpp and time-independent up-regulated genes. M-values of genes exhibiting significantly increased transcript levels in both the parental strain CR099 (WT) and the (p)ppGpp free mutant CR099 Δ*rel*Δ*relS*Δ*relH* (Mut) compared to the respective unstressed initial state after 15 (WT15; Mut15) and 60 minutes (WT60; Mut60) of total starvation.

| Locus tag | Gene name | Product/Function | M-value | | | |
| --- | --- | --- | --- | --- | --- | --- |
|  |  |  | **WT15** | **Mut15** | **WT60** | **Mut60** |
| *cg0077* |  | hypothetical protein | 5.49 | 5.98 | 1.39 | 1.76 |
| *cg0079* |  | putative secreted protein, CotH-like protein | 5.90 | 6.59 | 1.62 | 2.33 |
| *cg0103* | *crnT* | putative drug efflux permease, MFS-type | 2.19 | 2.32 | 1.18 | 4.18 |
| *cg0104* | *codA* | cytosine deaminase | 1.34 | 1.03 | 1.88 | 2.56 |
| *cg0113* | *ureA* | urease gamma subunit | 1.75 | 1.22 | 1.42 | 2.51 |
| *cg0114* | *ureB* | urease beta subunit | 1.69 | 1 | 1.42 | 2.54 |
| *cg0196* | *iolR* | putative transcriptional regulator, GntR-family | 1.55 | 1.9 | 1.07 | 1.18 |
| *cg0229* | *gltB* | glutamate synthase (NADPH), large chain | 4.86 | 3.6 | 3.03 | 4.36 |
| *cg0230* | *gltD* | glutamate synthase (NADPH), small chain | 4.47 | 1.73 | 3.01 | 4.36 |
| *cg0291* |  | putative dioxygenase | 3.13 | 3.76 | 1.07 | 1.23 |
| *cg0317* |  | putative transcriptional regulator, ArsR-family | 1.32 | 1.74 | 1.42 | 1.8 |
| *cg0318* | *arsC1* | putative secondary arsenite transporter, arsenical resistance-3 (ACR3) family | 1.70 | 2.64 | 1.69 | 2.66 |
| *cg0319* | *arsX* | putative arsenate reductase (arsenical pump modifier) | 1.14 | 1.71 | 1.36 | 1.83 |
| *cg0378* |  | putative phage-associated protein | 1.09 | 1.19 | 1.23 | 1.3 |
| *cg0463* |  | hypothetical protein | 4.54 | 5.1 | 3.82 | 5.01 |
| *cg0464* |  | putative Cu2+ transporting P-type ATPase | 6.09 | 5.85 | 5.31 | 5.63 |
| *cg0496* | *mrx3* | mycoredoxin 3 | 1.42 | 2.5 | 1.05 | 2.26 |
| *cg0534* |  | putative integral membrane protein | 1.40 | 1.93 | 1.42 | 1.43 |
| *cg0566* | *gabT* | putative 4-aminobutyrate aminotransferase, AT class II | 1.09 | 1.84 | 1.78 | 1.49 |
| *cg0569* |  | putative Cd2+ transporting P-type ATPase | 3.78 | 4.19 | 3.49 | 4.33 |
| *cg0570* |  | putative dehydrogenase | 2.19 | 2.69 | 1.71 | 2.81 |
| *cg0614* |  | hypothetical protein | 1.53 | 2.05 | 1.31 | 1.82 |
| *cg0616* | *fdhD* | putative formate dehydrogenase, FdhD-family | 1.72 | 2.25 | 1.5 | 2.09 |
| *cg0617* |  | hypothetical protein | 1.67 | 2.44 | 1.5 | 2.21 |
| *cg0727* |  | putative nucleoside-diphosphate-sugar epimerase | 1.24 | 1.03 | 1.5 | 1.19 |
| *cg0923* |  | putative membrane protein | 2.16 | 1.69 | 1.98 | 1.59 |
| *cg0931* |  | putative aminotransferase, AT class I | 1.39 | 1.46 | 1.35 | 1.27 |
| *cg0952* |  | putative integral membrane protein | 1.41 | 1.3 | 1.88 | 1.38 |
| *cg0953* | *mctC* | secondary acetate/propionate/pyruvate transporter | 1.35 | 1.35 | 1.82 | 1.24 |
| *cg0998* |  | trypsin-like serine protease | 3.39 | 3.81 | 1.42 | 1.66 |
| *cg1061* | *urtA* | ABC-type putative branched-chain amino acid transporter, substrate-binding lipoprotein | 2.40 | 2.66 | 1.98 | 4.24 |
| *cg1062* | *urtB* | ABC-type putative branched-chain amino acid transporter, permease subunit | 1.18 | 1.55 | 1.09 | 3.24 |
| *cg1064* |  | ABC-type putative branched-chain amino acid transporter, permease subunit | 1.38 | 2.26 | 1.23 | 4.33 |
| *cg1065* | *urtD* | ABC-type putative branched-chain amino acid transporter, ATPase subunit | 1.77 | 1.82 | 1.8 | 3.93 |
| *cg1066* | *urtE* | ABC-type putative branched-chain amino acid transporter, ATPase subunit | 1.69 | 1.53 | 1.66 | 3.8 |
| *cg1210* |  | putative membrane protein | 1.00 | 1.04 | 1.33 | 1.16 |
| *cg1271* | *sigE* | RNA polymerase sigma factor, ECF-family | 1.36 | 1.99 | 1.05 | 1.36 |
| *cg1287* |  | hypothetical protein | 1.50 | 1.64 | 1.61 | 1.09 |
| *cg1296* |  | hypothetical protein, putative non-ribosomal peptide synthetase module | 2.64 | 3.14 | 2.69 | 4.44 |
| *cg1313* |  | putative secreted lipoprotein | 1.31 | 1.88 | 1.88 | 2.24 |
| *cg1417* |  | putative acetyltransferase | 3.55 | 4.78 | 1.72 | 2.85 |
| *cg1470* |  | hypothetical protein | 1.09 | 1.03 | 1.84 | 1.1 |
| *cg1555* |  | DNA/RNA helicase, superfamily I | 1.58 | 2.64 | 1.06 | 2.14 |
| *cg1566* |  | hypothetical protein | 1.15 | 1.44 | 1.51 | 1.58 |
| *cg1589* |  | putative secreted protein | 1.54 | 1.83 | 1.85 | 1.54 |
| *cg1590* |  | putative secreted protein | 1.58 | 1.75 | 1.86 | 1.35 |
| *cg1612* |  | putative acetyltransferase | 2.89 | 2.13 | 3.94 | 2.2 |
| *cg1658* |  | putative metabolite permease, MFS-type | 1.57 | 1.29 | 1.46 | 1.6 |
| *cg1660* |  | putative membrane protein | 1.41 | 1.7 | 1.23 | 1.66 |
| *cg1703* |  | putative FAD-dependent pyridine nucleotide-disulfide oxidoreductase | 1.01 | 1.37 | 1.04 | 1.32 |
| *cg1705* | *arsC2* | putative secondary arsenite transporter, arsenical resistance-3 (ACR3) family | 1.39 | 2.09 | 1.52 | 2.1 |
| *cg1706* |  | putative arsenate reductase (arsenical pump modifier) | 1.21 | 1.82 | 1.44 | 1.96 |
| *cg1783* |  | Putative FAD-dependent oxidoreductase, pseudogene (N-terminal fragment) | 1.51 | 1.3 | 1.03 | 3.48 |
| *cg2078* |  | peptide methionine sulfoxide reductase | 1.57 | 2.44 | 1.67 | 2.03 |
| *cg2106* |  | hypothetical protein | 1.92 | 2.99 | 2.16 | 3.01 |
| *cg2136* | *gluA* | ABC-type glutamate transporter, ATPase subunit | 1.18 | 1.1 | 1.58 | 1.01 |
| *cg2157* | *terC* | putative integral membrane export protein, tellurium resistance | 1.34 | 1.58 | 1.22 | 1.53 |
| *cg2194* | *mtr* | putative NADPH-dependent mycothiol reductase | 1.24 | 1.84 | 1.16 | 2.45 |
| *cg2261* | *amtB* | putative secondary ammonium transporter, Amt-family | 1.97 | 2.38 | 1.59 | 4.28 |
| *cg2381* |  | hypothetical protein | 1.15 | 1.49 | 1.02 | 1.21 |
| *cg2411* |  | hypothetical protein, HesB/YadR/YfhF family | 1.29 | 1.68 | 1.38 | 2.04 |
| *cg2429* | *glnA* | glutamate-ammonia ligase | 1.33 | 1.28 | 1.03 | 1.8 |
| *cg2538* |  | alkanal monooxygenase (FMN-linked) | 2.29 | 3.62 | 1.94 | 3.55 |
| *cg2559* | *aceB* | malate synthase | 2.14 | 1.96 | 2.64 | 1.55 |
| *cg2628* | *pcaC* | putative 4-carboxymuconolactone decarboxylase | 5.46 | 5.11 | 1.38 | 1.53 |
| *cg2629* | *pcaB* | putative 3-carboxy-cis,cis-muconate cycloisomerase | 5.36 | 5.13 | 1.52 | 1.37 |
| *cg2630* | *pcaG* | protocatechuate 3,4-dioxygenase, alpha subunit | 5.94 | 5.81 | 2.01 | 1.66 |
| *cg2631* | *pcaH* | protocatechuate 3,4-dioxygenase, beta subunit | 6.82 | 6.43 | 2.77 | 2.15 |
| *cg2782* | *ftn* | ferritin-like protein | 1.92 | 1.6 | 2.49 | 2.08 |
| *cg2788* |  | hypothetical protein | 1.25 | 2.15 | 1.3 | 1.72 |
| *cg2789* | *mrx2* | mycoredoxin 2 | 1.15 | 2.03 | 1.28 | 1.8 |
| *cg2810* |  | putative secondary H+/Na+:glutamate/dicarboxylate symporter, dicarboxylate/amino acid:cation symporter (DAACS) family | 1.80 | 2.9 | 2.9 | 3.4 |
| *cg2838* |  | putative dithiol-disulfide isomerase | 2.52 | 3.37 | 2.47 | 3.97 |
| *cg2870* | *dctA* | putative secondary H+/Na+:C4-dicarboxylate symporter, dicarboxylate/amino acid:cation symporter (DAACS) family | 1.55 | 2.89 | 2.4 | 2.94 |
| *cg3082* |  | putative transcriptional regulator, ArsR-family | 3.19 | 3.56 | 4.23 | 3.72 |
| *cg3083* |  | putative secondary Co2+/Zn2+/Cd2+ efflux transporter, cation diffusion facilitator (CDF) family | 3.02 | 3.63 | 3.9 | 4.09 |
| *cg3084* |  | putative flavoprotein involved in K+ transport | 2.33 | 2.83 | 3.16 | 3.57 |
| *cg3213* |  | putative secreted protein | 4.78 | 5.46 | 1.9 | 2.34 |
| *cg3234* |  | putative metal-dependent amidase/aminoacylase/carboxypeptidase | 1.89 | 2.68 | 1.77 | 2.73 |
| *cg3236* | *msrA* | protein-methionine-S-oxide reductase | 2.04 | 2.66 | 2.18 | 2.6 |
| *cg3272* |  | putative membrane protein | 1.65 | 1.79 | 1.44 | 1.53 |
| *cg3279* |  | putative dehydrogenase, putative pseudogene | 5.07 | 4.27 | 5.14 | 4.66 |
| *cg3280* |  | putative secreted protein | 5.40 | 4.38 | 5.49 | 4.67 |
| *cg3281* |  | putative Cu2+ transporting P-type ATPase | 5.20 | 4.48 | 5.04 | 4.42 |
| *cg3282* |  | putative Cu2+ transporting P-type ATPase | 5.33 | 4.9 | 5.11 | 4.72 |
| *cg3283* |  | hypothetical protein | 5.15 | 4.82 | 4.66 | 4.65 |
| *cg3296* | *tnp19c* | transposase (fragment) | 1.73 | 1.67 | 1.46 | 1.89 |
| *cg3297* | *tnp19b* | transposase (fragment) | 1.15 | 1.97 | 1.29 | 2.04 |
| *cg3303* |  | putative transcriptional regulator, PadR-family | 2.13 | 2.22 | 2.35 | 2.23 |
| *cg3326* |  | hypothetical protein | 1.39 | 1.82 | 1.64 | 2.59 |
| *cg3327* | *dps* | putative starvation-induced DNA protecting protein | 1.33 | 1.55 | 1.83 | 2.73 |
| *cg3333* |  | hypothetical protein | 2.11 | 3.21 | 2.29 | 2.27 |
| *cg3334* |  | putative sugar permease, MFS-type | 1.89 | 3.3 | 2.02 | 2.87 |
| *cg3338* |  | putative membrane protein | 2.12 | 1.65 | 1.42 | 1.18 |
| *cg3344* |  | putative nitroreductase | 1.96 | 2.66 | 1.03 | 2.27 |
| *cg3345* |  | hypothetical protein | 1.11 | 1.09 | 1.03 | 1.12 |
| *cg3374* |  | putative NADH-dependent flavin oxidoreductase | 2.91 | 5.78 | 3.41 | 5.56 |
| *cg3389* |  | putative oxidoreductase | 1.63 | 1.64 | 4.67 | 4.61 |
| *cg3390* |  | putative sugar phosphate isomerase/epimerase | 2.29 | 2.16 | 5.53 | 4.95 |
| *cg3391* | *idhA1* | inositol 2-dehydrogenase | 2.68 | 2.27 | 5.9 | 4.84 |
| *cg3392* | *idhA2* | inositol 2-dehydrogenase | 2.10 | 2.1 | 5.31 | 4.68 |
| *cg3402* |  | putative Hg2+ permease, MerTP-family | 5.68 | 4.81 | 5.29 | 5.16 |
| *cg3405* |  | NADPH:quinone reductase Zn-dependent oxidoreductase | 2.12 | 2.98 | 2.49 | 3.51 |
| *cg3407* |  | putative membrane protein | 1.40 | 2.03 | 1.77 | 2.68 |
| *cg3411* |  | putative Hg2+ permease, MerTP-family | 4.18 | 4.27 | 4.83 | 4.6 |
| *cg4028* |  | hypothetical protein | 5.25 | 4.7 | 5.12 | 2.2 |

Table S4: (p)ppGpp and time-independent down-regulated genes. M-values of genes exhibiting significantly decreased transcript levels in both the parental strain CR099 (WT) and the (p)ppGpp free mutant CR099 Δ*rel*Δ*relS*Δ*relH* (Mut) compared to the respective unstressed initial state after 15 (WT15; Mut15) and 60 minutes (WT60; Mut60) of total starvation.

| Locus tag | Gene name | Product/Function | M-value | | | |
| --- | --- | --- | --- | --- | --- | --- |
|  |  |  | **WT15** | **Mut15** | **WT60** | **Mut60** |
| *cg0591* |  | ABC-type putative iron-siderophore transporter, permease subunit | -1.71 | -1.27 | -1.54 | -1.52 |
| *cg0767* |  | siderophore-interacting protein | -1.06 | -1.34 | -1.96 | -1.62 |
| *cg0769* |  | ABC-type putative iron-siderophore transporter, permease subunit | -1.22 | -1.2 | -1.83 | -1.3 |
| *cg0770* |  | ABC-type putative iron-siderophore transporter, permease subunit | -1.67 | -1.96 | -1.89 | -2.03 |
| *cg0771a* | *Irp1a* | ABC-type putative iron-siderophore transporter, substrate-binding lipoprotein, putative pseudogene | -2.43 | -3.04 | -3.04 | -2.53 |
| *cg0771b* | *Irp1b* | ABC-type putative iron-siderophore transporter, substrate-binding lipoprotein, putative pseudogene | -2.30 | -2.77 | -2.83 | -2.6 |
| *cg0924* |  | ABC-type putative iron-siderophore transporter, substrate-binding lipoprotein | -3.15 | -2.9 | -3.51 | -3.5 |
| *cg0926* |  | ABC-type putative iron-siderophore transporter, permease subunit | -2.60 | -2.49 | -2.97 | -2.74 |
| *cg0927* |  | ABC-type putative iron-siderophore transporter, permease subunit | -2.29 | -1.96 | -2.6 | -2.33 |
| *cg0928* |  | ABC-type putative iron-siderophore transporter, ATPase subunit | -1.62 | -1.89 | -1.87 | -1.77 |
| *cg1292* |  | flavin-containing monooxygenase 3 | -2.40 | -2.03 | -1.85 | -1.56 |
| *cg2087* |  | putative membrane protein | -1.15 | -1.53 | -1.35 | -1.31 |
| *cg2234* |  | ABC-type putative iron(III) dicitrate transporter, substrate-binding lipoprotein | -2.37 | -2.2 | -3.81 | -3.03 |
| *cg2896* |  | putative secreted protein, hypothetical endoglucanase | -2.76 | -2.13 | -2.04 | -1.2 |
| *cg2962* |  | hypothetical protein | -1.47 | -2.49 | -2.32 | -2.2 |
| *cg3247* | *cgtR11* | two-component system, transcriptional response regulator, probalble nitrate/nitrite response | -1.33 | -1.51 | -1.3 | -1.66 |
| *cg3278* | *tnp20a* | transposase | -1.95 | -2.23 | -1.88 | -2.42 |
| *cg3323* | *ino1* | inositol-3-phosphate synthase | -1.18 | -2.55 | -5.92 | -7 |
| *cg3395* | *proP* | putative proline/betaine permease, MFS-type | -2.91 | -3.03 | -2.94 | -3.67 |
| *cg3404* |  | ABC-type putative iron(III) dicitrate transporter, substrate-binding lipoprotein | -1.86 | -1.4 | -2.39 | -1.83 |

Table S5: (p)ppGpp-dependent, time-independent up-regulated genes. Presentation of genes with a significantly increased transcript level only in the parental strain CR099 (WT) and not in the (p)ppGpp free mutant CR099 Δ*rel*Δ*relS*Δ*relH* (Mut) compared to the respective unstressed initial state both after 15 and 60 minutes of total starvation. Comparative listing of M-values for both strains, as well as stress exposure durations.

| Locus tag | Gene name | Product/Function | M-value | | | |
| --- | --- | --- | --- | --- | --- | --- |
|  |  |  | **WT15** | **Mut15** | **WT60** | **Mut60** |
| *cg0038* | *ohr* | putative organic hydroperoxide detoxification protein | 1.29 | 0.75 | 1.65 | 0.86 |
| *cg0205* | *iolH* | myo-inositol catabolism protein | 1.55 | 0.75 | 1.04 | 0.04 |
| *cg0223* |  | putative sugar/metabolite permease, MFS-type | 1.13 | 0.42 | 1.76 | 0.42 |
| *cg0288* |  | ABC-type transporter, membrane and ATPase subunit | 1.10 | 0.86 | 1.56 | 0.67 |
| *cg0567* | *gabD2* | putative succinate-semialdehyde dehydrogenase (NAD(P)(+)) | 1.27 | 0.59 | 1.13 | 0.69 |
| *cg0641* | *fabG2* | putative secreted short-chain dehydrogenase | 1.28 | 0.66 | 2.15 | 0.54 |
| *cg1055* | *menG* | S-Adenosylmethionine:2- demethylmenaquinonemethyltransferase | 1.38 | 0.36 | 1.95 | 0.68 |
| *cg1335* |  | hypothetical protein | 1.06 | 0.74 | 1.58 | 0.88 |
| *cg1336* |  | putative secreted protein | 1.08 | 0.93 | 1.59 | 0.79 |
| *cg1341* | *narI* | respiratory nitrate reductase 2, gamma chain | 1.68 | -0.77 | 1.39 | -0.15 |
| *cg1342* | *narJ* | respiratory nitrate reductase 2, delta chain | 2.00 | -0.59 | 1.44 | -0.4 |
| *cg1343* | *narH* | respiratory nitrate reductase 2, beta chain | 2.44 | -0.04 | 1.38 | 0.1 |
| *cg1344* | *narG* | respiratory nitrate reductase 2, alpha chain | 2.70 | 0.98 | 1.29 | 0.47 |
| *cg1411* | *rbsA* | ABC-type ribose transporter, ATPase subunit | 1.14 | 0.17 | 1.39 | 0.18 |
| *cg1412* | *rbsC* | ABC-type ribose transporter, permease subunit | 1.44 | -0.05 | 1.46 | -0.12 |
| *cg1413* | *rbsB* | ABC-type ribose transporter, substrate-binding lipoprotein | 1.60 | 0.04 | 1.42 | -0.17 |
| *cg1414* | *rbsD* | ABC-type ribose transporter, uncharacterized component | 1.65 | 0.09 | 1.28 | -0.04 |
| *cg2485* | *phoD* | alkaline phosphatase, secreted precursor | 1.33 | 0.77 | 1.1 | 0.24 |
| *cg2610* |  | ABC-type putative dipeptide/oligopeptide transporter, substrate-binding lipoprotein | 1.73 | 0.68 | 2.26 | 0.9 |
| *cg2637* | *benA* | putative benzoate 1,2-dioxygenase, alpha subunit | 1.55 | 0.5 | 3.14 | 0.81 |
| *cg2799* |  | putative secreted protein | 1.53 | 0.91 | 1.03 | 0.4 |
| *cg2938* |  | ABC-type putative dipeptide/oligopeptide transporter, permease subunit | 1.24 | 0.29 | 1.97 | 0.65 |
| *cg2939* |  | ABC-type putative dipeptide/oligopeptide transporter, ATPase subunit | 1.42 | 0.38 | 1.92 | 0.6 |
| *cg2940* |  | ABC-type putative dipeptide/oligopeptide transporter, ATPase subunit | 1.33 | 0.44 | 1.78 | 0.54 |
| *cg3047* | *ackA* | acetate kinase | 1.09 | -0.16 | 1.61 | 0.57 |
| *cg3048* | *pta* | phosphate acetyltransferase | 1.10 | -0.42 | 1.64 | 0.72 |
| *cg3051* |  | putative secreted protein | 1.46 | 0.83 | 1.1 | 0.57 |
| *cg3096* | *ald* | acetaldehyde dehydrogenase (acetylating) | 2.72 | 0.45 | 4.53 | 1.25 |
| *cg3107* | *adhA* | alcohol dehydrogenase | 2.01 | 0.1 | 4.23 | 0.75 |
| *cg3125* | *tctA* | citrate uptake transporter, membrane subunit | 1.25 | 0.96 | 1.7 | 0.67 |
| *cg3195* |  | putative flavin-containing monooxygenase | 2.20 | 0.71 | 4.49 | 1.33 |

Table S6: Genes exclusively up-regulated (p)ppGpp-dependently after 15 minutes of total starvation. Comparative listing of M-values for both parental strain CR099 (WT) and (p)ppGpp^0^-strain CR099 Δ*rel*Δ*relS*Δ*relH* (Mut), as well as stress exposure durations (t_15_; t_60_), compared to the respective unstressed initial state.

| Locus tag | Gene name | Product/Function | M-value | | | |
| --- | --- | --- | --- | --- | --- | --- |
|  |  |  | **WT15** | **Mut15** | **WT60** | **Mut60** |
| *cg0133* |  | putative p-aminobenzoyl-glutamate transporter, anion permease ArsB/NhaD family | 1.44 | 0.91 | 0.8 | 0.02 |
| *cg0134* |  | metal-dependent amidase/aminoacylase/carboxypeptidase, AbgB-like protein | 1.08 | 0.34 | 0.52 | -0.4 |
| *cg0175* |  | putative secreted protein | 1.40 | 0.72 | -0.56 | -0.47 |
| *cg0471* |  | secreted protein | 1.03 | 0.69 | 0.85 | 0.51 |
| *cg0518* | *hemL* | glutamate-1-semialdehyde 2,1-aminomutase, AT class II | 1.06 | 0.93 | 0.26 | 0.38 |
| *cg0519* |  | putative phosphoglycerate mutase | 1.04 | 0.83 | 0.17 | 0.27 |
| *cg0522* | *ccsA* | cytochrome c biogenesis membrane protein, DsbD-family | 1.01 | 0.17 | 0.02 | 0.16 |
| *cg0568* |  | putative secondary aromatic amino acid permease, hydroxy/aromatic amino acid permease (HAAAP) family | 1.09 | 0.25 | 0.92 | 0.46 |
| *cg0664* |  | putative membrane protein | 1.07 | 0.89 | -0.11 | 0.44 |
| *cg0753* |  | putative secreted protein | 1.56 | 0.98 | 0.81 | 0.77 |
| *cg0839* |  | hypothetical protein | 1.13 | 0.21 | 0.88 | 0.26 |
| *cg1419* |  | putative secondary Na+/bile acid symporter, bile acid:Na+ symporter (BASS) family | 1.29 | -0.84 | -0.38 | -0.05 |
| *cg1487* | *leuC* | 3-Isopropylmalate dehydratase, large subunit | 1.61 | -0.56 | 0.83 | 0.25 |
| *cg1488* | *leuD* | 3-Isopropylmalate dehydratase, small subunit | 1.44 | -0.38 | 0.9 | 0.34 |
| *cg1546* | *rbsK1* | putative ribokinase | 1.62 | 0.77 | 0.81 | 0.86 |
| *cg1586* | *argG* | argininosuccinate synthase | 1.88 | 0.74 | -0.35 | 0.96 |
| *cg1665* |  | putative secreted protein | 1.08 | 0.62 | -0.27 | -0.55 |
| *cg1853* | *glpD* | glycerol-3-phosphate dehydrogenase | 1.10 | 0.76 | 0.92 | 0.45 |
| *cg2267* |  | putative membrane protein | 1.13 | 0.74 | 0.72 | 0.74 |
| *cg2545* |  | putative secreted or membrane protein | 1.59 | 0.02 | -0.31 | -0.87 |
| *cg2546* |  | putative secondary C4-dicarboxylate transporter, tripartite ATP-independent transporter (TRAP-T) family | 1.31 | -0.67 | -0.16 | -1.51 |
| *cg3364* | *trpA* | tryptophan synthase alpha chain | 1.38 | 0.19 | 0.16 | 0.28 |

Table S7: Genes exclusively down-regulated (p)ppGpp-dependently after 15 minutes of total starvation. Comparative listing of M-values for both parental strain CR099 (WT) and (p)ppGpp^0^-strain CR099 Δ*rel*Δ*relS*Δ*relH* (Mut), as well as stress exposure durations (t_15_; t_60_), compared to the respective unstressed initial state.

| Locus tag | Gene name | Product/Function | M-value | | | |
| --- | --- | --- | --- | --- | --- | --- |
|  |  |  | **WT15** | **Mut15** | **WT60** | **Mut60** |
| *cg0898* | pdxS | pyridoxal 5'-phosphate (PLP) synthase subunit | -1.09 | 0.59 | 0.41 | 0.43 |
| *cg0899* | pdxT | pyridoxal 5'-phosphate (PLP) synthase subunit, glutamine amidotransferase | -1.17 | 0.48 | 0.1 | 0.21 |
| *cg1091* |  | hypothetical protein | -1.41 | -0.71 | -0.58 | 0.4 |
| *cg1290* | metE | 5-Methyltetrahydropteroyltriglutamate- homocysteine methyltransferase | -1.09 | 0.96 | 0.94 | 1.5 |
| *cg1424* | lysE | L-Lysine efflux permease, L-lysine exporter (LysE) family | -1.25 | -0.22 | -0.98 | 0.39 |
| *cg1464* |  | putative transcriptional regulator, HTH_3-family | -1.08 | -0.18 | -0.53 | 0.4 |
| *cg1763* | sufD | FeS cluster assembly protein, SufD-family | -2.23 | -0.89 | -0.63 | -0.13 |
| *cg2831* | ramA | putative transcriptional regulator, LuxR-family, Nif-specific | -1.38 | -0.94 | -0.63 | -0.23 |
| *cg3186* | cmt2 | trehalose corynomycolyl transferase | -1.19 | -0.89 | 0 | -0.32 |

Table S8: Genes exclusively up-regulated (p)ppGpp-dependently after 60 minutes of total starvation. Comparative listing of M-values for both parental strain CR099 (WT) and (p)ppGpp^0^-strain CR099 Δ*rel*Δ*relS*Δ*relH* (Mut), as well as stress exposure durations (t_15_; t_60_), compared to the respective unstressed initial state.

| Locus tag | Gene name | Product/Function | M-value | | | |
| --- | --- | --- | --- | --- | --- | --- |
|  |  |  | **WT15** | **Mut15** | **WT60** | **Mut60** |
| *cg0012* | *ssuR* | transcriptional activator of sulfonate(ester) utilization, ROK-family | -0.59 | -1.13 | 5.57 | 0.19 |
| *cg0018* |  | putative membrane protein | 0.56 | 0.65 | 4.76 | 0.89 |
| *cg0110* | *lip2* | triacylglycerol lipase | 0.85 | 0.64 | 1.04 | 0.98 |
| *cg0120* |  | putative hydrolase | -0.18 | -0.48 | 1.37 | 0.82 |
| *cg0143* | *mtlD* | mannitol-1-phosphate 5-dehydrogenase | 0.38 | 0.4 | 1.45 | 0.94 |
| *cg0156* | *cysR* | transcriptional activator of assimilatory sulfate reduction, ROK-family | -0.91 | -1.58 | 2.59 | -0.1 |
| *cg0171* |  | putative secreted protein | 0.49 | 0.12 | 1.08 | 0.76 |
| *cg0192* |  | hypothetical protein | 0.18 | -0.24 | 1.14 | 0.34 |
| *cg0208* |  | hypothetical protein | 0.09 | -0.02 | 1.21 | 0.13 |
| *cg0228* |  | putative two-component system, sensory histidine kinase, putative pseudogene | 0.10 | -0.28 | 2.06 | 0.63 |
| *cg0232* |  | putative secreted protein | 0.55 | -0.52 | 1.05 | -0.13 |
| *cg0233* |  | hypothetical protein | 0.49 | -0.07 | 1.05 | -0.05 |
| *cg0287* |  | hypothetical protein | 0.75 | 0.87 | 1.1 | 0.81 |
| *cg0344* | *fabG1* | 3-Oxoacyl-(acyl-carrier protein) reductase | 0.14 | -0.12 | 2.11 | 0.93 |
| *cg0346* | *fadE* | glutaryl-CoA dehydrogenase | 0.25 | 0.05 | 1.24 | 0.46 |
| *cg0347* |  | putative dehydratase, MaoC-family | -0.19 | -0.01 | 1.39 | 0.72 |
| *cg0606* |  | putative membrane protein | 0.91 | 0.71 | 1.24 | 0.26 |
| *cg0636* |  | putative membrane protein | 0.84 | 0.77 | 1.68 | 0.61 |
| *cg0637* | *betB* | putative betaine aldehyde dehydrogenase (BADH) | 0.41 | 0.16 | 1.03 | 0.28 |
| *cg0638* |  | hypothetical protein | 0.52 | 0.26 | 1.28 | -0.12 |
| *cg0639* |  | putative ferredoxin reductase | 0.78 | 0.45 | 1.55 | 0.3 |
| *cg0640* | *fdxB* | 2Fe-2S ferredoxin | 0.35 | 0.07 | 1.46 | 0.38 |
| *cg0644* |  | PEP/pyruvate-binding pyruvate phosphate dikinase | 0.99 | 0.25 | 2.67 | 1.05 |
| *cg0735* | *metI* | ABC-type methionine transporter, permease subunit | 0.15 | 0.59 | 1.26 | -0.01 |
| *cg0736* | *metN* | ABC-type methionine transporter, ATPase subunit | 0.13 | 0.62 | 1.33 | -0.06 |
| *cg0737* | *metQ* | ABC-type methionine transporter, substrate-binding lipoprotein | -1.16 | -1.66 | 3.79 | 0.16 |
| *cg0745* |  | putative NAD-dependent protein deacetylase, SIR2-family | 0.43 | 0.49 | 1.14 | 0.68 |
| *cg0754* | *metX* | homoserine O-acetyltransferase | -1.39 | -2.67 | 2.91 | -0.14 |
| *cg0755* | *metY* | O-Acetylhomoserine sulfhydrylase | -1.39 | -2.47 | 4.71 | 0.27 |
| *cg0795* |  | putative oxidoreductase | 0.60 | 0.56 | 1.35 | 0.51 |
| *cg0796* | *prpD1* | putative (2-methyl) citrate dehydratase | 0.31 | -1.06 | 2.09 | 0.33 |
| *cg0797* | *prpB1* | putative (methyl)isocitrate lyase | 0.39 | -1.37 | 2.27 | 0.16 |
| *cg0798* | *prpC1* | putative (methyl)citrate synthase | 0.67 | -1.39 | 2.3 | 0.03 |
| *cg0834* | *tusE* | ABC-type trehalose transporter, substrate-binding lipoprotein | 0.53 | -0.38 | 1.08 | -0.32 |
| *cg0838* |  | putative helicase | 0.98 | -1.3 | 1.74 | 0.29 |
| *cg0858* |  | putative secreted protein | -1.21 | -1.65 | 1.1 | 0.31 |
| *cg0875* |  | hypothetical protein | 0.82 | 0.85 | 1.17 | 0.4 |
| *cg1043* |  | hypothetical protein | -0.08 | -0.95 | 1.11 | 0.17 |
| *cg1073* |  | putative lactoylglutathione lyase | 0.06 | -0.15 | 1.07 | 0.54 |
| *cg1087* |  | putative membrane protein | 0.29 | -0.15 | 1.98 | 0.97 |
| *cg1088* |  | ABC-type putative multidrug transporter, ATPase and permease subunit | 0.27 | -0.26 | 1.87 | 0.89 |
| *cg1090* | *ggtB* | putative gamma-glutamyltranspeptidase | 0.69 | 0.11 | 2.41 | 0.93 |
| *cg1109* |  | hypothetical protein | 0.39 | 0.48 | 1.31 | 0.78 |
| *cg1147* | *ssuI* | FMN reductase (NADPH) | -0.65 | -0.54 | 3.13 | 0.14 |
| *cg1151* | *seuA* | FMNH2-dependent monooxygenase, involved in sulfonate ester degradation | -0.18 | 0.58 | 4.48 | 0.67 |
| *cg1152* | *seuB* | FMNH2-dependent monooxygenase, involved in sulfonate ester degradation | 0.84 | -0.04 | 4.31 | -0.46 |
| *cg1153* | *seuC* | putative FMNH2-dependent monooxygenase | 0.83 | 0.31 | 3.7 | 0.17 |
| *cg1156* | *ssuD2* | FMNH2-dependent aliphatic sulfonate monooxygenase | -0.33 | -0.41 | 3.41 | 0.65 |
| *cg1200* |  | hypothetical protein | 0.88 | 0.72 | 1.05 | 0.28 |
| *cg1216* | *nadA* | quinolinate synthetase, subunit A | 0.69 | 0.39 | 1.17 | 0.23 |
| *cg1218* |  | ADP-ribose pyrophosphatase | 0.67 | 0.45 | 1 | 0.05 |
| *cg1224* |  | hypothetical protein | -0.76 | -1.41 | 1.14 | 0.49 |
| *cg1314* | *putP* | putative Na+/proline symporter, solute:sodium symporter (SSS) family | 0.60 | 0.37 | 1.35 | 0.8 |
| *cg1377* | *ssuC* | ABC-type aliphatic sulfonate transporter, permease subunit | 0.00 | 0.26 | 5.14 | -0.22 |
| *cg1379* | *ssuB* | ABC-type aliphatic sulfonate transporter, ATPase subunit | 0.14 | -0.33 | 4.88 | -0.58 |
| *cg1380* | *ssuA* | ABC-type aliphatic sulfonate transporter, substrate-binding lipoprotein | -0.24 | -0.04 | 3.71 | 0.17 |
| *cg1386* | *fixA* | putative electron transfer flavoprotein, beta subunit | -0.24 | -0.19 | 1.35 | 0.21 |
| *cg1387* | *fixB* | putative electron transfer flavoprotein, alpha subunit | -0.26 | -0.03 | 1.15 | 0.13 |
| *cg1410* | *rbsR* | transcriptional repressor of the ribose importer RbsACBD, LacI-family | 0.78 | -0.1 | 1.42 | 0.46 |
| *cg1426* | *gst* | putative glutathione S-transferase | 0.16 | 0.44 | 1.04 | 0.55 |
| *cg1478* |  | hypothetical protein | -0.28 | -1.13 | 2.32 | 0.78 |
| *cg1484* |  | putative secreted protein | 0.66 | 0.14 | 1.35 | 0.3 |
| *cg1613* | *sseA2* | rhodanese-related sulfurtransferase | 0.84 | 0.36 | 1.36 | 0.99 |
| *cg1701* | *metH* | methionine synthase | -0.74 | -0.56 | 1.05 | 0.47 |
| *cg1739* |  | hypothetical protein | -1.53 | -1.17 | 1.79 | 0.3 |
| *cg1806* | *metK* | methionine adenosyltransferase | -1.24 | -1.66 | 1.07 | -0.03 |
| *cg2118* |  | transcriptional regulator protein, DeoR-family | 0.40 | -0.7 | 1.39 | -0.12 |
| *cg2119* | *fruK* | 1-Phosphofructokinase | 0.60 | -0.35 | 1.33 | -0.12 |
| *cg2120* | *ptsF* | phosphotransferase system (PTS), fructose-specific enzyme IIABC component | 0.87 | -0.02 | 1.37 | -0.15 |
| *cg2202* |  | ABC-type transporter, permease subunit | 0.48 | 0 | 1.09 | -0.27 |
| *cg2313* | *idhA3* | myo-inositol 2-dehydrogenase | 0.43 | 0.3 | 1.02 | 0.02 |
| *cg2438* |  | hypothetical protein | 0.58 | 0.18 | 1.2 | 0.6 |
| *cg2500* |  | putative transcriptional regulator, ArsR-family | 0.74 | 0.01 | 1.19 | 0.39 |
| *cg2565* |  | hypothetical protein | 0.41 | -0.46 | 1.52 | 0.44 |
| *cg2566* |  | putative secreted protein | 0.80 | 0.83 | 1.08 | 0.54 |
| *cg2634* | *catC* | putative muconolactone delta-isomerase | 0.64 | 0.25 | 1.2 | 0.29 |
| *cg2635* | *catB* | putative muconate cycloisomerase | 0.62 | 0.32 | 1.27 | 0.4 |
| *cg2639* | *benC* | putative benzoate 1,2-dioxygenase, electron transfer subunit | 0.59 | 0.92 | 1.72 | 0.6 |
| *cg2641* | *benR* | putative transcriptional regulator, LuxR-family | 0.47 | 0.72 | 1.2 | 0.79 |
| *cg2642* | *benK* | putative MFS-type benzoate permease | 0.59 | -0.21 | 1.93 | 0.49 |
| *cg2643* | *benE* | putative secondary benzoate symporter, benzoate:H+ symporter (BenE) family | 0.71 | 0.3 | 1.66 | 0.5 |
| *cg2674* |  | alkylhydroperoxidase, AhpD-family | -0.69 | -0.91 | 1.15 | -1.02 |
| *cg2675* |  | ABC-type putative dipeptide/oligopeptide transporter, ATPase subunit | -0.76 | -1.82 | 3.73 | -1.09 |
| *cg2676* |  | ABC-type putative dipeptide/oligopeptide transporter, permease subunit | -0.96 | -2.45 | 4.63 | -1.07 |
| *cg2677* |  | ABC-type putative dipeptide/oligopeptide transporter, permease subunit | -1.06 | -2.73 | 4.68 | -0.67 |
| *cg2678* |  | ABC-type putative dipeptide/oligopeptide transporter, substrate-binding lipoprotein | -0.77 | -2.66 | 4.99 | -0.43 |
| *cg2679* |  | hypothetical protein | -0.43 | -1.19 | 2.5 | -0.58 |
| *cg2687* | *metB* | cystathionine gamma-synthase | -0.81 | -1.32 | 2.52 | 0.64 |
| *cg2694* |  | hypothetical protein | 0.70 | 0.66 | 1.14 | 0.05 |
| *cg2833* | *cysK* | cysteine synthase | -1.31 | -3.63 | 3.78 | -1.57 |
| *cg2837* | *sucC* | succinate-CoA ligase (ADP-forming), beta subunit | -0.21 | -0.77 | 2.16 | 0.82 |
| *cg2925* | *ptsS* | phosphotransferase system (PTS), sucrose-specific enzyme IIBCA component | 0.49 | -0.1 | 1.11 | 0.2 |
| *cg2937* |  | ABC-type putative dipeptide/oligopeptide transporter, substrate-binding lipoprotein | 0.91 | 0.01 | 1.73 | 0.52 |
| *cg2966* |  | putative phenol 2-monooxygenase | 0.43 | -0.02 | 1.99 | 0.7 |
| *cg3112* | *cysZ* | sulfate transporter | -0.60 | -1.98 | 8.3 | -1.01 |
| *cg3113* | *cysY* | sirohydrochlorin ferrochelatase | -0.72 | -1.97 | 8.66 | -0.96 |
| *cg3114* | *cysN* | sulfate adenylyltransferase subunit 1 | -1.21 | -1.78 | 7.93 | -1.1 |
| *cg3115* | *cysD* | sulfate adenylyltransferase subunit 2 | -0.85 | -2.85 | 8 | -0.52 |
| *cg3116* | *cysH* | adenosine phosphosulfate reductase | -1.34 | -3.39 | 7.96 | -0.24 |
| *cg3117* | *cysX* | ferredoxin-like protein, involved in electron-transfer | -1.40 | -3.74 | 8.23 | 0.04 |
| *cg3118* | *cysI* | ferredoxin-sulfite reductase | -1.55 | -3.62 | 8.36 | 0.26 |
| *cg3119* | *fpr2* | ferredoxin-NADP(+) reductase | -0.55 | -2.56 | 9.18 | 0.77 |
| *cg3121* |  | putative membrane protein | 0.23 | -0.06 | 1.3 | 0.64 |
| *cg3122* |  | hypothetical protein, PhnB-family | 0.22 | -1.07 | 1.7 | 0.66 |
| *cg3126* |  | citrate uptake transporter, membrane subunit | 0.96 | 0.77 | 1.38 | 0.9 |
| *cg3127* | *tctC* | citrate uptake transporter, substrate binding protein | 0.67 | -0.12 | 1.18 | 0.79 |
| *cg3131* |  | acetylornithine deacetylase | -0.77 | -1.4 | 2.39 | -0.67 |
| *cg3132* |  | putative membrane protein | -0.33 | -0.56 | 2.39 | -0.43 |
| *cg3212* |  | putative carboxymuconolactone decarboxylase subunit | 0.23 | -0.37 | 1.39 | -0.56 |
| *cg3216* | *gntP* | putative secondary gluconate symporter, gluconate:H+ symporter (GntP) family | 0.91 | 0.46 | 2.51 | 1.25 |
| *cg3285* | *cgtR9* | two-component system, transcriptional response regulator | 0.59 | -0.22 | 1.24 | -0.06 |
| *cg3352* | *nagR* | transcriptional activator of gentisate catabolism, IclR-family | -0.05 | -1.06 | 1.01 | 0.87 |
| *cg3399* |  | permease, MFS-type | 0.24 | -0.72 | 6.34 | 0.6 |

Table S9: Genes exclusively down-regulated (p)ppGpp-dependently after 60minutes of total starvation. Comparative listing of M-values for both parental strain CR099 (WT) and (p)ppGpp^0^-strain CR099 Δ*rel*Δ*relS*Δ*relH* (Mut), as well as stress exposure durations (t_15_; t_60_), compared to the respective unstressed initial state.

| Locus tag | Gene name | Product/Function | M-value | | | |
| --- | --- | --- | --- | --- | --- | --- |
|  |  |  | **WT15** | **Mut15** | **WT60** | **Mut60** |
| *cg0004* | *dnaN* | DNA polymerase III, beta chain | -0.61 | -0.5 | -1.03 | -0.76 |
| *cg0076* |  | hypothetical protein | -0.41 | -0.27 | -1.26 | -0.68 |
| *cg0167* |  | putative membrane protein, DUF81-family | 0.09 | 0.85 | -1.06 | 0.2 |
| *cg0243* |  | putative membrane protein | -0.50 | -0.6 | -1.04 | -0.69 |
| *cg0244* |  | putative membrane protein | -0.57 | -0.71 | -1.27 | -0.78 |
| *cg0245* |  | hypothetical protein | -0.62 | -0.79 | -1.31 | -0.88 |
| *cg0277* | *dccT* | secondary Na+:succinate/malate/fumarate symporter, divalent anion:Na+ symporter (DASS) family | 0.36 | 0.85 | -1.06 | -0.63 |
| *cg0286* |  | putative membrane protein | -0.45 | 0.35 | -1.38 | -0.01 |
| *cg0321* | *mnhG* | putative Na+/H+ antiporter, membrane subunit | -0.61 | -0.42 | -1.05 | -0.33 |
| *cg0349* |  | putative metal-dependent hydrolase | -0.41 | -0.01 | -1.28 | -0.49 |
| *cg0405* |  | ABC-type putative iron(III) dicitrate transporter, substrate-binding lipoprotein | -0.76 | -0.59 | -1.13 | -0.97 |
| *cg0411* |  | putative membrane protein | -0.42 | 0.22 | -2 | -0.67 |
| *cg0442* | *galU2* | putative UTP-glucose-1-phosphate uridylyltransferase | 0.15 | 0.23 | -1.26 | -0.43 |
| *cg0457* | *purU* | putative formyltetrahydrofolate deformylase | -0.63 | -0.05 | -1.06 | -0.68 |
| *cg0478* |  | hypothetical protein | -0.28 | -0.59 | -1.83 | -1 |
| *cg0510* | *hemD* | uroporphyrinogen-III synthase | 0.01 | 0.59 | -1.1 | -0.22 |
| *cg0545* | *pitA* | putative phosphate/sulfate transporter, inorganic phosphate transporter (PiT) family | -0.19 | -0.36 | -1.27 | -0.99 |
| *cg0557* |  | putative FAD-linked oxidoreductase | -0.49 | -0.42 | -1.01 | -0.74 |
| *cg0563* | *rplK* | 50S ribosomal protein L11 | -0.19 | 0.7 | -1.42 | 0.19 |
| *cg0564* | *rplA* | 50S ribosomal protein L1 | -0.18 | 0.74 | -1.38 | 0.17 |
| *cg0572* | *rplJ* | 50S ribosomal protein L10 | -0.10 | 0.6 | -1.5 | -0.07 |
| *cg0573* | *rplL* | putative 50S ribosomal protein L7/L12 | -0.18 | 0.49 | -1.61 | 0.01 |
| *cg0581* | *rpsL* | 30S ribosomal protein S12 | -0.21 | 0.97 | -1.33 | 0.24 |
| *cg0582* | *rpsG* | 30S ribosomal protein S7 | -0.21 | 1.04 | -1.32 | 0.18 |
| *cg0594* | *rplC* | 50S ribosomal protein L3 | 0.00 | 0.91 | -1.05 | 0.3 |
| *cg0596* | *rplD* | 50S ribosomal protein L4 | -0.10 | 0.89 | -1.18 | 0.17 |
| *cg0597* | *rplW* | 50S ribosomal protein L23 | -0.06 | 0.84 | -1.21 | 0.11 |
| *cg0598* | *rplB* | 50S ribosomal protein L2 | -0.10 | 0.84 | -1.25 | 0.05 |
| *cg0599* | *rpsS* | 30S ribosomal protein S19 | -0.10 | 0.73 | -1.34 | 0.07 |
| *cg0600* | *rplV* | 50S ribosomal protein L22 | -0.09 | 0.75 | -1.28 | 0.02 |
| *cg0601* | *rpsC* | 30S ribosomal protein S3 | -0.06 | 0.77 | -1.26 | -0.04 |
| *cg0602* | *rplP* | 50S ribosomal protein L16 | -0.08 | 0.79 | -1.31 | -0.07 |
| *cg0603* | *rpmC* | 50S ribosomal protein L29 | -0.09 | 0.84 | -1.28 | -0.09 |
| *cg0604* | *rpsQ* | 30S ribosomal protein S17 | -0.18 | 0.73 | -1.56 | 0.03 |
| *cg0608* | *rplN* | 50S ribosomal protein L14 | -0.13 | 0.65 | -1.1 | 0.01 |
| *cg0609* | *rplX* | 50S ribosomal protein L24 | -0.18 | 0.65 | -1.17 | 0.05 |
| *cg0610* | *rplE* | 50S ribosomal protein L5 | -0.19 | 0.72 | -1.2 | 0.05 |
| *cg0621* |  | putative integral membrane protein | -0.21 | -0.65 | -1.29 | -0.71 |
| *cg0622* |  | ABC-type putative cobalt transporter, ATPase subunit | 0.04 | -0.18 | -1.05 | -0.6 |
| *cg0623* |  | ABC-type putative cobalt transporter, permease subunit | -0.10 | 0.15 | -1.13 | -0.24 |
| *cg0628* | *rpsH* | 30S ribosomal protein S8 | -0.42 | 0.5 | -1.47 | -0.06 |
| *cg0629* | *rplF* | 50S ribosomal protein L6 | -0.45 | 0.53 | -1.5 | -0.1 |
| *cg0630* | *rplR* | 50S ribosomal protein L18 | -0.38 | 0.6 | -1.51 | 0 |
| *cg0631* | *rpsE* | 30S ribosomal protein S5 | -0.38 | 0.63 | -1.53 | 0.01 |
| *cg0632* | *rpmD* | 50S ribosomal protein L30 | -0.34 | 0.62 | -1.45 | -0.05 |
| *cg0634* | *rplO* | 50S ribosomal protein L15 | -0.36 | 0.58 | -1.51 | -0.01 |
| *cg0651* | *infA* | translation initiation factor IF-1 | -0.24 | 0.47 | -1.25 | -0.15 |
| *cg0652* | *rpsM* | 30S ribosomal protein S13 | -0.29 | 0.6 | -1.46 | -0.21 |
| *cg0653* | *rpsK* | 30S ribosomal protein S11 | -0.31 | 0.55 | -1.51 | -0.14 |
| *cg0654* | *rpsD* | 30S ribosomal protein S4 | -0.33 | 0.59 | -1.49 | -0.19 |
| *cg0655* | *rpoA* | DNA-directed RNA polymerase, alpha subunit | -0.33 | 0.55 | -1.56 | -0.12 |
| *cg0656* | *rplQ* | 50S ribosomal protein L17 | -0.38 | 0.62 | -1.58 | 0.05 |
| *cg0657* | *truA* | tRNA pseudouridine synthase A | -0.16 | 0.49 | -1.02 | -0.24 |
| *cg0673* | *rplM* | 50S ribosomal protein L13 | -0.44 | 0.58 | -1.36 | -0.09 |
| *cg0674* | *rpsI* | 30S ribosomal protein S9 | -0.45 | 0.61 | -1.31 | -0.07 |
| *cg0732* |  | ABC-type transporter, permease subunit | -0.16 | -0.27 | -1.62 | -0.64 |
| *cg0733* |  | ABC-type transporter, ATPase subunit | -0.27 | 0.01 | -1.64 | -0.38 |
| *cg0861* | *tmk* | putative thymidylate kinase | -0.44 | -0.25 | -1.02 | -0.39 |
| *cg0938* |  | cold shock protein | -0.48 | -0.08 | -1.12 | -0.55 |
| *cg0988* | *rpsR* | 30S ribosomal protein S18 | -0.04 | 0.79 | -1.34 | -0.07 |
| *cg0989* | *rpsN* | 30S ribosomal protein S14 | -0.06 | 0.78 | -1.18 | -0.07 |
| *cg0990* | *rpmG* | 50S ribosomal protein L33 | -0.04 | 0.79 | -1.19 | -0.15 |
| *cg0991* | *rpmB* | 50S ribosomal protein L28 | -0.06 | 0.8 | -1.25 | -0.13 |
| *cg0994* | *rpmE* | putative 50S ribosomal protein L31 | 0.02 | 0.56 | -1.03 | -0.16 |
| *cg0995* | *rpmF* | 50S ribosomal protein L32 | -0.02 | 0.63 | -1.14 | -0.11 |
| *cg1072* | *rplY* | ribosomal protein L25 (general stress protein Ctc) | -0.29 | 0.53 | -1.18 | -0.19 |
| *cg1133* | *glyA* | serine hydroxymethyltransferase | -0.41 | 0.04 | -1.03 | -0.33 |
| *cg1257* | *aroP* | aromatic amino acids uptake system | -0.48 | 0.04 | -1.06 | -0.24 |
| *cg1279* |  | putative secreted protein | -0.67 | -0.56 | -1.42 | -0.75 |
| *cg1291* |  | putative membrane protein | 0.44 | 2.24 | -1.36 | 1.3 |
| *cg1304* |  | putative secreted protein | -0.41 | -0.44 | -1.31 | -0.96 |
| *cg1305* |  | putative secondary proline transporter, amino acid-polyamine-organocation (APC) family | -0.18 | 0.46 | -1.83 | -0.73 |
| *cg1325* |  | hypothetical protein | -0.67 | 0.28 | -1.09 | -0.98 |
| *cg1356* |  | putative rRNA or tRNA methylase | 0.26 | -0.17 | -1.05 | -0.15 |
| *cg1360* |  | putative membrane protein | -0.08 | 0.44 | -1.04 | -0.11 |
| *cg1361* |  | hypothetical protein | -0.36 | 0.6 | -1.06 | 0.21 |
| *cg1362* | *atpB* | ATP synthase F0, A chain | -0.36 | -0.41 | -1.52 | -0.63 |
| *cg1363* | *atpE* | ATP synthase F0, C chain | -0.39 | -0.41 | -1.6 | -0.57 |
| *cg1364* | *atpF* | ATP synthase F0, B chain | -0.37 | -0.38 | -1.55 | -0.55 |
| *cg1365* | *atpH* | ATP synthase F1, delta subunit | -0.31 | -0.34 | -1.57 | -0.57 |
| *cg1366* | *atpA* | ATP synthase F1, alpha chain | -0.33 | -0.21 | -1.63 | -0.63 |
| *cg1367* | *atpG* | ATP synthase F1, gamma chain | -0.40 | -0.2 | -1.67 | -0.64 |
| *cg1368* | *atpD* | ATP synthase F1, beta chain | -0.47 | -0.12 | -1.67 | -0.63 |
| *cg1369* | *atpC* | ATP synthase F1, epsilon chain | -0.42 | -0.01 | -1.63 | -0.52 |
| *cg1506* |  | putative membrane protein | -0.89 | -0.92 | -1.09 | -0.15 |
| *cg1563* | *infC* | translation initiation factor IF-3 | -0.33 | 0.76 | -1.18 | 0.27 |
| *cg1564* | *rpmI* | 50S ribosomal protein L35 | -0.29 | 0.93 | -1.17 | 0.26 |
| *cg1565* | *rplT* | 50S ribosomal protein L20 | -0.36 | 0.98 | -1.31 | 0.33 |
| *cg1626* |  | hypothetical protein | 0.72 | 1.66 | -1.18 | 0.85 |
| *cg1668* |  | putative membrane protein | -0.48 | -0.87 | -1.41 | -0.68 |
| *cg1669* |  | putative secreted protein | -0.45 | -0.64 | -1.3 | -0.78 |
| *cg1670* |  | hypothetical protein | -0.40 | -0.57 | -1.08 | -0.86 |
| *cg1710* | *bacA* | putative undecaprenol kinase | -0.52 | -0.3 | -1.12 | -0.26 |
| *cg1767* |  | ABC-type putative multidrug transporter, ATPase subunit | -0.19 | 0.04 | -1.17 | -0.46 |
| *cg1769* | *ctaA* | cytochrome oxidase assembly protein | -0.13 | 0.44 | -1.49 | -0.04 |
| *cg1773* | *ctaB* | polyprenyltransferase, cytochrome oxidase assembly factor | -0.38 | 0.01 | -1.57 | -0.85 |
| *cg1874* |  | putative membrane protein | -0.48 | -0.21 | -1.02 | -0.13 |
| *cg2079* |  | hypothetical protein, chlorite dismutase family | -0.52 | -0.26 | -1.15 | -0.91 |
| *cg2125* | *uraA* | putative xanthine/uracil symporter, nucleobase:cation symporter-2 (NCS2) family | 0.23 | 0.57 | -1.28 | -0.15 |
| *cg2134* |  | putative membrane protein | -0.71 | -0.56 | -1.43 | -0.6 |
| *cg2135* | *miaB* | tRNA methylthiotransferase | -0.20 | 0.37 | -1.39 | -0.45 |
| *cg2167* | *rpsO* | 30S ribosomal protein S15 | -0.33 | -0.02 | -1.33 | -0.08 |
| *cg2178* | *nusA* | putative transcriptional termination/antitermination factor | -0.28 | 0.21 | -1.23 | -0.39 |
| *cg2235* | *rplS* | 50S ribosomal protein L19 | -0.08 | 0.37 | -1.11 | 0.22 |
| *cg2253* | *rpsP* | 30S ribosomal protein S16 | -0.37 | 0.3 | -1.44 | 0.24 |
| *cg2336* |  | putative secreted protein | -0.52 | -0.61 | -1.05 | -0.89 |
| *cg2353* |  | hypothetical protein disrupted by insertion of ISCg2e | -0.65 | -0.65 | -1.09 | -0.07 |
| *cg2573* | *rpsT* | 30S ribosomal protein S20 | -0.31 | 1.04 | -1.33 | 0.23 |
| *cg2590* |  | putative xanthine/uracil symporter, nucleobase:cation symporter-2 (NCS2) family | 0.03 | 0.58 | -1.04 | -0.14 |
| *cg2594* | *rpmA* | 50S ribosomal protein L27 | -0.19 | 0.45 | -1.17 | -0.08 |
| *cg2595* | *rplU* | 50S ribosomal protein L21 | -0.17 | 0.41 | -1.08 | -0.12 |
| *cg2647* | *tig* | putative Trigger factor,invoved in cell division | -0.12 | 0.22 | -1.39 | -0.2 |
| *cg2699* |  | putative membrane protein | -0.39 | 0.15 | -1.17 | -0.52 |
| *cg2709* |  | Putative polyprenol phosphate mannosyl transferase, putative pseudogene (C-terminal fragment) | -0.42 | -0.23 | -1.7 | -0.51 |
| *cg2739* |  | putative multidrug efflux permease, MFS-type | 0.42 | 1.53 | -1.07 | 0.12 |
| *cg2791* | *rpmJ* | 50S ribosomal protein L36 | -0.11 | 0.25 | -1.18 | 0.05 |
| *cg2954* | *cynT* | carbonate dehydratase | -0.28 | 0.11 | -1.06 | -0.6 |
| *cg3063* | *purA* | adenylosuccinate synthase | -0.91 | -0.98 | -1.15 | -0.87 |
| *cg3133* |  | ABC-type putative cobalt/sugar transporter, ATPase subunit | 0.70 | 1.14 | -1.07 | 0.08 |
| *cg3141* | *hmp* | globin-like flavohemoprotein, putative nitric oxide dioxygenase | -0.15 | 0.22 | -1.46 | -0.23 |
| *cg3158* | *nagA2* | putative beta-glucosidase | -0.15 | 0.06 | -1.42 | -0.55 |
| *cg3240* |  | putative multidrug efflux permease, MFS-type | 0.07 | -0.51 | -1.59 | -0.79 |
| *cg3243* |  | hypothetical protein, RecB-family nuclease | -0.28 | -0.02 | -1.6 | -0.67 |
| *cg3321* |  | ABC-type transporter, ATPase and permease subunit | -0.73 | -0.73 | -1.32 | -0.08 |
| *cg3322* |  | putative secreted membrane-fusion protein | -0.83 | -0.3 | -1.37 | -0.23 |
| *cg3382* |  | putative dipeptide/tripeptide:H+ transporter, proton-dependent oligopeptide transporter (POT) family | -0.39 | -0.13 | -1.73 | -0.83 |
| *cg3431* | *rnpA* | ribonuclease P | 0.17 | 0.29 | -1.06 | -0.23 |
| *cg3434* |  | hypothetical protein | -0.56 | -0.75 | -1 | -0.56 |
| *cg4018* |  | hypothetical protein in strongly transcribed region | -0.16 | -0.19 | -1.16 | -0.22 |

Table S10: Genes exclusively up-regulated in the (p)ppGpp^0^-strain CR099 Δ*rel*Δ*relS*Δ*relH* after 15 minutes of total starvation. Comparative listing of M-values for both parental strain CR099 (WT) and (p)ppGpp^0^-strain CR099 Δ*rel*Δ*relS*Δ*relH* (Mut), as well as stress exposure durations (t_15_; t_60_), compared to the respective unstressed initial state.

| Locus tag | Gene name | Product/Function | M-value | | | |
| --- | --- | --- | --- | --- | --- | --- |
|  |  |  | **WT15** | **Mut15** | **WT60** | **Mut60** |
| *cg0083* |  | putative nicotinamide mononucleotide uptake permease, nicotinamide mononucleotide (NMN) uptake permease (PnuC) family | 0.91 | 1.02 | 0.88 | 0.68 |
| *cg0182* | *tagA2* | DNA-3-methyladenine glycosylase I | 0.73 | 1.23 | 0.37 | 0.85 |
| *cg0215* | *cspA* | cold-shock protein A | 0.72 | 1.39 | -0.48 | 0.01 |
| *cg0283* |  | putative membrane protein | 0.44 | 1.11 | 0.05 | 0.76 |
| *cg0293* |  | hypothetical protein | 0.56 | 1.08 | 0.91 | 0.92 |
| *cg0328* |  | hypothetical protein | 0.44 | 1.35 | 0.03 | 0.8 |
| *cg0390* |  | putative multidrug efflux permease, MFS-type | 0.87 | 1.02 | 0.23 | 0.51 |
| *cg0407* |  | putative secreted protein | 0.47 | 1.16 | 0.34 | 0.9 |
| *cg0461* |  | hypothetical protein | 0.58 | 1.63 | 0.5 | 1.08 |
| *cg0485* |  | hypothetical protein | 0.58 | 1.11 | -0.71 | 0.74 |
| *cg0497* | *hemA* | glutamyl-tRNA reductase | 0.55 | 1.19 | -0.16 | 0.79 |
| *cg0578* |  | putative membrane protein | 0.55 | 1.26 | -0.72 | 0.39 |
| *cg0582* | *rpsG* | 30S ribosomal protein S7 | -0.21 | 1.04 | -1.32 | 0.18 |
| *cg0671* |  | hypothetical protein | 0.84 | 1.89 | -0.17 | 0.85 |
| *cg0689* |  | hypothetical protein | 0.54 | 1 | 0.19 | 0.65 |
| *cg0791* | *pyc* | pyruvate carboxylase | 0.70 | 1.21 | 0.42 | 0.74 |
| *cg0792* |  | hypothetical protein | 0.55 | 1.68 | -0.24 | 0.35 |
| *cg0801* |  | hypothetical protein | -0.20 | 1.45 | 0.66 | 0.21 |
| *cg0817* | *kup* | putative secondary K+ uptake permease, K+ uptake permease (KUP) family | 0.35 | 1.07 | -0.19 | 0.69 |
| *cg1009* |  | putative cyanate permease, MFS-type | 0.29 | 1.01 | 0.32 | 0.59 |
| *cg1032* |  | putative transcriptional regulator, ArsR-family | 0.15 | 1.42 | 0.08 | 0.71 |
| *cg1033* |  | putative secondary Cd2+ transporter, cadmium resistance (CadD) family | 0.45 | 1.51 | 0.08 | 0.77 |
| *cg1105* | *lysI* | low capacity L-Lysine antiporter | 0.32 | 1.01 | -0.72 | 0.29 |
| *cg1116* | *tdcB* | threonine dehydratase | 0.31 | 1.05 | 0.07 | 0.47 |
| *cg1248* |  | putative GTPase, probably involved in stress response | 0.62 | 1.16 | -0.05 | 0.79 |
| *cg1277* |  | putative membrane protein | 0.92 | 1.22 | 0.14 | 0.96 |
| *cg1278* |  | putative secreted protein | 0.59 | 1.06 | 0.02 | 0.9 |
| *cg1432* | *ilvD* | dihydroxy-acid dehydratase | 0.66 | 1.11 | 0.46 | 0.88 |
| *cg1436* | *ilvN* | acetohydroxy acid synthase (AHAS), small subunit | 0.36 | 1.22 | 0.02 | 0.83 |
| *cg1526* |  | putative multidrug efflux permease, MFS-type | 0.74 | 1.39 | 0.63 | 1.25 |
| *cg1626* |  | hypothetical protein | 0.72 | 1.66 | -1.18 | 0.85 |
| *cg1628* |  | putative hydrolase, alpha/beta superfamily | 0.77 | 1.87 | -0.97 | 0.87 |
| *cg1684* | *tatC* | putative twin arginine targeting (Tat) Preprotein translocase subunit | 0.66 | 1.33 | -0.04 | 0.32 |
| *cg1685* | *tatX* | putative twin arginine targeting (Tat) Preprotein translocase subunit | 0.65 | 1.22 | 0.29 | 0.52 |
| *cg1719* | *tetB* | ABC-type multidrug transport system, ATPase and permease subunit | 0.62 | 1.01 | 0.41 | 0.7 |
| *cg1720* | *tetA* | ABC-type multidrug transport system, ATPase and permease subunit | 0.57 | 1.13 | 0.49 | 0.78 |
| *cg1778* | *zwf* | glucose-6-phosphate 1-dehydrogenase | 0.64 | 1.03 | 0.61 | 0.94 |
| *cg1798* | *ribA* | putative GTP cyclohydrolase II/3,4-dihydroxy-2-butanone-4-phosphatesynthase | 0.51 | 1.01 | 0.3 | 0.91 |
| *cg1842* |  | putative secreted metalloprotease | 0.79 | 1.19 | 0.58 | 0.44 |
| *cg2077* |  | putative membrane protein | 0.70 | 1.21 | 0.74 | 0.79 |
| *cg2140* | *recX* | putative regulatory protein RecX | 0.45 | 1.34 | 0.06 | 0.7 |
| *cg2185* | *proS* | prolyl-tRNA synthetase | 0.43 | 1.21 | -0.05 | 0.52 |
| *cg2383* | *metF* | 5,10-Methylenetetrahydrofolate reductase (NAD(P)H) | 0.32 | 1.21 | 0.49 | 0.71 |
| *cg2514* |  | hypothetical protein | 0.52 | 1.2 | -0.5 | 0.87 |
| *cg2515* | *dnaJ2* | Zn-finger containing chaperone | 0.69 | 1.41 | -0.09 | 0.92 |
| *cg2568* | *dctM* | putative secondary C4-dicarboxylate transporter permease, tripartite ATP-independent transporter (TRAP-T) family | 0.80 | 1.15 | 0.51 | 0.8 |
| *cg2573* | *rpsT* | 30S ribosomal protein S20 | -0.31 | 1.04 | -1.33 | 0.23 |
| *cg2584* | *nadD* | nicotinate-nucleotide adenylyltransferase | 0.50 | 1 | -0.15 | 0.45 |
| *cg2617* | *vanB* | vanillate O-demethylase oxidoreductase | 0.48 | 1.09 | 0.22 | 0.29 |
| *cg2633* |  | putative restriction endonuclease | 0.98 | 1.3 | 0.49 | 0.8 |
| *cg2657* |  | putative membrane protein, putative pseudogene | 0.80 | 1.28 | -0.2 | 0.8 |
| *cg2730* |  |  | -0.10 | 1.3 | -1.01 | 0.51 |
| *cg2739* |  | putative multidrug efflux permease, MFS-type | 0.42 | 1.53 | -1.07 | 0.12 |
| *cg2761* |  | metal-dependent hydrolase of the beta-lactamase superfamily III | 0.72 | 1.5 | -0.14 | 0.39 |
| *cg2807* | *tnp11a* | transposase | 0.28 | 1.03 | -0.08 | 0.32 |
| *cg2869* |  | putative deacetylase | 0.86 | 1.31 | 0.75 | 0.85 |
| *cg3094* |  | hypothetical protein | 0.49 | 1.22 | 0.59 | 0.48 |
| *cg3133* |  | ABC-type putative cobalt/sugar transporter, ATPase subunit | 0.70 | 1.14 | -1.07 | 0.08 |
| *cg3134* |  | ABC-type putative cobalt/sugar transporter, permease subunit | 0.44 | 1.14 | -0.8 | 0.45 |
| *cg3301* |  | putative sugar/metabolite permease, MFS-type | 0.64 | 1.13 | 0.51 | 0.53 |
| *cg3330* |  | putative secreted protein | 0.76 | 1.99 | 0.32 | 0.44 |
| *cg3359* | *trpE* | anthranilate synthase subunit I | 0.88 | 2.03 | 0.63 | 0.06 |
| *cg3360* | *trpG* | anthranilate synthase subunit II | 0.77 | 2.11 | 0.47 | 0.21 |
| *cg3361* | *trpD* | anthranilate phosphoribosyltransferase | 0.92 | 1.92 | 0.6 | 0.27 |
| *cg3362* | *trpCF* | phosphoribosylanthranilate isomerase | 0.86 | 1.65 | 0.35 | 0.33 |
| *cg3401* |  | hypothetical protein | 0.75 | 2.06 | 1 | 0.66 |
| *cg3432* | *rpmH* | 50S ribosomal protein L34 | 0.14 | 1.01 | -0.99 | 0.15 |
| *cg4017* | *aroR* | tyrosine and phenylalanine-containing leader peptide | 0.28 | 1.24 | -0.09 | 0.54 |

Table S11: Genes exclusively down-regulated in the (p)ppGpp^0^-strain CR099 Δ*rel*Δ*relS*Δ*relH* after 15 minutes of total starvation. Comparative listing of M-values for both parental strain CR099 (WT) and (p)ppGpp^0^-strain CR099 Δ*rel*Δ*relS*Δ*relH* (Mut), as well as stress exposure durations (t_15_; t_60_), compared to the respective unstressed initial state.

| Locus tag | Gene name | Product/Function | M-value | | | |
| --- | --- | --- | --- | --- | --- | --- |
|  |  |  | **WT15** | **Mut15** | **WT60** | **Mut60** |
| *cg0012* | *ssuR* | transcriptional activator of sulfonate(ester) utilization, ROK-family | -0.59 | -1.13 | 5.57 | 0.19 |
| *cg0016* |  | putative integral membrane protein | -0.70 | -1.22 | -0.57 | -0.64 |
| *cg0039* |  | putative transcriptional regulator | -0.59 | -1.22 | -0.43 | -0.27 |
| *cg0052* |  | ABC-type putative iron-siderophore transporter, permease subunit | -0.55 | -1.02 | 0.24 | -0.54 |
| *cg0075* |  | hypothetical protein | -0.32 | -1.11 | 0.06 | 0.15 |
| *cg0085* | *phoH1* | NYN ribonuclease and ATPase of PhoH family | -0.18 | -1.06 | 0.56 | -0.64 |
| *cg0091* |  | putative D-isomer specific 2-hydroxyacid dehydrogenase | -0.72 | -1.01 | 0.01 | -0.21 |
| *cg0112* | *ureR* | putative transcriptional regulator, MarR-family | -0.30 | -1.19 | -0.61 | -0.4 |
| *cg0124* | *amn* | AMP nucleosidase | -0.80 | -1.35 | -0.18 | -0.65 |
| *cg0128* |  | putative secreted protein | -0.83 | -1.03 | 0 | -0.89 |
| *cg0129* | *putA* | proline dehydrogenase/delta-1-pyrroline-5-carboxylate dehydrogenase | -0.07 | -1.27 | 0.65 | -0.6 |
| *cg0154* |  | putative hydrolase | -0.69 | -1.45 | -0.31 | -0.63 |
| *cg0155* |  | conserved hypothetical protein, carbonic anhydrases/acetyltransferases (isoleucine patch superfamily) | -0.59 | -1.38 | -0.41 | -0.74 |
| *cg0156* | *cysR* | transcriptional activator of assimilatory sulfate reduction, ROK-family | -0.91 | -1.58 | 2.59 | -0.1 |
| *cg0220* |  | putative acetyltransferase, GNAT-family | -0.30 | -1.25 | -0.22 | -0.31 |
| *cg0387* |  | putative NAD/mycothiol-dependent formaldehyde dehydrogenase | -0.85 | -1.31 | -0.01 | -0.58 |
| *cg0388* |  | putative Zn-dependent hydrolase | -0.88 | -1.18 | -0.18 | -0.46 |
| *cg0408* |  | putative membrane protein | -0.18 | -1.22 | -0.45 | -0.92 |
| *cg0418* |  | putative aminotransferase, involved in cell wall biosynthesis | -0.34 | -1.21 | -0.27 | -0.85 |
| *cg0419* |  | putative glycosyltransferase | -0.57 | -1.17 | -0.47 | -0.84 |
| *cg0469* |  | ABC-type putative hemin transporter, ATPase subunit | -0.14 | -1.11 | -0.49 | -0.64 |
| *cg0483* | *cgtS4* | two-component system, sensory histidine kinase | -0.50 | -1.13 | -0.6 | -0.92 |
| *cg0662* |  | hypothetical protein, putative FAD/FMN-containing dehydrogenase | -0.52 | -1.17 | -0.14 | -0.62 |
| *cg0663* | *cma* | cyclopropane-fatty-acyl-phospholipid synthase | -0.51 | -1.13 | 0 | -0.44 |
| *cg0717* | *crtEb* | lycopene elongase | -0.48 | -1.12 | -0.04 | -0.44 |
| *cg0718* | *crtYf* | C50 carotenoid epsilon cyclase | -0.70 | -1.49 | 0.04 | -0.63 |
| *cg0719* | *crtYe* | C50 carotenoid epsilon cyclase | -0.93 | -1.48 | -0.08 | -0.37 |
| *cg0720* | *crtI2* | phytoene dehydrogenase (desaturase) | -0.70 | -1.47 | -0.01 | -0.49 |
| *cg0721* | *crtB2* | phytoene synthetase | -0.39 | -1.42 | 0.04 | -0.14 |
| *cg0722* |  | putative multidrug efflux protein, resistance-nodulation-cell division (RND) superfamily | -0.30 | -1.19 | 0.17 | 0.16 |
| *cg0796* | *prpD1* | putative (2-methyl) citrate dehydratase | 0.31 | -1.06 | 2.09 | 0.33 |
| *cg0797* | *prpB1* | putative (methyl)isocitrate lyase | 0.39 | -1.37 | 2.27 | 0.16 |
| *cg0798* | *prpC1* | putative (methyl)citrate synthase | 0.67 | -1.39 | 2.3 | 0.03 |
| *cg0807* |  | hypothetical protein | -0.68 | -1.74 | 0.21 | -0.06 |
| *cg0838* |  | putative helicase | 0.98 | -1.3 | 1.74 | 0.29 |
| *cg0842* |  | putative DNA helicase | -0.20 | -1.98 | 0 | -0.56 |
| *cg0843* |  | putative helicase | -0.58 | -2.05 | -0.07 | -0.63 |
| *cg0844* |  | type II restriction enzyme, methylase subunit | -0.31 | -1.55 | -0.06 | -0.28 |
| *cg0911* |  | putative inositol monophosphatase | -0.88 | -1.11 | -0.64 | -0.77 |
| *cg0973* | *pgi* | glucose-6-phosphate isomerase | -0.43 | -1 | 0.17 | -0.27 |
| *cg1068* |  | putative oxidoreductase | -0.79 | -1.01 | 0.05 | -0.12 |
| *cg1081* |  | ABC-type putative daunorubicin transporter, ATPase subunit | -0.61 | -1.14 | -0.77 | -0.81 |
| *cg1082* |  | putative membrane protein | -0.60 | -1.38 | -0.63 | -0.56 |
| *cg1083* | *cgtS10* | two-component system, sensory histidine kinase | -0.49 | -1.05 | -0.42 | -0.57 |
| *cg1089* |  | ABC-type putative multidrug transporter, ATPase and permease subunit | -0.36 | -1.05 | 0.5 | -0.51 |
| *cg1106* |  | hypothetical protein | -0.33 | -1.12 | 0.68 | 0.15 |
| *cg1145* | *fum* | fumarate hydratase | -0.77 | -1.03 | -0.85 | -0.59 |
| *cg1179* |  | putative membrane protein | -0.30 | -1 | 0.39 | -0.31 |
| *cg1206* |  | hypothetical protein | -0.66 | -1.14 | -0.06 | -0.37 |
| *cg1224* |  | hypothetical protein | -0.76 | -1.41 | 1.14 | 0.49 |
| *cg1236* | *tpx* | thiol peroxidase | -0.90 | -1.39 | -0.36 | -0.6 |
| *cg1262* | *folP2* | dihydropteroate synthase | -0.51 | -1.01 | -0.35 | -0.65 |
| *cg1263* |  | glycosyltransferase, involved in cell wall biogenesis | -0.58 | -1.15 | -0.45 | -0.74 |
| *cg1285* |  | hypothetical protein | -0.57 | -1.02 | -0.62 | -0.57 |
| *cg1348* |  | putative membrane protein | -0.09 | -1.26 | -0.22 | 0.41 |
| *cg1349* |  | putative membrane protein | 0.07 | -1.1 | -0.25 | 0.24 |
| *cg1393* |  | putative acetyltransferase, GNAT family | -0.70 | -1.09 | -0.14 | -0.49 |
| *cg1478* |  | hypothetical protein | -0.28 | -1.13 | 2.32 | 0.78 |
| *cg1618* |  | hypothetical protein | -0.95 | -1.33 | -0.03 | -0.36 |
| *cg1619* |  | putative transcriptional regulator, AraC family | -0.65 | -1.37 | 0.55 | -0.19 |
| *cg1686* |  | putative transcriptional regulatory protein | -0.01 | -1.02 | 0.29 | 0.04 |
| *cg1702* |  | hypothetical protein | -0.62 | -1.07 | 0.29 | -0.29 |
| *cg1789* | *tpi* | triosephosphate isomerase | -0.54 | -1.01 | -0.33 | -0.51 |
| *cg2110* |  | putative membrane protein | -0.94 | -1.49 | -0.45 | -0.8 |
| *cg2162* | *thyX* | thymidylate synthase | -0.78 | -1.12 | -0.88 | -0.72 |
| *cg2163* | *dapB* | dihydrodipicolinate reductase | -0.72 | -1.18 | -0.69 | -0.68 |
| *cg2181* |  | ABC-type putative dipeptide/oligopeptide transporter, substrate-binding lipoprotein | 0.29 | -1.01 | 0.77 | -0.54 |
| *cg2270* |  | hypothetical protein | 0.36 | -1.2 | -0.15 | -0.96 |
| *cg2271* |  | putative secondary Co2+/Zn2+/Cd2+ efflux transporter, cation diffusion facilitator (CDF) family | 0.55 | -1.4 | 0.02 | -0.4 |
| *cg2324* |  | hypothetical protein | -0.36 | -1.04 | -0.16 | -0.82 |
| *cg2342* |  | putative oxidoreductase | -0.49 | -1.23 | 0.29 | -0.46 |
| *cg2350* |  | hypothetical protein | -0.74 | -1.24 | -0.94 | -0.67 |
| *cg2435* |  | putative ss-mRNA endonuclease | -0.72 | -1.01 | -0.35 | -0.91 |
| *cg2530* | *treX* | putative trehalose synthase | -0.57 | -1.31 | -0.07 | -0.33 |
| *cg2558* |  | putative aldose 1-epimerase | -0.62 | -1.07 | 0.14 | 0.09 |
| *cg2587* |  | putative dehydrogenase | -0.45 | -1.07 | -0.12 | -0.2 |
| *cg2621* |  | hypothetical protein | -0.82 | -1.34 | -0.38 | -0.45 |
| *cg2663* |  | hypothetical protein | -0.71 | -1.72 | -0.37 | -0.98 |
| *cg2676* |  | ABC-type putative dipeptide/oligopeptide transporter, permease subunit | -0.96 | -2.45 | 4.63 | -1.07 |
| *cg2678* |  | ABC-type putative dipeptide/oligopeptide transporter, substrate-binding lipoprotein | -0.77 | -2.66 | 4.99 | -0.43 |
| *cg2679* |  | hypothetical protein | -0.43 | -1.19 | 2.5 | -0.58 |
| *cg2687* | *metB* | cystathionine gamma-synthase | -0.81 | -1.32 | 2.52 | 0.64 |
| *cg2707* |  | hypothetical protein | -0.51 | -1.25 | 0.43 | -0.46 |
| *cg2708* | *msiK1* | ABC-type putative sugar transporter, ATPase subunit | -0.32 | -1.31 | 0.75 | -0.2 |
| *cg2727* |  | putative secreted protein | -0.96 | -1.41 | -0.3 | -0.68 |
| *cg2867* | *gpx* | putative mycothiol peroxidase, GSH peroxidase family | -0.92 | -1.23 | 0.11 | -0.21 |
| *cg2889* |  | putative transcriptional regulator, MerR family | -0.34 | -1.37 | 0.07 | -0.52 |
| *cg2890* |  | hypothetical protein, putative amino acid processing enzyme | -0.32 | -1.02 | 0.5 | -0.21 |
| *cg2908* |  | putative membrane protein | -0.33 | -1.19 | -0.8 | -0.97 |
| *cg2953* |  | putative 4-hydroxybenzaldehyde dehydrogenase | -0.96 | -1.87 | 0.68 | -0.32 |
| *cg2958* | *butA* | L-2,3-butanediol dehydrogenase/acetoin reductase | -0.79 | -1.13 | 0.09 | -0.81 |
| *cg2978* |  | putative membrane protein | 0.03 | -1.11 | -0.33 | -0.28 |
| *cg2991* |  | putative membrane protein | -0.92 | -1.29 | -0.86 | -0.94 |
| *cg2992* |  | putative secreted protein | -0.70 | -1 | -0.57 | -0.77 |
| *cg3012* |  | putative membrane protein | 0.00 | -1.18 | -0.06 | -0.09 |
| *cg3015* |  | hypothetical protein | -0.40 | -1.66 | -0.1 | -0.38 |
| *cg3016* |  | hypothetical protein | -0.19 | -1.67 | -0.01 | -0.23 |
| *cg3017* |  | putative membrane protein | -0.08 | -1.47 | 0.08 | -0.12 |
| *cg3068* | *fda* | fructose-bisphosphate aldolase | -0.56 | -1.26 | -0.35 | -0.79 |
| *cg3097* | *hspR* | putative transcriptional regulator, MerR-family | -0.89 | -1.16 | -0.71 | 0.88 |
| *cg3098* | *dnaJ* | chaperone DnaJ, heat shock protein | -0.74 | -1.1 | -0.69 | 0.79 |
| *cg3099* | *grpE* | chaperone GrpE, heat shock protein | -0.93 | -1.38 | -0.48 | 1.06 |
| *cg3100* | *dnaK* | chaperone DnaK, heat shock protein | -0.71 | -1.15 | -0.39 | 0.71 |
| *cg3103* |  | hypothetical protein | 0.01 | -2.26 | -0.01 | -0.77 |
| *cg3104* |  | hypothetical protein | 0.59 | -1.4 | 0.07 | -0.18 |
| *cg3112* | *cysZ* | sulfate transporter | -0.60 | -1.98 | 8.3 | -1.01 |
| *cg3113* | *cysY* | sirohydrochlorin ferrochelatase | -0.72 | -1.97 | 8.66 | -0.96 |
| *cg3115* | *cysD* | sulfate adenylyltransferase subunit 2 | -0.85 | -2.85 | 8 | -0.52 |
| *cg3119* | *fpr2* | ferredoxin-NADP(+) reductase | -0.55 | -2.56 | 9.18 | 0.77 |
| *cg3122* |  | hypothetical protein, PhnB-family | 0.22 | -1.07 | 1.7 | 0.66 |
| *cg3131* |  | acetylornithine deacetylase | -0.77 | -1.4 | 2.39 | -0.67 |
| *cg3159* |  | putative universal stress protein | -0.52 | -1.19 | 0.27 | -0.76 |
| *cg3218* |  | pyruvate kinase-like protein | -0.64 | -1.22 | -0.23 | -0.83 |
| *cg3219* | *ldh* | L-Lactate dehydrogenase | -0.74 | -1.24 | -0.67 | -0.75 |
| *cg3223* |  | hypothetical protein, putative FMN reductase | -0.48 | -1.09 | -0.21 | -0.84 |
| *cg3352* | *nagR* | transcriptional activator of gentisate catabolism, IclR-family | -0.05 | -1.06 | 1.01 | 0.87 |
| *cg3412* | *azlD* | putative permease, branched-chain amino acid permease (azaleucine resistance) | -0.70 | -1.05 | -0.84 | -0.61 |
| *cg3418* |  | putative secreted protein | 0.16 | -1.06 | -0.08 | -0.47 |

Table S12: Genes exclusively up-regulated in the (p)ppGpp^0^-strain CR099 Δ*rel*Δ*relS*Δ*relH* after 60 minutes of total starvation. Comparative listing of M-values for both parental strain CR099 (WT) and (p)ppGpp^0^-strain CR099 Δ*rel*Δ*relS*Δ*relH* (Mut), as well as stress exposure durations (t_15_; t_60_), compared to the respective unstressed initial state.

| Locus tag | Gene name | Product/Function | M-value | | | |
| --- | --- | --- | --- | --- | --- | --- |
|  |  |  | **WT15** | **Mut15** | **WT60** | **Mut60** |
| *cg0314* | *brnF* | secondary branched-chain amino acid efflux transporter, LIV-E family, large subunit | 0.35 | -0.02 | 0.32 | 1.19 |
| *cg0690* | *groES* | 10kDa chaperonin | 0.92 | -0.24 | 0.69 | 1.31 |
| *cg1282* |  | hypothetical protein | 0.34 | 0.65 | 0.33 | 1.05 |
| *cg1295* |  | putative hydrolase/acyltransferase, alpha/beta hydrolase superfamily | 0.98 | 0.8 | 0.81 | 2.12 |
| *cg1505* |  | putative secreted protein | 0.37 | 0.83 | 0.86 | 1.38 |
| *cg1560* | *uvrA* | excinuclease ABC, ATPase subunit A | 0.54 | 0.91 | 0.07 | 1.01 |
| *cg1606* | *pyrG* | CTP synthetase | 0.63 | 0.52 | 0.56 | 1.55 |
| *cg1688* | *pafA2* | putative proteasome component | 0.69 | 0.76 | 0.76 | 1.15 |
| *cg1689* | *pup* | prokaryotic ubiquitin-like protein | 0.75 | 0.92 | 0.82 | 1.43 |
| *cg1780* | *pgl* | 6-Phosphogluconolactonase | 0.59 | 0.43 | 0.87 | 1.07 |
| *cg1781* |  | putative FAD-dependent oxidoreductase, pseudogene (C-terminal fragment) | 0.38 | 0.7 | 0.45 | 1.74 |
| *cg1852* | *sdaA* | L-Serine dehydratase | 0.05 | 0.24 | 0.58 | 1.24 |
| *cg1860* |  | putative membrane protein | -0.33 | 0.07 | 0.15 | 1.25 |
| *cg2174* |  | exopolyphosphatase-related protein | 0.55 | 0.9 | 0.6 | 1.12 |
| *cg2244* |  | lyase, class I family | 0.22 | 0.92 | -0.12 | 1.09 |
| *cg2258* | *glnD* | putative protein PII uridylyltransferase | 0.21 | -0.26 | 0.15 | 1.44 |
| *cg2260* | *glnK* | nitrogen regulatory protein PII | 0.42 | 0.61 | 0.46 | 2.59 |
| *cg2359* | *ileS* | isoleucine-tRNA ligase | -0.12 | -0.53 | -0.48 | 1.13 |
| *cg2418* | *ilvE* | branched-chain amino acid aminotransferase, AT class III | 0.67 | 0.64 | 0.66 | 1.01 |
| *cg2619* |  | putative secondary malonate transporter, auxin efflux carrier (AEC) family | 0.21 | 0.72 | -0.21 | 1.01 |
| *cg2644* | *clpP2* | endopeptidase Clp, proteolytic subunit | 0.33 | 0.9 | 0.44 | 1.06 |
| *cg2645* | *clpP1* | endopeptidase Clp, proteolytic subunit | 0.41 | 0.92 | 0.29 | 1.02 |
| *cg2661* |  | hypothetical protein | 0.35 | 0.71 | 0.96 | 1.04 |
| *cg2963* | *clpC* | putative ATP-dependent protease (heat shock protein) | 0.33 | 0.75 | -0.17 | 1.21 |
| *cg3077* |  | putative membrane protein | 0.05 | -0.43 | -0.23 | 1.65 |
| *cg3078* |  | hypothetical protein | 0.07 | -0.08 | 0.03 | 1.74 |
| *cg3079* | *clpB* | putative ATP-dependent protease (heat shock protein) | 0.34 | 0.2 | 0.45 | 1.85 |
| *cg3085* |  | putative monooxygenase | 0.44 | 0.72 | 0.8 | 1.13 |
| *cg3099* | *grpE* | chaperone GrpE, heat shock protein | -0.93 | -1.38 | -0.48 | 1.06 |
| *cg3274* |  | putative DNA invertase, putative pseudogene | 0.46 | 0.57 | 0.42 | 1.01 |
| *cg3275* | *fdxA* | putative ferredoxin | 0.74 | 0.64 | 0.69 | 1.12 |
| *cg3337* |  | putative membrane protein | 0.60 | 0.76 | 0.42 | 1.24 |
| *cg3365* | *rmpC* | phosphotransferase system (PTS), putative ribitol/mannitol-specific enzyme IIC component | 0.78 | 0.89 | 0.98 | 1.03 |

Table S13: Genes exclusively down-regulated in the (p)ppGpp^0^-strain CR099 Δ*rel*Δ*relS*Δ*relH* after 15 minutes of total starvation. Comparative listing of M-values for both parental strain CR099 (WT) and (p)ppGpp^0^-strain CR099 Δ*rel*Δ*relS*Δ*relH* (Mut), as well as stress exposure durations (t_15_; t_60_), compared to the respective unstressed initial state.

| Locus tag | Gene name | Product/Function | M-value | | | |
| --- | --- | --- | --- | --- | --- | --- |
|  |  |  | **WT15** | **Mut15** | **WT60** | **Mut60** |
| *cg0355* |  | putative NTP pyrophosphohydrolase | -0.47 | -0.92 | -0.84 | -1.14 |
| *cg0364* |  | putative membrane protein | -0.38 | -1.04 | 0.06 | -1.31 |
| *cg0414* | *wzz* | cell surface polysaccharide biosynthesis/chain length determinant | -0.28 | -0.51 | -0.69 | -1 |
| *cg0446* | *sdhA* | succinate dehydrogenase, subunit A | -0.39 | -0.68 | -0.43 | -1.03 |
| *cg0933* |  | DNA/RNA helicase, superfamily II | -0.38 | -0.54 | -0.82 | -1.07 |
| *cg0982* |  | putative membrane protein | -0.30 | -0.82 | -0.36 | -1.01 |
| *cg1036* |  | hypothetical protein | -0.19 | -0.39 | -0.57 | -1.44 |
| *cg1069* | *gapX* | putative glyceraldehyde-3-phosphate dehydrogenase | -0.37 | -0.96 | -0.66 | -1.48 |
| *cg1654* | *thiD1* | phosphomethylpyrimidine kinase/thiamine-phosphate diphosphorylase | -0.55 | -0.87 | -0.67 | -1.16 |
| *cg1655* | *thiM* | hydroxyethylthiazole kinase | -0.39 | -0.57 | -0.46 | -1.03 |
| *cg1867* | *secD* | preprotein translocase, SecD subunit | -0.32 | -0.36 | -0.7 | -1.23 |
| *cg2237* | *thiO* | putative D-amino acid dehydrogenase, small subunit | -0.35 | -0.66 | -0.82 | -1 |
| *cg2543* | *glcD* | putative (S)-2-hydroxy-acid oxidase | 0.77 | -0.57 | 0.04 | -1.23 |
| *cg2546* |  | putative secondary C4-dicarboxylate transporter, tripartite ATP-independent transporter (TRAP-T) family | 1.31 | -0.67 | -0.16 | -1.51 |
| *cg2674* |  | alkylhydroperoxidase, AhpD-family | -0.69 | -0.91 | 1.15 | -1.02 |
| *cg2959* |  | putative secreted protein | -0.16 | -0.91 | 0.1 | -1.18 |
| *cg3156* |  | putative secreted protein | -0.77 | -0.96 | -0.51 | -1.3 |

REFERENCES

Blom, J., Kreis, J., Spänig, S., Juhre, T., Bertelli, C., Ernst, C., et al. (2016). EDGAR 2.0: An enhanced software platform for comparative gene content analyses. *Nucleic acids research* 44, W22-8. doi: 10.1093/nar/gkw255

Brown, N. P., Leroy, C., and Sander, C. (1998). MView: A web-compatible database search or multiple alignment viewer. *Bioinformatics (Oxford, England)* 14, 380–381.

Brune, I., Werner, H., Hüser, A. T., Kalinowski, J., Pühler, A., and Tauch, A. (2006). The DtxR protein acting as dual transcriptional regulator directs a global regulatory network involved in iron metabolism of Corynebacterium glutamicum. *BMC Genomics* 7, 21. doi: 10.1186/1471-2164-7-21

Busche, T., Silar, R., Pičmanová, M., Pátek, M., and Kalinowski, J. (2012). Transcriptional regulation of the operon encoding stress-responsive ECF sigma factor SigH and its anti-sigma factor RshA, and control of its regulatory network in Corynebacterium glutamicum. *BMC Genomics* 13, 445. doi: 10.1186/1471-2164-13-445

Ehira, S., Shirai, T., Teramoto, H., Inui, M., and Yukawa, H. (2008). Group 2 sigma factor SigB of Corynebacterium glutamicum positively regulates glucose metabolism under conditions of oxygen deprivation. *Applied and environmental microbiology* 74, 5146–5152. doi: 10.1128/AEM.00944-08

Hilker, R., Stadermann, K. B., Schwengers, O., Anisiforov, E., Jaenicke, S., Weisshaar, B., et al. (2016). ReadXplorer 2-detailed read mapping analysis and visualization from one single source. *Bioinformatics (Oxford, England)* 32, 3702–3708. doi: 10.1093/bioinformatics/btw541

Keilhauer, C., Eggeling, L., and Sahm, H. (1993). Isoleucine synthesis in *Corynebacterium glutamicum*: Molecular analysis of the ilvB-ilvN-ilvC operon. *J. Bacteriol.* 175, 5595–5603. doi: 10.1128/jb.175.17.5595-5603.1993

Koch, D. J., Rückert, C., Albersmeier, A., Hüser, A. T., Tauch, A., Pühler, A., et al. (2005). The transcriptional regulator SsuR activates expression of the Corynebacterium glutamicum sulphonate utilization genes in the absence of sulphate. *Mol Microbiol* 58, 480–494. doi: 10.1111/j.1365-2958.2005.04836.x

Love, M. I., Huber, W., and Anders, S. (2014). Moderated estimation of fold change and dispersion for RNA-seq data with DESeq2. *Genome biology* 15, 550. doi: 10.1186/s13059-014-0550-8

Myronovskyi, M., Tokovenko, B., Brötz, E., Rückert, C., Kalinowski, J., and Luzhetskyy, A. (2014). Genome rearrangements of Streptomyces albus J1074 lead to the carotenoid gene cluster activation. *Applied microbiology and biotechnology* 98, 795–806. doi: 10.1007/s00253-013-5440-6

Notredame, C., Higgins, D. G., and Heringa, J. (2000). T-Coffee: A novel method for fast and accurate multiple sequence alignment. *Journal of molecular biology* 302, 205–217. doi: 10.1006/jmbi.2000.4042

Pfeifer-Sancar, K., Mentz, A., Rückert, C., and Kalinowski, J. (2013). Comprehensive analysis of the *Corynebacterium glutamicum* transcriptome using an improved RNAseq technique. *BMC Genomics* 14, 888. doi: 10.1186/1471-2164-14-888

Rey, D. A., Nentwich, S. S., Koch, D. J., Rückert, C., Pühler, A., Tauch, A., et al. (2005). The McbR repressor modulated by the effector substance S-adenosylhomocysteine controls directly the transcription of a regulon involved in sulphur metabolism of Corynebacterium glutamicum ATCC 13032. *Molecular microbiology* 56, 871–887. doi: 10.1111/j.1365-2958.2005.04586.x

Rückert, C., Milse, J., Albersmeier, A., Koch, D. J., Pühler, A., and Kalinowski, J. (2008). The dual transcriptional regulator CysR in Corynebacterium glutamicum ATCC 13032 controls a subset of genes of the McbR regulon in response to the availability of sulphide acceptor molecules. *BMC Genomics* 9, 483. doi: 10.1186/1471-2164-9-483

Schneefeld, M., Busche, T., Geffers, R., Kalinowski, J., and Bange, F.-C. (2017). The transcriptional regulator LysG (Rv1985c) of Mycobacterium tuberculosis activates lysE (Rv1986) in a lysine-dependent manner. *PloS one* 12, e0186505. doi: 10.1371/journal.pone.0186505

Toyoda, K., and Inui, M. (2016). The extracytoplasmic function σ factor σ(C) regulates expression of a branched quinol oxidation pathway in Corynebacterium glutamicum. *Molecular microbiology* 100, 486–509. doi: 10.1111/mmi.13330

Wolf, T., Schneiker-Bekel, S., Neshat, A., Ortseifen, V., Wibberg, D., Zemke, T., et al. (2017). Genome improvement of the acarbose producer Actinoplanes sp. SE50/110 and annotation refinement based on RNA-seq analysis. *Journal of biotechnology* 251, 112–123. doi: 10.1016/j.jbiotec.2017.04.013
